# Super-resolution RESOLFT microscopy of lipid bilayers using a fluorophore-switch dyad†

**DOI:** 10.1039/d0sc02447c

**Published:** 2020-08-10

**Authors:** Andrew T. Frawley, Virginia Wycisk, Yaoyao Xiong, Silvia Galiani, Erdinc Sezgin, Iztok Urbančič, Andreas Vargas Jentzsch, Kathryn G. Leslie, Christian Eggeling, Harry L. Anderson

**Affiliations:** Department of Chemistry, University of Oxford, Chemistry Research Laboratory Oxford OX1 3TA UK harry.anderson@chem.ox.ac.uk; MRC Human Immunology Unit, Weatherall Institute of Molecular Medicine, University of Oxford Oxford OX3 9DS UK christian.eggeling@rdm.ox.ac.uk; SAMS Research Group, Institut Charles Sadron, CNRS–UPR 22, University of Strasbourg Strasbourg Cedex 2 67034 France; Institute of Applied Optics and Biophysics, Friedrich-Schiller-University Jena Max-Wien Platz 4 07743 Jena Germany; Leibniz Institute of Photonic Technology e.V. Albert-Einstein-Straße 9 07745 Jena Germany; Jena Center for Soft Matter (JCSM) Philosophenweg 7 07743 Jena Germany

## Abstract

Dyads consisting of a photochromic switch covalently linked to a fluorescent dye allow the emission from the dye to be controlled by reversible photoisomerization of the switch; one form of the switch quenches fluorescence by accepting energy from the dye. Here we investigate the use of dyads of this type for super-resolution imaging of lipid bilayers. Giant unilamellar vesicles stained with the dyads were imaged with about a two-fold resolution-enhancement compared with conventional confocal microscopy. This was achieved by exciting the fluorophore at 594 nm, using a switch activated by violet and red light (405/640 nm).

## Abstract 2

A photoswitchable quencher can be used to reversibly turn off the emission from a fluorescent dye, generating a small molecule dyad that is effective for super-resolution RESOLFT microscopy.

## Graphical abstract

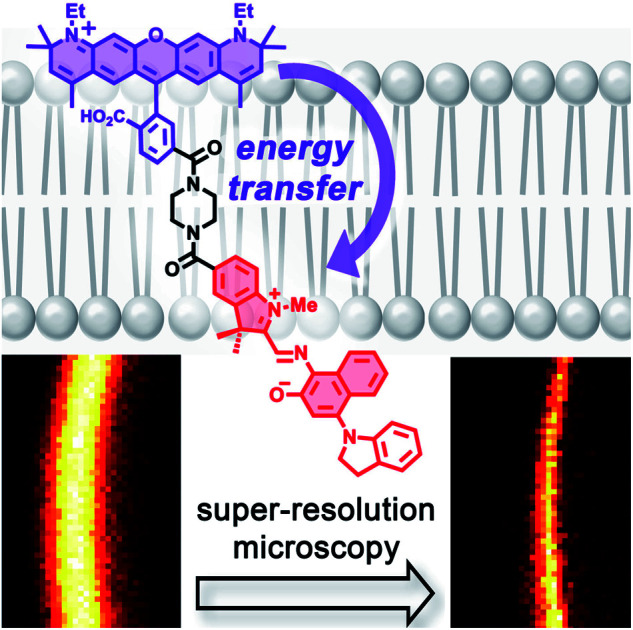

## Introduction

**Fig. 1:**
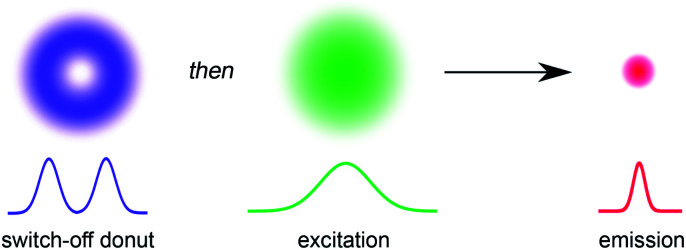
The principle of RESOLFT microscopy. A donut-shaped laser for turning off fluorescence (violet) with zero intensity at the center is spatially concentric with a Gaussian-shaped excitation laser (green). The resulting fluorescent region of the sample (red) is smaller than the diffraction limit.

The ability to resolve biological structures smaller than a few hundred nanometers is critical for the study of spatial organization and dynamics in living cells. Before the invention of super-resolution microscopy (SRM), the spatial resolution of optical microscopes was limited by diffraction. However, there is now a range of SRM techniques that allow sub-diffraction imaging of biological samples.^1–5^ Stimulated emission depletion (STED) is a form of SRM in which a long-wavelength donut-shaped depletion laser is overlaid with the excitation laser, forcing the spatially defined subset of molecules exposed to the donut into a non-emissive state.^6^ Fluorescence is only detected from the central spot, which is not diffraction limited. However, STED requires high laser powers (up to GW cm^−2^) which can cause permanent photobleaching of the fluorophore and photodamage to the sample. Techniques such as triplet relaxation STED,^7^ and scanning methods including RESCue, DyMIN and MINFIELD have been developed to mitigate photodamage.^8,9^ STED belongs to a broader collection of SRM techniques called ‘reversible saturable optical fluorescence transitions' (RESOLFT) microscopy,^10–13^ which require an imaging agent to be switched reversibly over many cycles between fluorescent and non-fluorescent states. Switching the fluorescence using a photochemical reaction requires lower laser intensities than stimulated emission, making the technique potentially less damaging to the sample. Ideally, the photochemical reaction is driven in both directions using visible light (wavelength > 400 nm). UV irradiation should be avoided for imaging biological samples, as phototoxic effects can disturb normal cell function, and microscope optics are typically not optimized for wavelengths shorter than 400 nm. Here we demonstrate that small-molecule dyads, consisting of a switchable quencher connected to a fluorescent dye, can be used for RESOLFT microscopy. A donut-shaped beam at 405 nm is used to switch the system into its dark state, and excitation at 594 nm stimulates fluorescence only from the center of the donut (Fig. 1).

RESOLFT has previously been demonstrated using reversibly switchable fluorescent proteins.^14–21^ However, the applications of fluorescent proteins are limited by low brightness, poor photostability and large size, compared to synthetic dyes,^17^ and they need to be introduced by genetic encoding. Therefore, there is an urgent need for small-molecule reversibly switchable fluorescent dyes for RESOLFT microscopy.^22–24^ A few small-molecule dyes have been demonstrated previously for RESOLFT microscopy.^23–26^ Fluorescent bis-sulfone diarylethenes have been used for RESOLFT, although they rely on UV irradiation (usually 350–365 nm) to switch them to the fluorescent isomer.^23,25,26^ Another example utilizes a Cy3–Cy5 heterodimer which can be switched using visible light, but requires long irradiation times and addition of thiols to enable reversible switching.^24^ The ability to move the switching wavelengths away from the UV towards the visible region is highly desirable.^23,26^

**Fig. 2:**
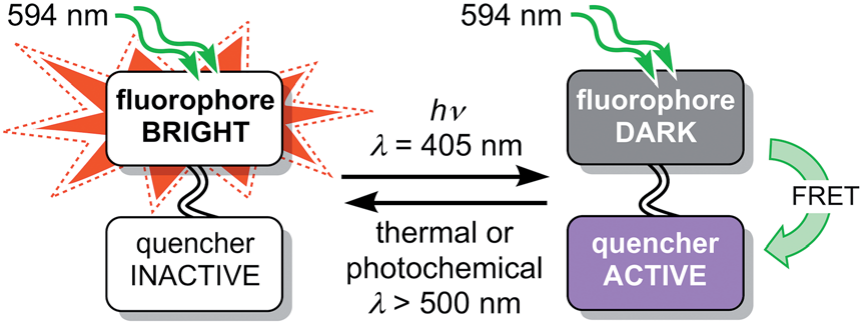
Schematic design of a dyad using a switchable quencher to modulate the fluorescence of the emissive dye. Irradiation at 405 nm switches the quencher to its active form.

The fluorescence of a dye can be reversibly modulated using a covalently-linked photoswitchable quencher.^27–45^ In our system, quenching is achieved by Förster resonance energy transfer (FRET). The dyad can exist in two states: a bright state in which the quencher is inactive and a dark state in which the quencher is activated and quenches the emission from the dye (Fig. 2). Various implementations of this dyad concept have been reported,^28–44^ many of which use diarylethene photoswitches as FRET acceptors, which are converted to their quenching (closed) form by UV irradiation.^29,30,32–34,40,42^ Silica nanoparticles incorporating a rhodamine–diarylethene dyad have been used to demonstrate enhanced spatial resolution in a microscopy line profile using UV switching.^31^ However, there are few photoswitches that can be driven in both directions using wavelengths longer than 400 nm without concomitant loss of their favorable switching properties, such as high conversion in the photostationary states in both directions and suitable switching kinetics.^46^

**Fig. 3:**
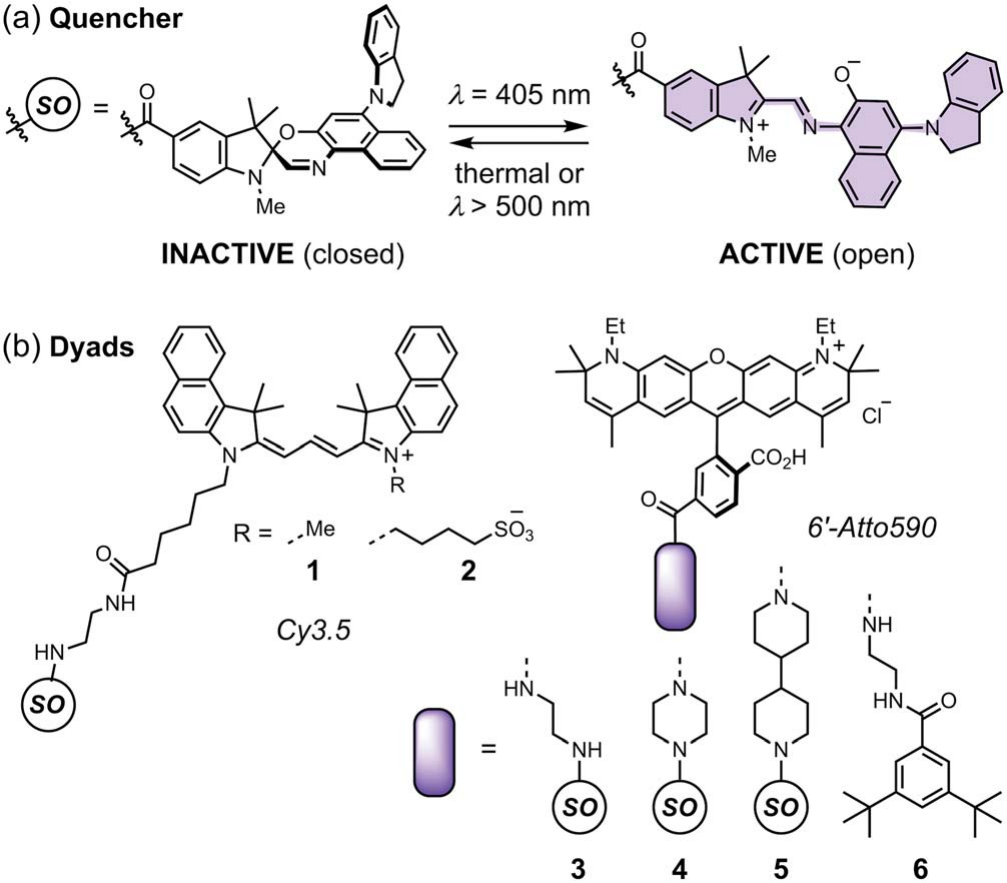
(a) The closed and open structures of the spironaphthoxazine (SO) switch and (b) the structures of dyads **1–5**, and non-switching reference compound **6**.

Spironaphthoxazines are a promising class of photoswitch.^47^ An indoline-substituted spironaphthoxazine (SO) exhibits reversible switching from a yellow spiro form to a deep purple merocyanine upon irradiation with violet light at 405 nm, with a photostationary state comprising around 90% of the open form (Fig. 3a). The colored open form exhibits a broad absorption band at 460–640 nm and closes to the spiro form either thermally, or accelerated by irradiation with visible light >500 nm. The spironaphthoxazine can be switched from the closed to the open form for several hundred cycles with minimal fatigue in organic solvents.^47^

Previously, we synthesized a dyad consisting of the spironaphthoxazine switch covalently linked to Atto565, a rhodamine-type fluorescent dye. This dyad was shown to be taken up by HEK cells and switch between bright and dark states *in cellulo* using confocal microscopy.^47^ In the current work, we have tested a range of related dyads, **1–5** (Fig. 3b), made by attaching a selection of fluorescent dyes (Cy3.5 or Atto590) to the spironaphthoxazine switch. Our choice of dyes was determined by two major factors. Firstly, we require excellent FRET efficiency. Secondly, we require dyes that are efficiently excited using laser wavelengths available on our RESOLFT microscope. The Atto590 and Cy3.5 derivatives are both ideal for excitation with a 594 nm laser. The calculated FRET efficiencies of the dyads are ≥99% (for overlap of the dye emission spectrum with the absorption spectrum of the open form of the switch; ESI Section 4†). Photoinduced electron transfer (PET) may also contribute to the quenching of the fluorophore in these dyads.^28,35–37,39,40,42,43,45,46^ The redox potentials of the components (ESI Section 5†) suggest that PET is thermodynamically favorable, from both the closed and open forms of the spironaphthoxazine to the Atto590 and Cy3.5 fluorophores. We have explored several linker units to study their effect on the molecular properties, but all these linkers are probably too long for efficient PET, and we expect that FRET is the dominant quenching mechanism.

## Results and discussion

Dyads (**1–5**, Fig. 3b) were synthesized from dyes bearing carboxylic acid groups. Amide coupling of Boc-protected amine linkers to the dyes was followed by removal of Boc groups and a second amide coupling to the spironaphthoxazine switch (see ESI Section 2 for Experimental details†). The photophysical properties, such as fluorescence quantum yields and lifetimes, of the dyads and their dye precursors were measured (see ESI Section 3, Fig. S1–S11 and Table S1†). The rigid linkers in dyads **4** and **5** resulted in small increases in fluorescence quantum yield and lifetime compared to dyad **3**.

**Fig. 4:**
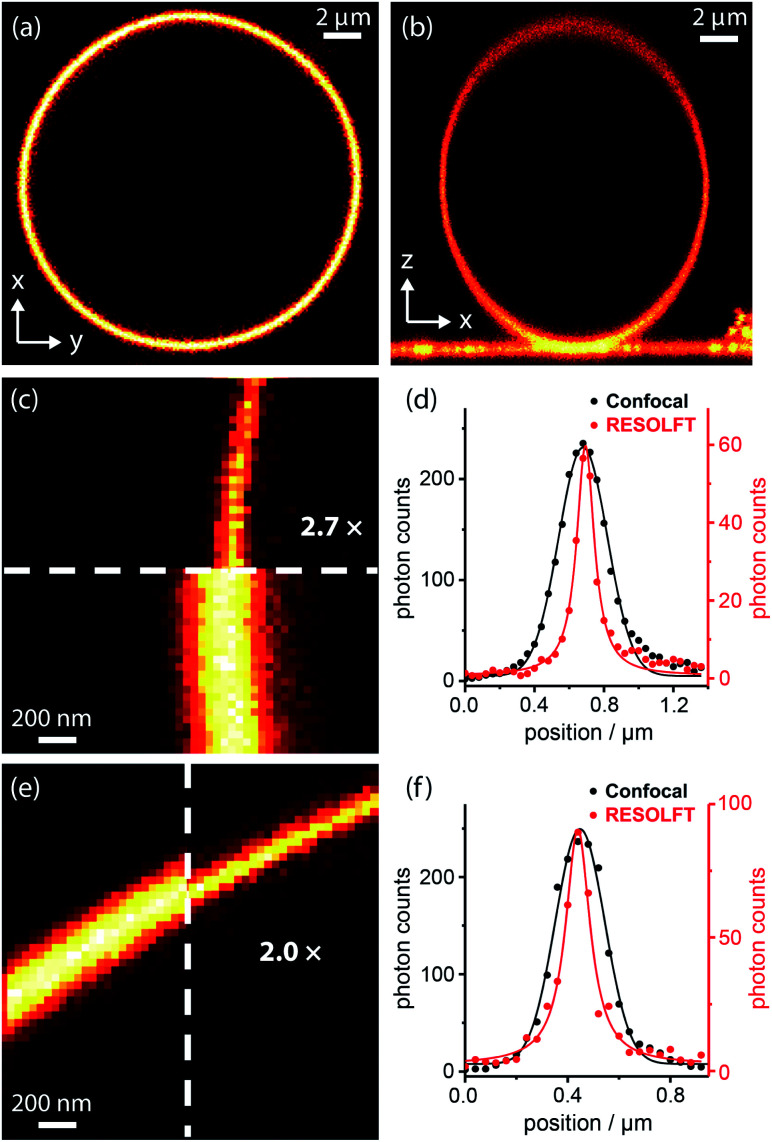
RESOLFT images of DOPC GUV membranes stained with spironaphthoxazine dyads: confocal images of (a) equatorial cross-section (*xy*) and (b) axial cross-section (*xz*) of a whole GUV; (c) confocal and RESOLFT images, scaled from zero to their respective maximum photon counts, and (d) corresponding radial line profiles of a section of a GUV stained with dyad **1** showing a 2.7-fold resolution enhancement; RESOLFT parameters: 40 nm pixels; *λ*_ex_ 594 nm, 2.0 μW, 30 μs; *λ*_switch-off_ 405 nm, 335 μW, 50 μs; *λ*_recovery_ 640 nm, 20 μW (continuous wave), 30 ms; (e) confocal and RESOLFT images, and (f) corresponding radial line profiles of a section of a GUV stained with dyad **3** showing a 2.0-fold resolution enhancement; parameters: 40 nm pixels; *λ*_ex_ 594 nm, 2.0 μW, 30 μs; *λ*_switch-off_ 405 nm, 155 μW, 50 μs; *λ*_recovery_ 640 nm, 3.6 μW (pulsed), 30 ms. Confocal line profiles are fitted with Gaussian functions and RESOLFT profiles by Lorentzians (see ESI Section 7 for more details†).

We tested dyads **1–5** in giant unilamellar vesicles (GUVs) consisting of 1,2-dioleoyl-*sn-glycero*-3-phosphocholine (DOPC; lipid bilayer thickness 4–5 nm). The vesicles were formed by electroformation and then incubated with the dyads. GUVs provide a simple model membrane in which to investigate imaging with the dyads. Images were taken through the equatorial plane of the spherical GUVs, giving ring-shaped patterns (Fig. 4a). The choice of vesicle is important: the greater curvature of small vesicles produces a high background signal on the inside of the membrane, while imaging large vesicles requires focusing deep into the sample to reach the equatorial plane, which is not favorable for high resolution imaging. Therefore, a compromise must be made, and we imaged vesicles with diameters of 5–15 μm. In order to acquire RESOLFT images, we needed to develop a suitable laser pulse sequence, which was applied in a pixel-by-pixel fashion. We excited the dye using a Gaussian-shaped 594 nm spot to acquire a confocal image which was used as an internal control for each RESOLFT image. Then, the dyads were switched to their non-fluorescent form using a donut-shaped 405 nm irradiation. The Gaussian-shaped 594 nm spot was used to acquire the RESOLFT image from material at the center of the donut, which remains in the fluorescent form. Finally, recovery of the switch to its closed form was accelerated using 640 nm light (for details, see ESI Section 7†). Initial experiments established the laser powers required for efficient excitation and switching using confocal imaging (Fig. S18–S21†). These results allowed us to select appropriate parameters for RESOLFT imaging (Fig. 4 and S24–S28†). The thickness (full-width-at-half-maximum, FWHM) of the image of the membrane should correspond to the size of the point-spread function, *i.e.* resolution. The confocal line profiles are well described by Gaussian functions, while the RESOLFT line profiles are better fitted by Lorentzian functions.^23,25^ The RESOLFT line profiles can also be described by two coaxial Gaussian functions, where the width of one of the functions is fixed to match the confocal line profile.^14^ Such a model accounts for confocal contribution to the RESOLFT images, caused by incomplete switching off of the dyads (full details of the fitting can be found in ESI Section 7, Table S5†). FWHM values varied between different GUVs, even during confocal imaging, and the FWHM was often larger than the expected diffraction-limited value (212 nm) due to mechanical fluctuations in the membranes,^48^ as highlighted in previous STED microscopy images.^49^ Nevertheless, RESOLFT imaging gave average improvements in FWHM by around a factor of 2 compared with confocal images acquired simultaneously on the same membrane, with the best examples showing improvement factors of 2.7 (see Fig. S29† for analysis for all dyads).^50^ The average (and standard deviation) resolution enhancement factors from RESOLFT images of many GUVs were: 2.21 (0.36) for dyad **1**, 2.21 (0.25) for dyad **2**, 1.87 (0.32) for dyad **3**, 1.87 (0.28) for dyad **4** and 1.90 (0.15) for dyad **5**. Previous work imaging giant plasma membrane vesicles using well-established STED microscopy achieved only slightly better FWHM enhancement factors of around 3.^49^ In this case, the resolution enhancement is also limited by nanoscopic fluctuations in the membrane.

**Fig. 5:**
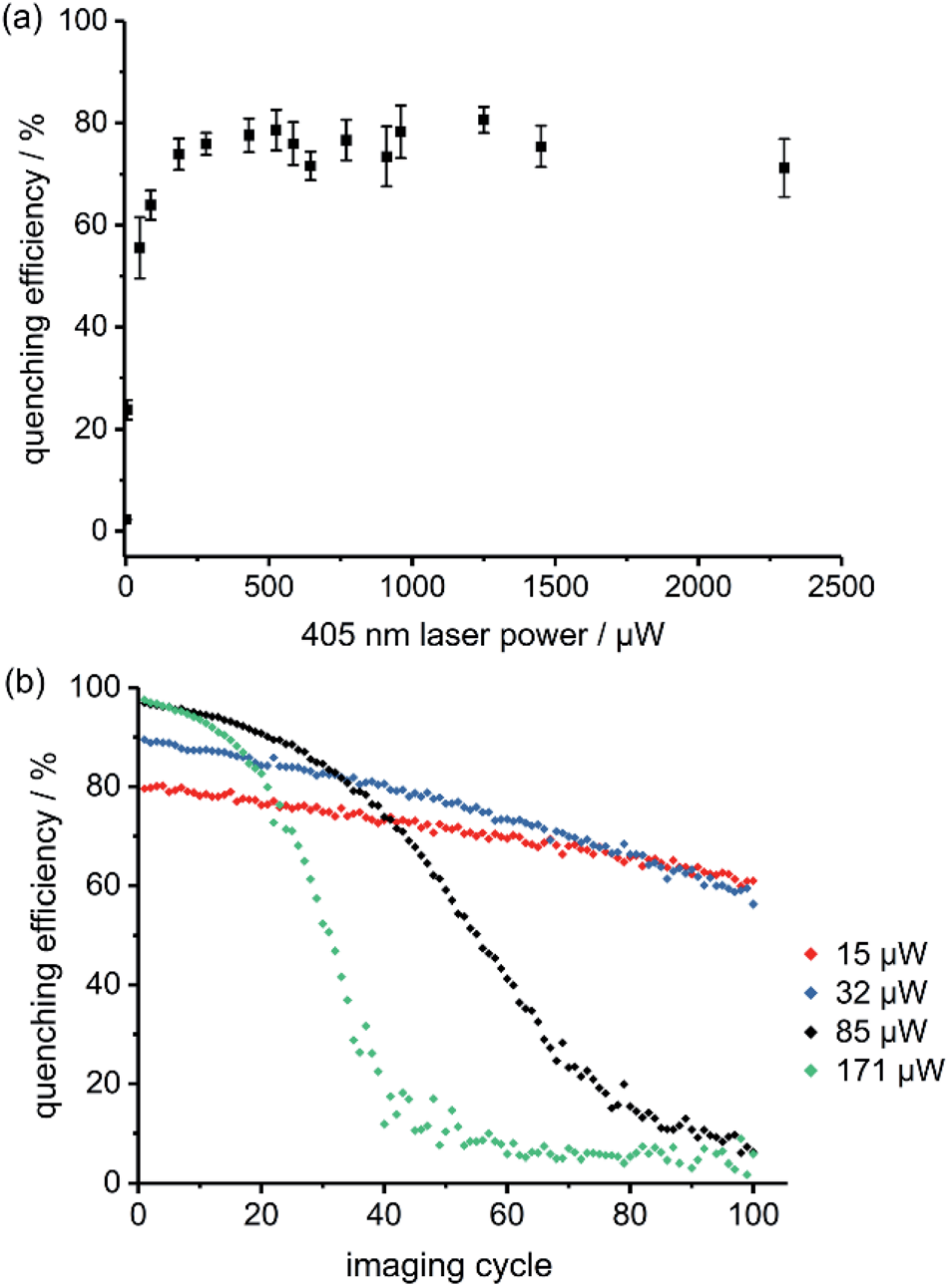
(a) Fluorescence quenching of dyad **1** in GUVs as a function of 405 nm switch-off laser power; pixel by pixel, *λ*_ex_ 594 nm, 2.0 μW, *λ*_recovery_ 640 nm, 20 μW (continuous wave), pixel size 40 nm, 5 repeats. (b) Fatigue of dyad **1** over 100 imaging cycles in GUVs at various 405 nm laser powers (line-by-line scanning, *λ*_ex_ 594 nm, 0.66 μW power, pixel dwell time 1.58 μs, pixel size 80 nm).

The best RESOLFT results for each of the five dyads required slightly different imaging parameters, which is perhaps surprising, given that the switching behavior is mostly a property of the spironaphthoxazine switch. Dyads **1** and **2** (bearing Cy3.5) performed better at higher 405 nm laser power than dyads **3–5** (bearing Atto590), as reflected by the power-dependent quenching curves in Fig. 5a and S18–S21.† Despite efforts to optimize the resolution, we were unable to surpass a mean FWHM enhancement factor of 2.2 with dyads **1–5**. This limit appeared to be a consequence of photochemical fatigue, so we set out to quantify the rates of fatigue of the dyads in GUVs, using confocal microscopy. There is no previously established method for assessing the fatigue of photoswitchable dyes under imaging conditions (*i.e.* in the microscope). We have quantified the viability of the dyad by the ‘quenching efficiency’ defined by eqn (1)

1 Quenching efficiency = (*I*_bright_ − *I*_dark_)/*I*_bright_

where *I*_bright_ and *I*_dark_ are the signal intensities of the fluorescence images before and after irradiation at 405 nm. We recorded the quenching efficiency over 100 imaging cycles on the same vesicle, at a range of 405 nm laser powers (total time per cycle ≈10 s). At higher 405 nm laser powers, the initial quenching efficiency was higher, but the switch underwent faster fatigue. Over the course of 100 imaging cycles, the bright state remained bright (indicating minimal photobleaching of the fluorescent dye), but that the dark state got brighter; the switch degraded so that it was no longer able to quench the fluorescent dye efficiently (see Fig. 5b and S32–S35† for dyad **1** and Fig. S36–S40† for dyads **2–5** and **6**). The fatigue curves for the dyads are strikingly sigmoidal, which is not expected for simple first-order chemical degradation.^51^

There are several possible explanations for the sigmoidal shape of these fatigue curves. If the dyad molecules are sufficiently close together, the spironaphthoxazine of one molecule can quench the fluorescent dye of a neighboring dyad. FRET is efficient over long distances (Förster radius: 4–6 nm), because of the good spectral overlap between the fluorescent dye and the active spironaphthoxazine quencher. Experiments using a 1 : 1 mixture of a ‘dummy dyad’ **6** (bearing no photoswitch) and dyad **3** resulted in initial quenching efficiencies of over 80% (Fig. S41†), which is only marginally lower than for pure dyad **3**. In the absence of intermolecular energy transfer, we would expect less than 50% quenching efficiency, because only half of the dyes are connected to switches. This confirms that intermolecular quenching occurs in the stained GUV membranes and may explain why some dyads perform better at different quenching laser powers. The calculated Förster radii (ESI Section 4†) for the dyads **3–5** (bearing Atto590) are higher than for dyads **1** and **2** (∼6 nm *vs.* ∼4 nm). If the Atto590 dyads quench each other over longer distances, then lower quenching laser powers will be sufficient to achieve the same quenching efficiency. We developed a kinetic model for the intermolecular quenching in an ensemble of dyads. In the simplest case, we consider a collection of *n* proximate dyads, such that one quencher can quench all the dyes in the ensemble. We can then calculate fatigue curves for different values of *n* (Scheme S7 and Fig. S44–S45; see ESI† for details of the model). Higher values of *n* lead to longer initial periods of high quenching efficiency before the sigmoidal decline (Fig. S45†).

Another possible explanation for the sigmoidal fatigue curves is that diffusion of dyad molecules within the membrane replenishes the population in the imaging plane (we are imaging a slice through a spherical vesicle). We tested this hypothesis using small patches of supported lipid bilayers (*ca.* 10–20 μm diameter), in which all the dyad molecules in a membrane patch were irradiated in each cycle, leaving no pool for replenishment between imaging cycles. Lateral diffusion of already-imaged molecules during the collection of a single image is still possible, as in GUVs, but there is no out-of-plane diffusion of fresh molecules. Fatigue curves in supported lipid bilayers were still sigmoidal, but the initial period before the decay became shorter than for GUVs (Fig. S42 and S43†). These results confirm that replenishment occurs by diffusion, but that it is not the only process which contributes to the sigmoidal shape. To gain further insight into the rate of diffusion of the dyads in the lipid bilayers, we carried out fluorescence correlation spectroscopy (FCS) experiments in GUVs (see ESI Section 9 for details†). FCS provides information on the transit time of a fluorescent molecule through the small observation spot. We recorded FCS curves for all dyads and a commercially available fluorescently-labeled lipid, Atto565-DPPE (see ESI Fig. S46–S51†). All the dyads showed similar diffusion coefficients (*D* = 10.5–12.7 μm^2^ s^−1^, Fig. S52†) which are only marginally slower than the commercial lipid (*D* = 13.3 μm^2^ s^−1^). These diffusion coefficients indicate that the dyad molecules have transit times through the confocal spot of around 1.4 ms. This confirms that in our RESOLFT imaging sequence, we quench and image (within 80 μs) the same molecules but that diffusion replenishes the dyads on the timescale of the recovery phase, *i.e.* when moving from pixel to pixel (approx. 30 ms).

## Conclusions

In summary, we have demonstrated that dyads consisting of a fluorescent dye covalently-linked to a quencher can be used for RESOLFT microscopy. An appealing feature of our dyads is that they can be switched in both directions using visible light (at 405 nm and 640 nm). RESOLFT microscopy of synthetic vesicles was achieved with an average resolution of around twice that of conventional confocal microscopy. The resolution is limited by mechanical motion in the vesicle membrane, as well as fatigue of the switch in the imaging environment. We suspect that similar dyads derived from other switching units may give even higher resolution. The fatigue curves are surprisingly sigmoidal, indicating that the dyad is replenished from outside the imaging plane between imaging cycles, and that quenching occurs intermolecularly between proximate dyads, as well as intramolecularly. The modular design, combined with the broad absorption spectrum of the open merocyanine, provides the opportunity to use dyes in different spectral regions for multicolor SRM. These dyads localize well in lipid bilayers and are promising agents for SRM of plasma membranes in simple cells, such as bacteria. The next challenge will be to create dyads functionalized with targeting groups for selective labeling of organelles in eukaryotic cells. The development of new switching units which allow switching wavelengths longer than 405 nm is also highly desirable.

## Conflicts of interest

There are no conflicts to declare.

## Supplementary Material

SC-011-D0SC02447C-s001
