# Supplementary material for: Super-resolution RESOLFT microscopy of lipid bilayers using a fluorophore-switch dyad
Source: Chem Sci. 2020 Aug 10;11(33):8955–60. doi: 10.1039/d0sc02447c (PMC8163400; doi:10.1039/d0sc02447c)
Supplement: SC-011-D0SC02447C-s001 [file SC-011-D0SC02447C-s001.pdf]

Electronic Supplementary Material (ESI) for Chemical Science.
This journal is © The Royal Society of Chemistry 2020

Electronic Supporting Information

Super-resolution RESOLFT microscopy of lipid bilayers using a fluorophore-switch dyad

Andrew T. Frawley,‡[a] Virginia Wycisk,‡[a] Yaoyao Xiong,[a] Silvia Galiani,[b] Erdinc Sezgin,[b] Iztok Urbančič,[b] Andreas Vargas Jentzsch,[a,c] Kathryn G. Leslie,[a] Christian Eggeling*[b,d,e,f] and Harry L. Anderson*[a]

[a] Department of Chemistry, University of Oxford, Chemistry Research Laboratory, Oxford, OX1 3TA, UK. E-mail: harry.anderson@chem.ox.ac.uk
[b] MRC Human Immunology Unit, Weatherall Institute of Molecular Medicine, University of Oxford, Oxford, OX3 9DS, UK. Email: christian.eggeling@rdm.ox.ac.uk
[c]. SAMS Research Group, Institut Charles Sadron, CNRS–UPR 22, University of Strasbourg, 67034, Strasbourg Cedex 2, France.
[d] Institute of Applied Optics and Biophysics, Friedrich-Schiller-University Jena, Max-Wien Platz 4, 07743 Jena, Germany.
[e] Leibniz Institute of Photonic Technology e.V., Albert-Einstein-Straße 9, 07745 Jena, Germany.
[f] Jena Center for Soft Matter (JCSM), Philosophenweg 7, 07743 Jena, Germany

‡ These authors contributed equally.

Table of Contents

1. General Methods S2
2. Synthetic Procedures S2
3. Photophysical Properties S14
4. Calculation of Förster Distances S20
5. Photoinduced Electron Transfer as a Potential Quenching Mechanism S21
6. Vesicle Preparation S24
7. Microscopy S25
8. Modeling Sigmoidal Fatigue S42
9. Fluorescence Correlation Spectroscopy S44
10. HPLC Analysis S47
11. NMR Spectra S51
12. References S64
13. Author Contributions S64

1. General Methods

Materials & methods

All reagents were purchased from commercial sources and used as received. Cy3.5 NHS ester was purchased from Lumiprobe GmbH. Normal phase flash column chromatography was carried out using SiO_2 (60 Å pore size, 40–63 µm particle size, Aldrich, UK) as the stationary phase.

NMR spectra were acquired on a Bruker AVII400, AVIII400, or AVII500 instrument at 298 K. NMR chemical shifts are reported in ppm relative to $SiMe_4$ (δ = 0) and were referenced internally with respect to residual solvent protons. Coupling constants are reported in Hz.

Electrospray mass spectrometry was carried out on a Waters Micromass LCT Premier XE spectrometer using 90:10 $MeOH:H_2O$ (+0.1% formic acid) as the mobile phase. High-resolution mass spectra (HRMS) were obtained on a Bruker µTOF instrument or a Waters GCT.

Optical analysis

All spectroscopic measurements were conducted in HPLC grade solvents using quartz cuvettes (10 mm path length, Starna Scientific Ltd, UK). UV-vis absorption spectra were acquired on a Perkin Elmer Lambda 20 spectrometer. Unless otherwise stated, all absorption spectra were recorded at 298 K, with temperature control by a PTP-1 Peltier unit from Perkin Elmer. Fluorescence spectra were acquired at 298 K using an Edinburgh Instruments FS5 spectrofluorometer operating Fluoracle® software, and equipped with a xenon arc lamp (providing 230–1000 nm excitation range), a thermostatic sample holder (SC-20) and both an R13456 PMT detector (200–950 nm spectral coverage, Hamamatsu) and an InGaAs analogue NIR detector (850–1650 nm spectral coverage). Quantum yields were measured by an absolute method using an integrating sphere (SC-30). Lifetimes were measured in time-correlated single photon counting (TCSPC) mode using a picosecond pulsed diode laser (EPL-475) as the excitation source.

2. Synthetic Procedures

Cy3.5 synthesis

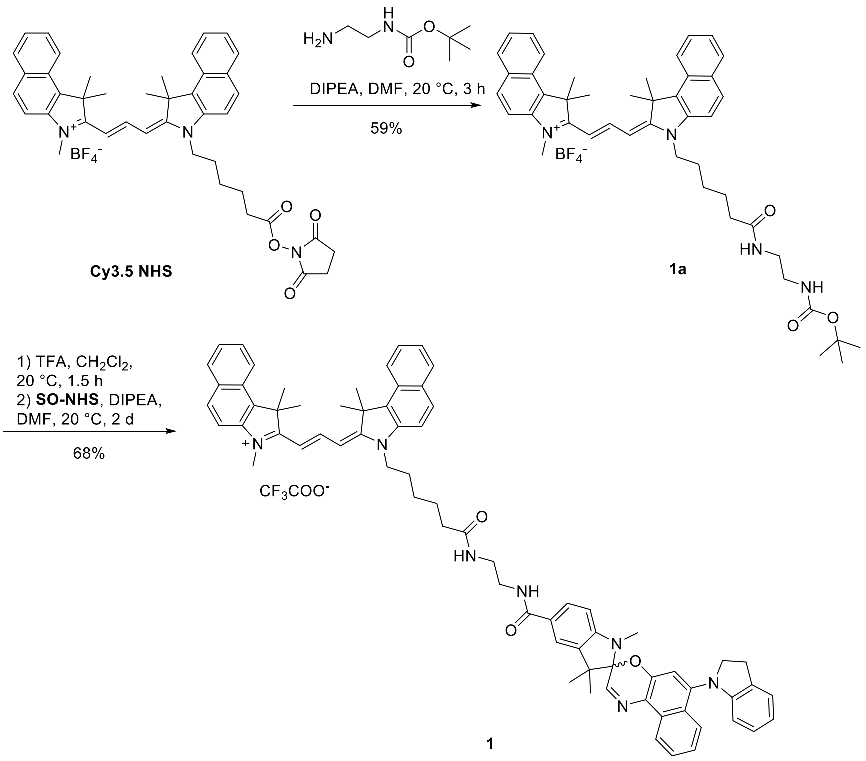

Scheme S1 Synthetic route to dyad **1**.

Cy3.5 Boc-ethylene diamine (1a)

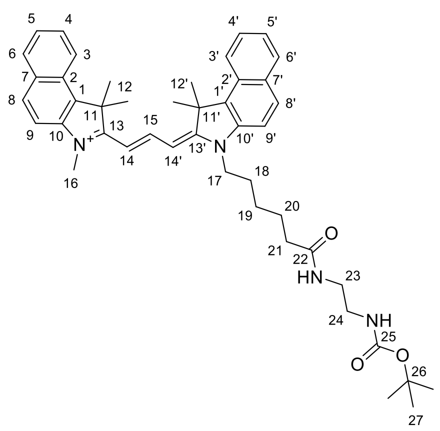

Tert-butyl (2-aminoethyl)carbamate (7.1 mg, 7.0 µL, 44 µmol) and DIPEA (9.6 mg, 13 µL, 74 µmol) were added from stock solutions in DMF (0.1 mM and 0.2 mM, respectively) to a solution of **Cy3.5 NHS** (22.0 mg, 30 µmol) in dry DMF (1.7 mL). The resulting deep purple solution was stirred at 20 °C for 3 h. The solvent was removed under reduced pressure, and the residue was treated with dichloromethane and washed with water (3 x 40 mL). The organic layer was dried over $MgSO_4$, filtered and concentrated. Purification by column chromatography (silica gel, 100% CH_2Cl_2 to 95:5 CH_2Cl_2/CH_3OH) yielded dye **1a** (14.0 mg, 59%) as a deep purple solid. R_f = 0.45 (CH_2Cl_2/MeOH 9:1); ^{1}H NMR (500 MHz, CD_3OD) δ 8.78 (t, $^3J_{H,H}$ 13.5, 1H, H^{15}), 8.30 (d, $^3J_{H,H}$ 8.6, 2H, H^3 + $H^{3'}$), 8.06 (dd, $^3J_{H,H}$ 8.9, 2.6, 2H, H^8 + $H^{8'}$), 8.02 (d, $^3J_{H,H}$ 8.2, 2H, H^6 + $H^{6'}$), 7.72–7.62 (m, 4H, H^4 + $H^{4'}$ + H^9 + $H^{9'}$), 7.52 (t, $^3J_{H,H}$ 7.5, 2H, H^5 + $H^{5'}$), 6.50 (d, $^3J_{H,H}$ 13.5, 1H, H^{14} or $H^{14'}$), 6.49 (d, $^3J_{H,H}$ 13.5, 1H, H^{14} or $H^{14'}$), 4.29 (t, $^3J_{H,H}$ 7.5, 2H, H^{17}), 3.82 (s, 3H, H^{16}), 3.19 (t, $^3J_{H,H}$ 6.3, 2H, H^{23}), 3.09 (t, $^3J_{H,H}$ 6.3, 2H, H^{24}), 2.23 (t, $^3J_{H,H}$ 7.3, 2H, H^{21}), 2.10 (s, 6H, H^{12} or $H^{12'}$), 2.10 (s, 6H, H^{12} or $H^{12'}$), 1.93 (pent., $^3J_{H,H}$ 7.5, 2H, H^{18}), 1.73 (pent., $^3J_{H,H}$ 7.3, 7.4, 2H, H^{20}), 1.54 (m, 2H, H^{19}), 1.38 (s, 9H, H^{27}); ^{13}C NMR (126 MHz, CD_3OD) δ 178.0 (C^{13}), 177.3 ($C^{13'}$), 176.1 (C^{22}), 158.5 (C^{25}), 150.8 (C^{15}), 141.5 (C^{10}), 140.8 ($C^{10'}$), 134.9 (C^1 or $C^{1'}$), 134.8 (C^1 or $C^{1'}$), 133.7 (C^7 or $C^{7'}$), 133.6 (C^7 or $C^{7'}$), 132.0 (C^8 or $C^{8'}$), 131.9 (C^8 or $C^{8'}$), 131.2 (C^6 + $C^{6'}$), 129.3 (C^2 or $C^{2'}$), 129.3 (C^2 or $C^{2'}$), 128.9 (C^4 + $C^{4'}$), 126.3 (C^5 + $C^{5'}$), 123.4 (C^3 + $C^{3'}$), 112.2 (C^9 or $C^{9'}$), 112.1 (C^9 or $C^{9'}$), 103.4 (C^{14} or $C^{14'}$), 103.1 (C^{14} or $C^{14'}$), 80.1 (C^{26}), 52.4 (C^{11} +$C^{11'}$), 45.2 (C^{17}), 40.9 (C^{24}), 40.5 (C^{23}), 36.7 (C^{21}), 32.1 (C^{16}), 28.7 (C^{27}), 28.5 (C^{18}), 28.0 (C^{12} or $C^{12'}$), 27.9 (C^{12} or $C^{12'}$), 27.3 (C^{19}), 26.5 (C^{20}); UV/Vis (MeOH): λ_{max} (ε) = 587 nm (77,000 mol^{-1} dm^3 cm^{-1}); fluorescence (MeOH): λ_{ex} = 500 nm; λ_{em} = 604 nm; Φ_{fl} = 7%; HRMS (ESI$^+$) *m/z* calcd for $C_{45}H_{55}N_4O_3^+$: 699.4269 [M$^+$]; found: 699.4265.

Cy3.5 spironaphthoxazine dyad (1)

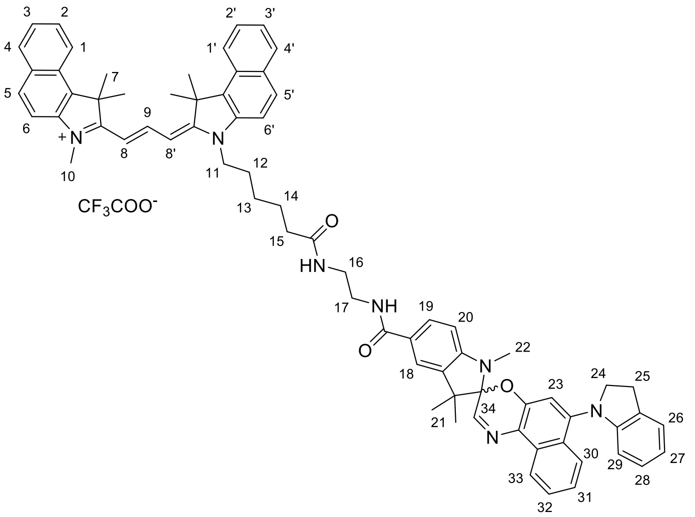

Dye **1a** (11.0 mg, 14 µmol) was dissolved in a 9:1 mixture of dichloromethane/TFA (1 mL) and the resulting purple solution was stirred at 20 °C for 1.5 h. The solvent was evaporated under reduced pressure and the residue was co-distilled with dichloromethane (4 x 3 mL). The purple solid (10.0 mg, 99%) and was used immediately without further purification. R_f = 0.07 (CH_2Cl_2/CH_3OH 9:1).

DIPEA (4.5 mg, 4.7 µL, 35 µmol) was added from a stock solution in DMF (35 µM) to deprotected dye **1a** (5.8 mg, 70 µmol) and **SO-NHS** (4.1 mg, 70 µmol). The mixture was diluted with DMF (0.5 mL) and stirred at 20 °C for 1 d. DIPEA (9.1 mg, 12.2 µL, 70 µmol) was added from a stock solution in DMF (22 µM) and the solution was continued to stir for 1 d. The crude mixture was concentrated and the residue was dissolved in dichloromethane (10 mL). The organic layer was washed with water (2 x 10 mL) and subsequently dried over $MgSO_4$, filtered and concentrated. The crude product was purified by column chromatography (silica gel, 100% CH_2Cl_2 to 95:5 CH_2Cl_2/CH_3OH) affording dyad **1** as a purple solid (6.9 mg, 68%). R_f = 0.35 (CH_2Cl_2/CH_3OH 9:1); ^{1}H NMR (400 MHz, 2:1 $CD_3OD/CDCl_3$) δ 8.72 (t, $^3J_{H,H}$ 13.5, 1H, H^9), 8.53 (dt, $^3J_{H,H}$ 8.6, 1.0, 1H, H^{33}), 8.24 (dd, $^3J_{H,H}$ 8.3, 4.8, 2H, H^1 + $H^{1'}$), 8.07–7.96 (m, 4H, H^4 + $H^{4'}$ + H^5 + $H^{5'}$), 7.88 (dt, $^3J_{H,H}$ 8.4, 1.0, 1H, H^{30}), 7.72–7.45 (m, 10H, H^2 + $H^{2'}$ + H^3 + $H^{3'}$ + H^6 + $H^{6'}$ + H^{18} + H^{19} + H^{32} + H^{34}), 7.34 (ddd, $^3J_{H,H}$ 8.3, 6.8, 1.3, 1H, H^{31}), 7.13 (dd, $^3J_{H,H}$ 7.4, 1.3, 1H, H^{26}), 6.88–6.81 (m, 1H, H^{28}), 6.81 (s, 1H, H^{23}), 6.67 (td, $^3J_{H,H}$ 7.4, 1.0, 1H, H^{27}), 6.54 (d, $^3J_{H,H}$ 8.2, 1H, H^{20}), 6.53 (d, $^3J_{H,H}$ 13.5, 1H, H^8 or $H^{8'}$) 6.45 (d, $^3J_{H,H}$ 13.5, 1H, H^8 or $H^{8'}$), 6.12 (d, $^3J_{H,H}$ 7.9, 1H, H^{29}), 4.14 (m, 2H, H^{11}), 3.82–3.74 (m, 5H, H^{10} + H^{24}), 3.52–3.44 (m, 2H, H^{16} or H^{17}), 3.42–3.35 (m, 2H, H^{16} or H^{17}), 3.06 (br. s, 3H, H^{25}), 2.76 (s, 3H, H^{22}), 2.23 (t, $^3J_{H,H}$ 7.1, 2H, H^{15}), 2.05 (s, 6H, H^7 or $H^{7'}$), 2.03 (s, 6H, H^7 or $H^{7'}$), 1.85 (pent., $^3J_{H,H}$ 7.2, 2H, H^{12}), 1.70 (m, 2H, H^{14}), 1.47 (m, 2H, H^{13}), 1.29 (s, 6H, H^{21}); UV/Vis (MeOH): λ_{max} (ε) = 589 nm (56,000 mol^{-1} dm^3 cm^{-1}); fluorescence (MeOH): λ_{ex} = 500 nm; λ_{em} = 604 nm; Φ_{fl} = 9%; HRMS (ESI$^+$) *m/z* calcd for $C_{71}H_{72}N_7O_3^+$: 1070.5691 [M$^+$]; found: 1070.5685.

Dyad 2 synthesis

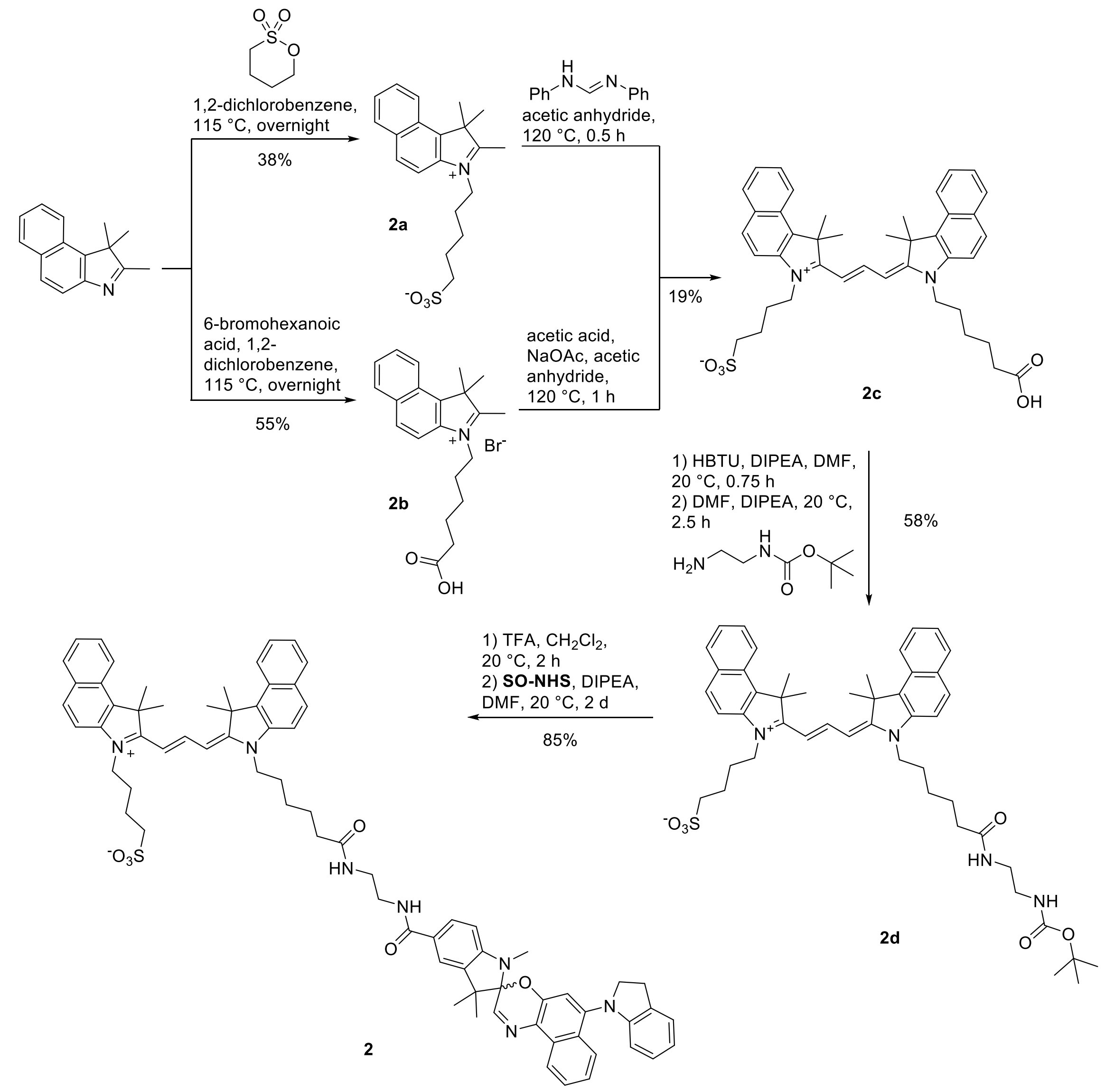

Scheme S2 Synthetic procedure to sulfonated dyad **2**.

4-(1,1,2-Trimethyl-1*H*-benzo[*e*]indol-3-ium-3-yl)butane-1-sulfonate (2a)

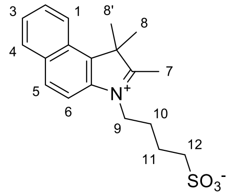

Indole **2a** was synthesized according to a modified procedure from literature.[1] 1,1,2-Trimethyl-1*H*-benzo[*e*]indole (1.0 g, 4.8 mmol) and 1,4-butanesultone (1.80 g, 13.4 mmol) were dissolved in 1,2-dichlorobenzene (20 mL) and stirred at 115 °C overnight. After cooling to 20 °C, the mixture was precipitated with Et_2O (500 mL) and the precipitated product was washed with Et_2O several times. The crude product **2a** was obtained as a pale blue solid (0.60 g, 38%). ^{1}H NMR (400 MHz, DMSO-d_6) δ 8.36 (dd, $^3J_{H,H}$ 8.4, 1.2, 1H, H^1), 8.27 (d, $^3J_{H,H}$ 8.9, 1H, H^5), 8.21 (dd, $^3J_{H,H}$ 8.2, 1.2, 1H, H^4), 8.0 (d, $^3J_{H,H}$ 8.9, 1H, H^6), 7.78 (ddd, $^3J_{H,H}$ 8.4, 6.9, 1.2, 1H, H^2), 7.72 (ddd, $^3J_{H,H}$ 8.2, 6.9, 1.2, 1H, H^3), 4.60 (t, $^3J_{H,H}$ 7.8, 2H, H^9), 2.94 (s, 2H, H^7), 2.53 (t, $^3J_{H,H}$ 7.1, 2H, H^{12}, overlapping with solvent), 2.06–1.99 (m, 2H, H^{11}), 1.84–1.75 (m, 2H, H^{10}), 1.75 (s, 6H, H^8 + $H^{8'}$); MS (ESI$^+$) *m/z* calcd for $C_{19}H_{23}NNaO_3S^+$: 368.1 [M+Na$^+$]; found: 368.2.

3-(5-Carboxypentyl)-1,1,2-trimethyl-1*H*-benzo[*e*]indol-3-ium bromide (2b)

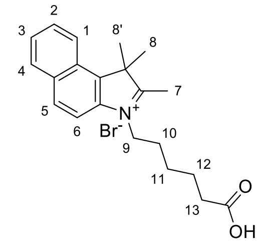

Indole **2b** was synthesized according to modified procedures from literature.[1,2] 1,1,2-Trimethyl-1*H*-benzo[*e*]indole (1.5 g, 7.2 mmol) and 6-bromohexanoic acid (3.90 g, 20.2 mmol) were dissolved in 1,2-dichlorobenzene (8 mL) and stirred at 110 °C overnight. After cooling to 20 °C, the mixture was precipitated with Et_2O (500 mL) and the precipitated product was washed with Et_2O and dichloromethane several times. The crude product **2b** was obtained as a brown solid (1.6 g, 55%). ^{1}H NMR (400 MHz, DMSO-d$_6$) δ 8.37 (dd, $^3J_{H,H}$ 8.4, 1.2, 1H, H^1), 8.29 (d, $^3J_{H,H}$ 8.9, 1H, H^5), 8.22 (dd, $^3J_{H,H}$ 8.0, 1.2, 1H, H^4), 8.15 (d, $^3J_{H,H}$ 8.9, 1H, H^6), 7.79 (ddd, $^3J_{H,H}$ 8.4, 6.9, 1.2, 1H, H^2 or H^3), 7.73 (ddd, $^3J_{H,H}$ 8.0, 6.9, 1.2, 1H, H^2 or H^3), 4.58 (t, $^3J_{H,H}$ 7.7 Hz, 2H, H^9), 2.94 (s, 2H, H^7), 2.23 (t, $^3J_{H,H}$ 7.2, 2H, H^{13}), 1.90 (pent., $^3J_{H,H}$ 7.7, 2H, H^{10}), 1.76 (s, 6H, H^8 + $H^{8'}$), 1.57 (pent., $^3J_{H,H}$ 7.2, 2H, H^{12}), 1.52–1.40 (m, 2H, H^{11}); MS (ESI$^+$) *m/z* calcd for $C_{21}H_{26}NO_2^+$: 324.1958 [M$^+$]; found: 324.2.

Sulfo-Cy3.5 carboxylic acid (2c)

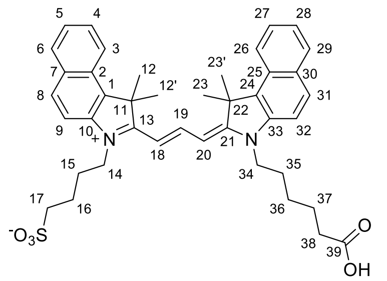

Dye **2c** was synthesized according to a modified procedure from literature.[3] 4-(1,1,2-Trimethyl-1*H*-benzo[*e*]indol-3-ium-3-yl)butane-1-sulfonate **2a** (263 mg, 0.76 mmol) and N,N-diphenylformamidine (150 mg, 0.76 mmol) were dissolved in acetic anhydride (11 mL) and stirred at 120 °C for 30 min. To the above solution were added 3-(5-carboxypentyl)-1,1,2-trimethyl-1*H*-benzo[*e*]indol-3-ium bromide **2b** (307 mg, 0.76 mmol), sodium acetate (187 mg, 2.28 mmol) and acetic anhydride (5 mL). Then acetic acid (0.55 mL) was added and the mixture was stirred at 120 °C for 1 h. The purple solution was cooled to 20 °C and the solvent was removed in vacuo. The residue was dissolved in methanol (10 mL) and precipitated with diethyl ether (250 mL). The precipitated product was dissolved in methanol (10 mL) and washed with diethyl ether (250 mL) several times. The crude product was purified by column chromatography (silica gel, 100% CH_2Cl_2 to 80:20 CH_2Cl_2/CH_3OH) and dye **2c** was obtained as a red solid (100 mg, 19%). R_f = 0.49 (CH_2Cl_2/ CH_3OH:1); ^{1}H NMR (500 MHz, CD_3OD) δ 8.73 (t, $^3J_{H,H}$ 13.5, 1H, H^{19}), 8.27 (d, $^3J_{H,H}$ 8.5, 2H, H^3 + H^{26}), 8.03 (dd, $^3J_{H,H}$ 8.8, 2.1, 2H, H^8 + H^{31}), 8.02–7.96 (m, 2H, H^6 + H^{29}), 7.69–7.62 (m, 3H, H^4 + H^{27} and H^9 or H^{32}), 7.61 (d, $^3J_{H,H}$ 8.8, 1H, H^9 or H^{32}), 7.53–7.46 (m, 2H, H^5 + H^{28}), 6.57 (d, $^3J_{H,H}$ 13.5, 1H, H^{18} or H^{20}), 6.53 (d, $^3J_{H,H}$ 13.5, 1H, H^{18} + H^{20}), 4.30 (t, $^3J_{H,H}$ 7.1, 2H, H^{14}), 4.27 (t, $^3J_{H,H}$ 7.9, H^{34}), 2.95 (t, $^3J_{H,H}$ 6.9, 2H, H^{17}), 2.30 (t, $^3J_{H,H}$ 6.8, 2H, H^{38}), 2.15–1.98 (m, 16H, H^{12} + $H^{12'}$ + H^{15} + H^{16} + H^{23} + $H^{23'}$), 1.91 (pent., $^3J_{H,H}$ 7.9, 2H, H^{35}), 1.78–1.68 (m, 2H, H^{37}), 1.62–1.52 (m, 2H, H^{36}); ^{13}C NMR (126 MHz, CD_3OD) δ 177.2 (C^{13} + C^{21}), 150.8 (C^{19}), 140.8 (C^{10} + C^{33}), 134.9 (C^1 or C^{24}), 134.8 (C^1 or C^{24}), 133.57 (C^7 or C^{30}), 133.55 (C^7 or C^{30}), 132.0 (C^8 or C^{31}), 131.9 (C^8 or C^{31}), 131.1 (C^6 + C^{29}), 129.3 (C^2 + C^{25}), 128.9 (C^4 + C^{27}), 126.3 (C^5 + C^{28}), 123.3 (C^3 + C^{26}), 112.3 (C^9 or C^{32}), 112.2 (C^9 or C^{32}), 103.3 (C^{18} + C^{20}), 52.3 (C^{11} + C^{22}), 51.7 (C^{17}), 45.3 (C^{14} or C^{34}), 45.2 (C^{14} or C^{34}), 36.4 (C^{38}), 28.6 (C^{35}), 28.0 (C^{12} + $C^{12'}$ + C^{23} + $C^{23'}$), 27.5 (C^{16} + C^{36}), 26.4 (C^{37}), 23.5 (C^{15}); UV/Vis (MeOH): λ_{max} (ε) = 590 nm (91,000 mol^{-1} dm^3 cm^{-1}); fluorescence (MeOH): λ_{ex} = 500 nm; λ_{em} = 606 nm; Φ_{fl} = 12%; HRMS (ESI$^+$) *m/z* calcd for $C_{41}H_{47}N_2O_5S^+$: 679.3200 [M+H$^+$]; found: 679.3196.

Sulfo-Cy3.5 Boc-ethylenediamine linker (2d)

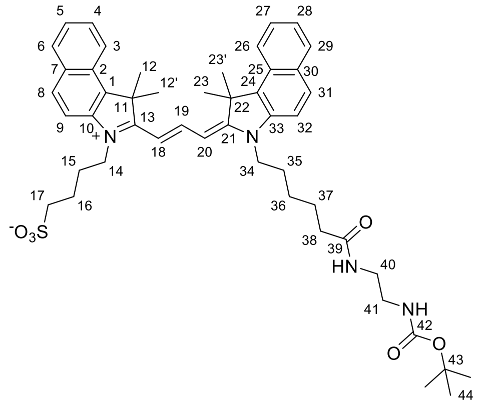

To a solution of dye **2c** (25 mg, 37 µmol) and HBTU (24 mg, 63 µmol) in DMF (1.5 mL) was added DIPEA (17 mg, 23 µL, 136 µmol) from a stock solution in DMF (1.6 mM). The resulting mixture was stirred at 20 °C under argon for 45 min followed by the addition of *tert*-butyl (2-aminoethyl)carbamate (9 mg, 56 µmol) and DIPEA (9 mg, 11 µL, 68 µmol) from stock solutions in DMF (196 mM and 1.6 mM, respectively). The resulting deep purple solution was stirred at 20 °C for 2.5 h and subsequently concentrated and dried. The residue was purified by column chromatography (silica gel, 100% CH_2Cl_2 to 80:20 CH_2Cl_2/CH_3OH) and yielded dye **2d** (18 mg, 58%) as a deep purple solid. R_f = 0.69 (CH_2Cl_2/MeOH 4:1); ^{1}H NMR (500 MHz, CD_3OD) δ 8.74 (t, $^3J_{H,H}$ 13.5, 1H, H^{19}), 8.28 (d, $^3J_{H,H}$ 8.6, 2H, H^3 + H^{26}), 8.04 (d, $^3J_{H,H}$ 8.9, 2H, H^8, H^{31}), 8.01 (d, $^3J_{H,H}$ 8.4, 2H, H^6 + H^{29}), 7.70–7.60 (m, 4H, H^4 + H^9 + H^{27} + H^{32}), 7.54–7.47 (m, 2H, H^5 + H^{28}), 6.63 (d, $^3J_{H,H}$ 13.5, 1H, H^{18} or H^{20}), 6.56 (d, $^3J_{H,H}$ 13.5, 1H, H^{18} or H^{20}), 4.32 (t, $^3J_{H,H}$ 6.6, 2H, H^{14}), 4.28 (t, $^3J_{H,H}$ 7.6, 2H, H^{34}), 3.19 (t, $^3J_{H,H}$ 6.3, 2H, H^{40}), 3.09 ($^3J_{H,H}$ 6.3, 2H, H^{41}), 2.97 (t, $^3J_{H,H}$ 6.9, 2H, H^{17}), 2.26 (t, $^3J_{H,H}$ 7.3, 2H, H^{38}), 2.17–2.00 (m, 16H, H^{12} + $H^{12'}$ + H^{15} + H^{16} +

H^{23} + $H^{23'}$), 1.92 (pent., $^3J_{H,H}$ 7.6, 2H, H^{35}), 1.73 (pent., $^3J_{H,H}$ 7.3, 2H, H^{37}), 1.60–1.50 (m, 2H, H^{36}), 1.35 (s, 9H, H^{44}); ^{13}C NMR (126 MHz, CD_3OD) δ 177.3 (C^{21}), 177.2 (C^{13}), 176.2 (C^{39}), 158.4 (C^{42}), 150.7 (C^{19}), 140.84 (C^{33}), 140.82 (C^{10}), 134.9 (C^1 + C^{24}), 133.6 (C^7 + C^{30}), 132.0 (C^8 or C^{31}), 131.9 (C^8 or C^{31}), 131.2 (C^6 + C^{29}), 129.3 (C^2 + C^{25}), 128.9 (C^4 + C^{27}), 126.3 (C^5 + C^{28}), 123.3 (C^3 + C^{26}), 112.3 (C^9 + C^{32}), 103.6 (C^{18} + C^{20}), 80.0 (C^{43}), 52.4 (C^{11} + C^{22}), 51.5 (C^{17}), 45.3 (C^{14} or C^{34}), 45.2 (C^{14} or C^{34}), 41.0 (C^{41}), 40.5 (C^{40}), 36.8 (C^{38}), 28.7 (C^{44}), 28.6 (C^{35}), 28.1 (C^{12} + $C^{12'}$ or C^{23} + $C^{23'}$), 28.0 (C^{12} + $C^{12'}$ or C^{23} + $C^{23'}$), 27.33 (C^{36}), 27.29 (C^{16}), 26.6 (C^{37}), 23.5 (C^{15}); UV/Vis (CH_3OH): λ_{max} (ε) = 590 nm (101,000 mol^{-1} dm^3 cm^{-1}); fluorescence (CH_3OH): λ_{ex} = 500 nm; λ_{em} = 607 nm; Φ_{fl} = 15%; HRMS (ESI$^+$) *m/z* calcd for $C_{48}H_{60}N_4O_6S+H^+$: 821.4306 [M^+]; found: 821.4298.

Sulfo-Cy3.5 spironaphthoxazine dyad (2)

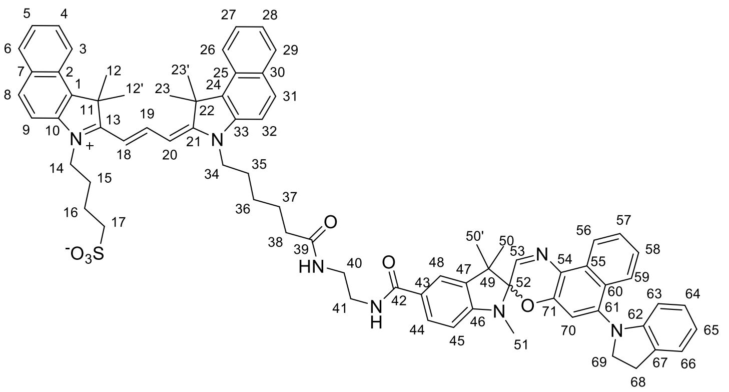

Dye **2d** (11 mg, 13 µmol) was dissolved in CH_2Cl_2 (0.9 mL) and trifluoroacetic acid (0.1 mL) was added. The resulting purple solution was stirred at 20 °C for 2 h. The mixture was concentrated and the residue was co-distilled with CH_2Cl_2 5 times. The deprotected dye was yielded as a purple solid (9.0 mg, 97%) and was used immediately without further purification. R_f = 0.39 (CH_2Cl_2/MeOH 4:1).

To a solution of the deprotected dye (11 mg, 13 µmol) and **SO-NHS** (6.5 mg, 11 µmol) in DMF (1.5 mL) was added DIPEA (7.1 mg, 9 µL, 55 µmol) from a stock solution in DMF (3.4 M) and the mixture was stirred at 20 °C overnight. The next day DIPEA (7.1 mg, 9 µL, 55 µmol) was added from the same stock solution (3.4 M) and the mixture was stirred for 6 h. In order to drive the reaction to completion, **SO-NHS** (8 mg, 13 µmol) was added and the solution was stirred at 20 °C overnight. The crude mixture was concentrated and the residue was dissolved in CH_2Cl_2. The organic layer was washed with water (3 x 5 mL) and the combined organic layers were concentrated. The crude product was purified by column chromatography (silica gel, 100% CH_2Cl_2 to 85:15 CH_2Cl_2/CH_3OH) affording dyad **2** as a purple solid (11 mg, 85%). R_f = 0.32 (CH_2Cl_2/MeOH 4:1); ^{1}H NMR (400 MHz, CD_3OD) δ 8.68 (dd, $^3J_{H,H}$ 13.3, 13.5, 1H, H^{19}), 8.58–8.50 (m, 1H, H^{56}), 8.21 (dd, $^3J_{H,H}$ 8.6, 3.4, 2H, H^3 + H^{26}), 8.07–7.94 (m, 4H, H^6 + H^8 + H^{29} + H^{31}), 7.85 (d, $^3J_{H,H}$ 8.3, 1H, H^{59}), 7.69 (dd, $^3J_{H,H}$ 8.2, 1.8, 1H, H^{44}), 7.67–7.62 (m, 3H, H^4 + H^{27} and H^9 or H^{32}), 7.62 (s, 1H, H^{53}), 7.60 (d, $^3J_{H,H}$ 1.8, 1H, H^{48}), 7.58–7.53 (m, 2H, H^{57} and H^9 or H^{32}), 7.52–7.46 (m, 2H, H^5 + H^{28}), 7.33 (ddd, $^3J_{H,H}$ 8.3, 6.8, 1.3, 1H, H^{58}), 7.17–7.04 (m, 1H, H^{66}), 6.81 (td, $^3J_{H,H}$ 7.7, 7.6, 1H, H^{64}), 6.77 (s, 1H, H^{70}), 6.69 (d, $^3J_{H,H}$ 13.3, 1H, H^{18}), 6.68–6.63 (m, 1H, H^{65}), 6.53 (d, $^3J_{H,H}$ 8.2, 1H, H^{45}), 6.52 (d, $^3J_{H,H}$ 13.5, 1H, H^{20}), 6.10 (d, $^3J_{H,H}$ 7.7, 1H, H^{63}), 4.28 (t, $^3J_{H,H}$ 7.6, 2H, H^{14}), 4.21–4.02 (m, 2H, H^{34}), 3.72–3.62 (m, 2H, H^{69}), 3.54–3.44 (m, 2H, H^{41}), 3.43–3.37 (m, 2H, H^{40}), 2.98 (br. s, 2H, H^{68}), 2.93 (t, $^3J_{H,H}$ 6.7, 2H, H^{17}), 2.74 (s, 3H, H^{51}), 2.28 (t, $^3J_{H,H}$ 7.1, 2H, H^{38}), 2.12–1.93 (m, 16H, H^{15} + H^{16} + H^{12} + $H^{12'}$ + H^{23} + $H^{23'}$), 1.84 (pent., $^3J_{H,H}$ = 7.7, 1H, H^{35}), 1.71 (pent., $^3J_{H,H}$ = 7.1, 1H, H^{37}), 1.56–1.44 (m, 2H, H^{36}), 1.36–1.21 (m, 6H, H^{50} + $H^{50'}$); ^{13}C NMR (126 MHz, CD_3OD) δ 177.2 (C^{13}), 177.1 (C^{21}), 176.6 (C^{39}), 170.1 (C^{42}), 151.9 (C^{46}), 151.1 (C^{62}), 150.6 (C^{19}), 150.5 (C^{53}), 145.9 (C^{61}), 145.5 (C^{53}), 140.8 (C^{10} or C^{33}), 140.7 (C^{10} or C^{33}), 137.3 (C^{47}), 134.9 (C^{24}), 134.8 (C^1), 133.6 (C^7 + C^{30}), 133.0 (C^{55}), 132.2 (C^{67}), 132.0 (C^8 + C^{31}), 131.2 (C^6 + C^{29}), 129.8 (C^{44}), 129.3 (C^2 or C^{25}), 129.3 (C^2 or C^{25}), 128.9 (C^4 + C^{27}), 128.7 (C^{57}), 127.7 (C^{64}), 126.8 (C^{60}), 126.7 (C^{43}), 126.3 (C^5 + C^{28}), 125.8 (C^{66}), 125.7 (C^{59}), 124.8 (C^{58}), 123.3 (C^3 or C^{26}), 122.9 (C^3 or C^{26}), 122.1 (C^{54}), 121.9 (C^{48}), 120.3 (C^{65}), 112.3 (C^9 or C^{32}), 112.2 (C^9 or C^{32}), 110.5 (C^{63}), 109.5 (C^{70}), 107.4 (C^{45}), 103.7 (C^{18}), 103.5 (C^{20}), 100.2 (C^{52}), 56.1 (C^{69}), 52.6 (C^{49}), 52.31 (C^{22}), 52.28 (C^{11}), 51.5 (C^{17}), 45.2 (C^{34}), 45.1 (C^{14}), 40.8 (C^{41}), 40.1 (C^{40}), 36.9 (C^{38}), 29.7 (C^{51}), 29.7 (C^{68}), 28.7 (C^{35}), 28.1 (C^{12} or C^{23}), 28.0 (C^{12} or C^{23}), 27.3 (C^{36}), 27.2 (C^{16}), 26.7 (C^{37}), 25.8 (C^{50} or $C^{50'}$), 23.5 (C^{15}), 21.3 (C^{50} or $C^{50'}$); UV/Vis (MeOH): λ_{max} (ε) = 587 nm (97,000 mol^{-1} dm^3 cm^{-1}); fluorescence (MeOH): λ_{ex} = 500 nm; λ_{em} = 607 nm; Φ_{fl} = 11%; HRMS (ESI$^+$) *m/z* calcd for $C_{74}H_{77}N_7O_6S+H^+$: 1192.5729 [M^+]; found: 1192.5725.

Atto590 dyads

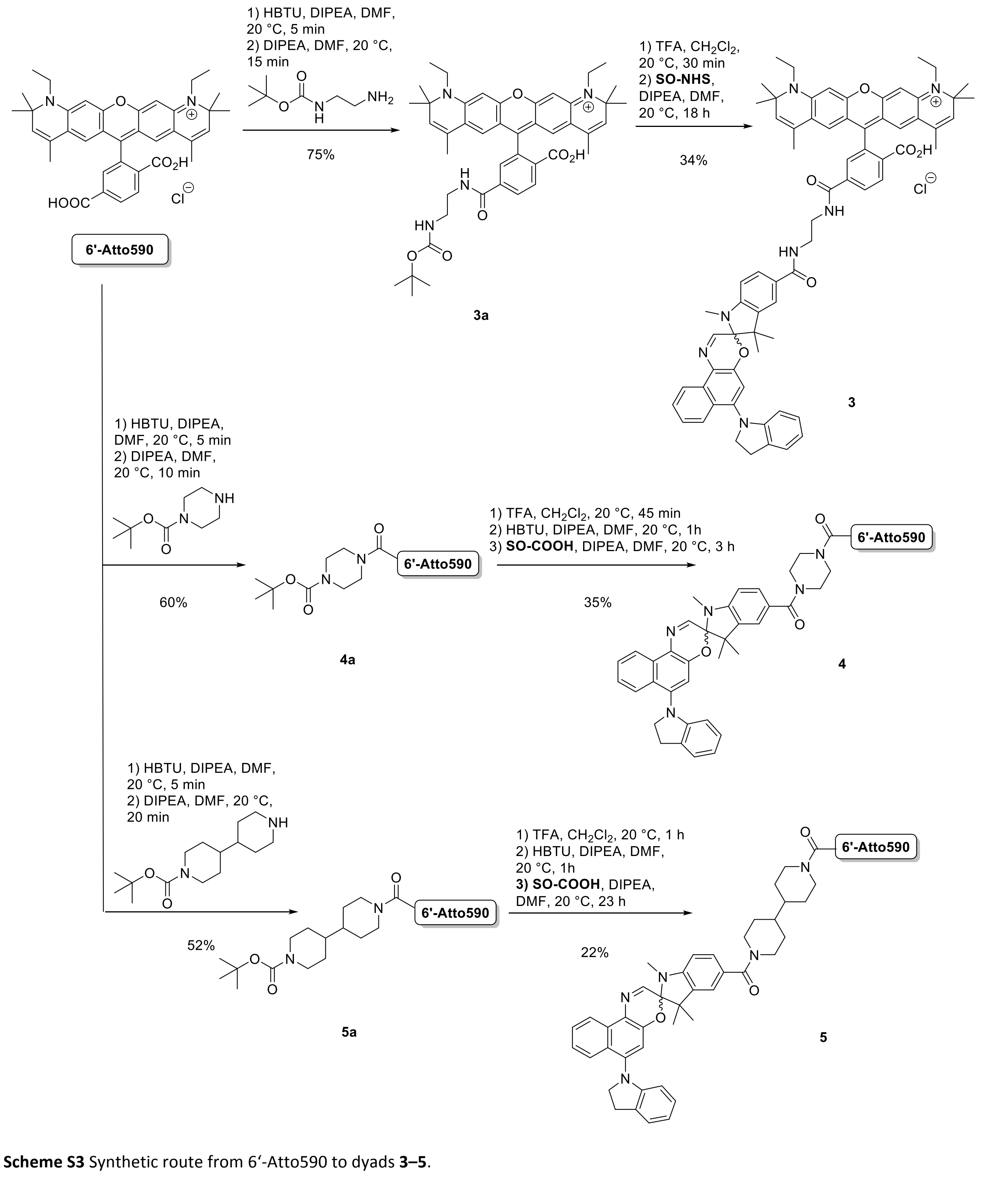

Scheme S3 Synthetic route from 6′-Atto590 to dyads **3–5**.

6’-Atto590-*N*-Boc-protected linker (3a)

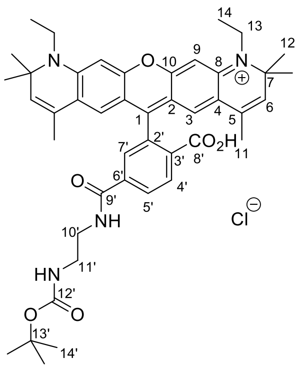

6’-Atto590 chloride (24.0 mg, 38 µmol) and HBTU (17.4 mg, 46 µmol) were dissolved in DMF (2.5 mL). To this solution was added DIPEA (60 µL, 340 µmol) and the reaction mixture was stirred for 5 min under argon at 20 °C. *Tert*-butyl (2-aminoethyl)carbamate (6.1 mg, 38 µmol) was added as a solution in DMF (0.3 mL). The reaction mixture was stirred at 20 °C under argon for 15 min, at which time it was quenched by the addition of water (3 mL). The solvent was removed under reduced pressure and the residue was dissolved in dichloromethane (20 mL), then washed with water (2 × 10 mL) followed by 2% aqueous HCl (1 × 10 mL). The organic layer was concentrated under reduced pressure and the crude product was purified by column chromatography (SiO_2, 97:3 to 85:15 CH_2Cl_2:CH_3OH) to yield dye **3a** as a blue solid (22 mg, 75%); R_f: 0.51 (SiO_2, 90:10 CH_2Cl_2:CH_3OH); ^1H NMR (500 MHz, CD_3OD) δ 8.18 (d, $^3J_{H\text{-}H}$ 7.6, 1H, $H^{5'}$), 8.11 (d, $^3J_{H\text{-}H}$ 7.6, 1H, $H^{4'}$), 7.72 (s, 1H, $H^{7'}$), 6.87 (s, 2H, H^9), 6.81 (s, 2H, H^3), 5.59 (s, 2H, H^6), 3.73 (q, $^3J_{H\text{-}H}$ 7.0, 4H, H^{13}), 3.46 (t, $^3J_{H\text{-}H}$ 6.0, 2H, $H^{10'}$), 3.27 (t, $^3J_{H\text{-}H}$ 6.0, 2H, $H^{11'}$), 1.76 (s, 6 H, H^{11}), 1.49 (s, 12 H, H^{12}), 1.37 (t, $^3J_{H\text{-}H}$ 7.0, 6 H, H^{14}), 1.31 (s, 9H, $H^{14'}$); ^{13}C NMR (126 MHz, CD_3OD) δ 172.3 ($C^{8'}$), 168.8 ($C^{9'}$), 159.6 (C^1), 159.5 (C^{10}), 158.9 ($C^{12'}$), 153.5 (C^8), 136.8 ($C^{6'}$), 133.8 ($C^{2'}$ or $C^{3'}$), 133.6 (C^6), 131.3 ($C^{5'}$), 129.7 ($C^{4'}$), 129.6 ($C^{7'}$), 126.8 (C^5), 124.7 (C^4), 123.9 (C^9), 115.2 (C^2), 96.7 (C^3), 80.0 ($C^{13'}$), 61.4 (C^7), 41.9 ($C^{10'}$), 41.1 (C^{13}), 40.6 ($C^{11'}$), 29.6 (C^{12}), 28.7 ($C^{14'}$), 18.3 (C^{11}), 13.6 (C^{14}); UV/Vis (MeOH): λ_{max} (ε) = 586 nm (123,000 mol^{-1} dm^3 cm^{-1}); fluorescence (MeOH): λ_{ex} = 500 nm; λ_{em} = 612 nm; Φ_{fl} = 87%; HRMS (ESI$^+$) *m/z* calcd for $C_{44}H_{53}N_4O_6^+$: 733.3960 [M$^+$]; found: 733.3958.

6’-Atto590-ethylenediamine-SO dyad (3)

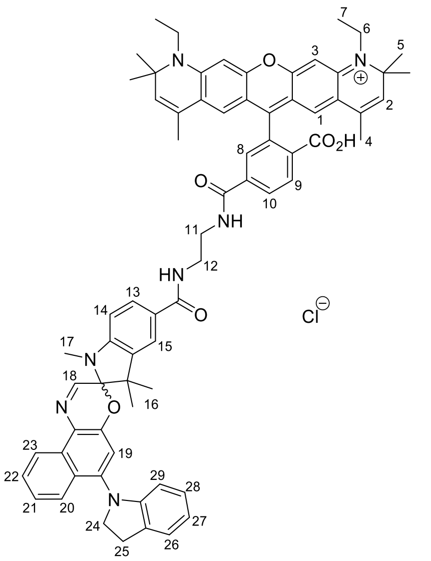

Dye **3a** (6.5 mg, 8.5 µmol) was dissolved in anhydrous dichloromethane (0.9 mL) and trifluoroacetic acid (0.1 mL) was added. The resulting purple solution was stirred at 20 °C under argon for 30 min, after which time the solvent was removed under high vacuum. Dichloromethane (1 mL) was added to the residue and the solvent removed under vacuum. This process was repeated 5 times. The resulting blue solid was used immediately without further purification assuming quantitative conversion; R_f: 0.06 (SiO_2, 90:10 CH_2Cl_2:CH_3OH).

Crude 6’-Atto590-ethylenediamine conjugate (7.3 mg, 8.5 µmol) was dissolved in DMF (0.5 mL). The **SO-NHS** (6.0 mg, 10 µmol) was added as a solution in DMF containing DIPEA (30 µL DIPEA in 1 mL). The resulting blue solution was stirred at 20 °C under argon for 18 h after which time the solvent was removed under reduced pressure. The residue was dissolved in dichloromethane (10 mL) and washed with water (10 mL). The aqueous solution was extracted with dichloromethane (3 × 10 mL), and the organic layers were combined, washed with brine (10 mL), and concentrated. The crude product was subjected to multiple column chromatography purifications (SiO_2, CH_2Cl_2 to 85:15 CH_2Cl_2:CH_3OH) to yield several fractions containing impure product. The product was further purified by semi-preparative HPLC (Method B) to yield dyad **3** as a blue solid (3.2 mg, 34%); R_f: 0.42 (SiO_2, 90:10 CH_2Cl_2:CH_3OH); ^1H NMR (400 MHz, CD_3OD) δ 8.58 (d, $^3J_{H\text{-}H}$ 8.0, 1H, H^{23}), 8.54 (br s, 1H, H^9), 8.05 (dd, $^3J_{H\text{-}H}$ 8.0, $^4J_{H\text{-}H}$ 1.6, 1H, H^{10}), 7.90 (d, $^3J_{H\text{-}H}$ 8.0, 1H, H^{20}), 7.78 (dd, $^3J_{H\text{-}H}$ 8.4, $^4J_{H\text{-}H}$ 1.4, 1H, H^{13}), 7.75 (s, 1H, H^{18}), 7.64 (d, $^4J_{H\text{-}H}$ 1.4, 1H, H^{15}), 7.58 (t, $^3J_{H\text{-}H}$ 8.0, 1H, H^{22}), 7.37–7.32 (m, 2H, H^8 + H^{21} overlapped), 7.13 (d, $^3J_{H\text{-}H}$ 7.2, 1H, H^{26}), 6.89 (s, 1H, H^{19}), 6.87–6.83 (m, 3H, H^3 + H^{28}), 6.77 (s, 2H, H^1), 6.67–6.62 (m, 2H, H^{14} + H^{27} overlapping), 6.12 (d, $^3J_{H\text{-}H}$ 8.0, 1H, H^{29}), 5.53 (s, 2H, H^2), 3.86 (br s, 2H, H^{24}), 3.76–3.62 (m, 8H, H^6 + H^{11} + H^{12} overlapping), 3.12 (br s, 2H, H^{25}), 2.83 (s, 3H, H^{17}), 1.72 (s, 6H, H^4), 1.47 (s, 12H, H^5), 1.38 (m, 12H, H^7 + H^{16}); UV/Vis (MeOH): λ_{max} (ε) = 586 nm (56,000 mol^{-1} dm^3 cm^{-1}); fluorescence (MeOH): λ_{ex} = 545 nm; λ_{em} = 615 nm; Φ_{fl} = 47%; HRMS (ESI$^+$) *m/z* calcd for $C_{70}H_{70}O_6N_7^+$: 1104.538 [M$^+$]; found: 1104.533.

6’-Atto590-Boc-protected piperazine conjugate (4a)

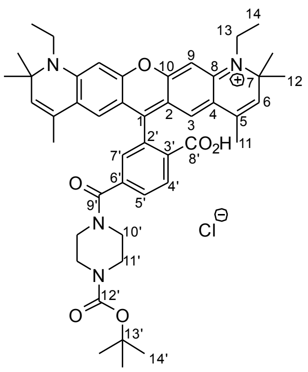

6’-Atto590 (35 mg, 56 µmol) was dissolved in DMF (1.0 mL). To this solution was added HBTU (23 mg, 62 µmol) as a solution in DMF (1.0 mL) and DIPEA (90 µL, 500 µmol) and the solution was stirred for 30 min at room temperature. 1-Boc-piperazine (10.4 mg, 56 µmol) was added as a solution in DMF (0.5 mL) and the reaction mixture was stirred at 20 °C under argon for 10 min, after which the reaction mixture was quenched with water (3 mL) and stirred for a further 15 min. The solvent was removed under reduced pressure and the residue dissolved in dichloromethane (20 mL). The solution was washed with aqueous HCl (2%, 20 mL), and the aqueous layer extracted with dichloromethane (3 × 20 mL). The organic layers were combined, washed with brine (20 mL) and concentrated. The crude product was purified by column chromatography (SiO_2, 98:2 to 88:12 CH_2Cl_2:MeOH) to yield dye **4a** as a deep blue solid (31 mg, 69%); R_f: 0.38 (SiO_2, 90:10 CH_2Cl_2:CH_3OH); ^1H NMR (500 MHz, CD_3OD) δ 8.19 (d, $^3J_{H\text{-}H}$ 8.0, 1H, $H^{4'}$), 7.72 (dd, $^3J_{H\text{-}H}$ 8.0, $^4J_{H\text{-}H}$ 1.5, 1H, $H^{5'}$), 7.38 (d, $^4J_{H\text{-}H}$ 1.5, 1H, $H^{7'}$), 6.90 (s, 2H, H^3), 6.80 (s, 2H, H^9), 5.60 (s, 2H, H^6), 3.77–3.69 (m, 6H, H^{13} + overlapping $H^{10'/11'}$), 3.60–3.35 (br m, 6H, $H^{10'}$ + $H^{11'}$), 1.79 (s, 6H, H^{11}), 1.49 (s, 12H, H^{12}), 1.46 (s, 9H, H^{14}), 1.36 (t, $^3J_{H\text{-}H}$ 7.0, 6H, H^{14}); ^{13}C NMR (126 MHz, CD_3OD) δ 172.4 ($C^{8'}$), 171.4 ($C^{9'}$), 159.5 (C^{10}), 159.3 (C^1), 156.2 ($C^{12'}$), 153.5 (C^8), 142.6 ($C^{6'}$), 138.0 ($C^{2'}$), 134.0 ($C^{3'}$), 133.7 (C^6), 131.5 ($C^{4'}$), 129.4 ($C^{5'}$), 129.3 ($C^{7'}$), 126.7 (C^5), 124.7 (C^4), 123.7 (C^3), 115.2 (C^2), 96.7 (C^9), 80.8 ($C^{13'}$), 48.4 (obscured by solvent residual peak) and 43.2 ($C^{10'}$ and $C^{11'}$), 61.3 (C^7), 41.1 (C^{13}), 29.6 (C^{12}), 28.6 ($C^{14'}$), 18.3 (C^{11}), 13.6 (C^{14}); UV/Vis (MeOH): λ_{max} (ε) = 587 nm (71,000 mol^{-1} dm^3 cm^{-1}); fluorescence (MeOH): λ_{ex} = 545 nm; λ_{em} = 612 nm; Φ_{fl} = 97%; HRMS (ESI^+) *m/z* calcd for $C_{46}H_{55}N_4O_6^+$: 759.4116 [M^+]; found: 759.4113.

6’-Atto590-piperazine-SO dyad (4)

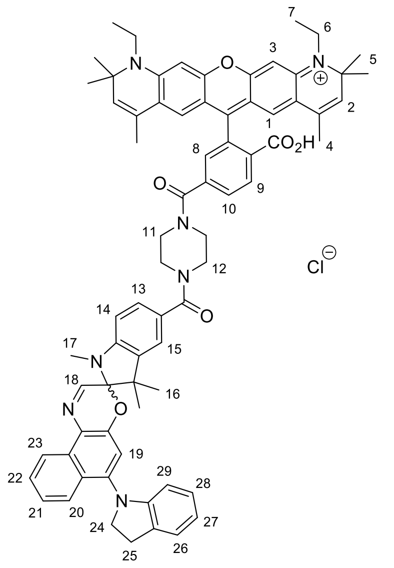

Dye **4a** (31 mg, 39 µmol) was dissolved in dichloromethane (3 mL) and trifluoroacetic acid (0.3 mL) was added. The resulting blue solution was stirred at 20 °C under argon for 45 min. The solvent was removed under high vacuum and the residue was dissolved in dichloromethane. This process was repeated 5 times, to yield the crude product as a blue solid, which was used immediately without further purification (assuming quantitative conversion); R_f: 0.10 (SiO_2, 90:10 CH_2Cl_2:CH_3OH); MS (ESI^+) *m/z* 659.4 $[M]^+$; Analytical HPLC (Method A): t_R = 14.4 min.

SO-COOH (17 mg, 35 µmol) was dissolved in DMF (1.0 mL), and HBTU (13 mg, 35 µmol) and DIPEA (135 µL, 776 µmol) were added as stock solutions in DMF, giving a total solvent volume of 2.0 mL. This solution was stirred at 20 °C under argon. After 1 h, the yellow solution was transferred into a dry Schlenk tube containing crude deprotected dye **4a** (30 mg, 39 µmol). The resulting blue solution was stirred at 20 °C under argon overnight after which time H_2O (3 mL) was added and the solution was stirred for 30 min. The solvent was removed under reduced pressure and the residue was dissolved in dichloromethane (20 mL) and washed with aqueous HCl (2%, 20 mL). The aqueous solution was extracted with dichloromethane (3 × 20 mL), and the organic layers were combined, washed with brine (10 mL), and concentrated. The crude product was purified by column chromatography (SiO_2, CH_2Cl_2 to 90:10 CH_2Cl_2:MeOH) to yield dyad **4** as a dark blue solid (16 mg, 35%); R_f: 0.46 (SiO_2, 90:10 CH_2Cl_2:CH_3OH); ^1H NMR (500 MHz, CD_3OD) δ 8.58 (d, $^3J_{H\text{-}H}$ 8.3, 1H, H^{23}), 8.17 (d, $^3J_{H\text{-}H}$ 7.9, 1H, H^9), 7.91 (d, $^3J_{H\text{-}H}$ 8.4, 1H, H^{20}), 7.76–7.72 (m, 2H, H^{10} + H^{18}), 7.58 (t, $^3J_{H\text{-}H}$ 8.3, 1H, H^{22}), 7.37–7.32 (m, 3H, H^8 + H^{13} + H^{21}), 7.22 (d, $^4J_{H\text{-}H}$ 1.7, 1H, H^{15}), 7.15 (d, $^3J_{H\text{-}H}$ 8.5, 1H, H^{26}), 6.91–6.89 (m, 3H, H^3 + H^{19}), 6.83 (t, $^3J_{H\text{-}H}$ 7.8, 1H, H^{28}), 6.78 (s, 2H, H^1), 6.70–6.63 (m, 2H, H^{14} + H^{27}), 6.14 (d, $^3J_{H\text{-}H}$ 7.8, 1H, H^{29}), 5.56 (s, 2H, H^2), 3.91–3.51 (m, 14H, H^6 + H^{11} + H^{12} + H^{24}), 3.17–3.09 (br s, 2H, H^{25}), 2.81 (s, 3H, H^{17}), 1.76 (s, 6H, H^4), 1.48–1.40 (br m, 12H, H^5), 1.38–1.27 (m, 12H, H^7 + H^{16}); UV/Vis (MeOH): λ_{max} (ε) = 587 nm (77,000 mol^{-1} dm^3 cm^{-1}); fluorescence (MeOH): λ_{ex} = 545 nm; λ_{em} = 613 nm; Φ_{fl} = 66%; HRMS (ESI^+) *m/z* calcd for $C_{72}H_{72}N_7O_6^+$: 1130.554 [M^+]; found: 1130.549.

6’-Atto590-Boc-protected 4,4’-bipiperidine conjugate (5a)

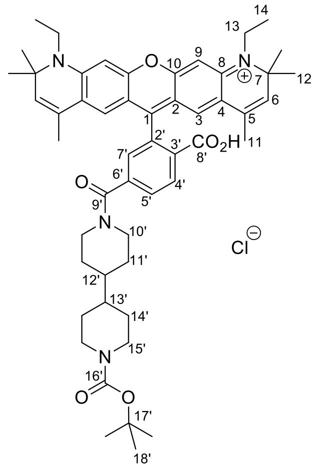

6’-Atto590 (20 mg, 32 µmol) was dissolved in DMF (1.0 mL). To this solution was added HBTU (12 mg, 32 µmol) as a solution in DMF (1.0 mL) and DIPEA (50 µL, 290 µmol) and the solution was stirred for 5 min. *N*-Boc-4,4’-bipiperidine (8.6 mg, 32 µmol) was added as a solution in DMF (0.5 mL) and the reaction mixture was stirred at 20 °C under argon for 20 min after which the reaction mixture was quenched with water (3 mL) and stirred for a further 20 min. The solvent was removed under reduced pressure and the residue dissolved in dichloromethane (20 mL). The solution was washed with aqueous HCl (2%, 20 mL), and the aqueous layer extracted with dichloromethane (3 × 20 ml). The organic layers were combined, washed with brine (20 mL) and concentrated. The crude product was purified by column chromatography (SiO_2, CH_2Cl_2 to 88:12 CH_2Cl_2:MeOH) to yield dye **5a** as a deep blue solid (14.7 mg, 52%); R_f: 0.38 (SiO_2, 90:10 CH_2Cl_2:CH_3OH); ^{1}H NMR (500 MHz, CD_3OD) δ 8.22 (d, $^3J_{H\text{-}H}$ 8.0, 1H, $H^{4'}$), 7.71 (dd, $^3J_{H\text{-}H}$ 8.0, $^4J_{H\text{-}H}$ 1.6, 1H, $H^{5'}$), 7.34 (d, $^4J_{H\text{-}H}$ 1.6, 1H, $H^{7'}$), 6.87 (s, 2H, H^9), 6.81 (s, 2H, H^3), 5.61 (s, 2H, H^6), 4.66 (d, $^2J_{H\text{-}H}$ 12.4, 1H, $H^{10'}$), 4.07 (d, $^2J_{H\text{-}H}$ 12.8, 2H, $H^{15'}$), 3.80–3.69 (m, 5H, H^{13} + $H^{10'}$ overlapping), 3.21–3.17 (m, 1H, $H^{10'}$), 2.84–2.78 (m, 1H, $H^{10'}$), 2.68 (br s, 2H, $H^{15'}$), 1.91–1.85 (m, 1H, $H^{11'}$), 1.78 (s, 6H, H^{11}), 1.74–1.66 (m, 3H, $H^{11'}$ + $H^{14'}$ overlapping), 1.50 (s, 12H, H^{12}), 1.44 (s, 9H, $H^{18'}$), 1.37 (t, $^3J_{H\text{-}H}$ 6.8, 6H, H^{14}); 1.31–1.28 (m, 2H, $H^{11'}$), 1.16–1.06 (m, 3H, $H^{13'}$ + $H^{14'}$ overlapping), 0.94–0.85 (m, 1H, $H^{12'}$); MS (ESI$^+$) *m/z* 841.5 [M]$^+$; UV/Vis (MeOH): λ_{max} (ε) = 586 nm (88,000 mol^{-1} dm^3 cm^{-1}); fluorescence (MeOH): λ_{ex} = 545 nm; λ_{em} = 612 nm; Φ_{fl} = 86%; HRMS (ESI$^+$) *m/z* calcd for $C_{52}H_{65}N_6O_4^+$: 841.4899 [M$^+$]; 841.4892.

6’-Atto590-4,4’-bipiperidine-SO dyad (5)

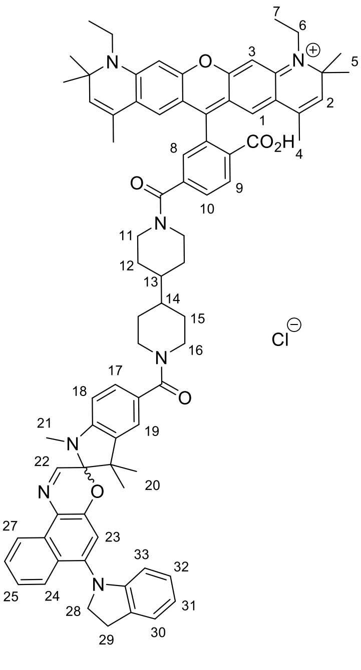

Dye **5a** (10.0 mg, 11.4 µmol) was dissolved in anhydrous dichloromethane (1.8 mL) and trifluoroacetic acid (0.2 mL) was added. The resulting blue solution was stirred at 20 °C under argon for 1 h. The solvent was removed under high vacuum and the residue was dissolved in dichloromethane. This process was repeated 5 times, to yield the crude product as a blue solid, which was used immediately without further purification (assuming quantitative conversion); R_f: 0.11 (SiO_2, 90:10 CH_2Cl_2:CH_3OH); HRMS (ESI$^+$) *m/z* calcd for $C_{47}H_{57}N_4O_4^+$: 741.4374 [M$^+$]; found: 741.4375.

SO-COOH (5.1 mg, 10.3 µmol) was dissolved in anhydrous DMF (0.5 mL), and HBTU (4.3 mg, 11.4 µmol) and DIPEA (17.9 µL, 13.3 mg, 103 µmol) were added as stock solutions in DMF, giving a total solvent volume of 1.5 mL. This solution was stirred at 20 °C under argon. After 1 h, the yellow solution was transferred into a dry Schlenk tube containing crude deprotected dye **5a** (11.0 mg, 11.4 µmol). Further DIPEA (20 µL) was then added. The resulting blue solution was stirred at 20 °C under argon for 23 h, after which time H_2O (3 mL) was added and the solution was stirred for 30 min. The solvent was removed under reduced pressure and the residue was dissolved in dichloromethane (20 mL) and washed with water (20 mL). The aqueous solution was extracted with dichloromethane (3 × 20 mL), and the organic layers were combined, washed with brine (20 mL), and concentrated. The crude product was purified by column chromatography (SiO_2, CH_2Cl_2 to 85:15 CH_2Cl_2:MeOH) to yield some impure product as a dark blue solid (5.7 mg), which was further purified by semi-preparative HPLC (Method B) to yield dyad **5** as a dark blue solid (3.1 mg, 22%); R_f: 0.35 (SiO_2, 90:10 CH_2Cl_2:CH_3OH); ^{1}H NMR (500 MHz, CD_3OD) δ 8.59 (d, $^3J_{H\text{-}H}$ 8.4, 1H, H^{27}), 8.38 (d, $^3J_{H\text{-}H}$ 8.0, 1H, H^9), 7.93 (d, $^3J_{H\text{-}H}$ 8.4, 1H, H^{24}), 7.81 (dd, $^3J_{H\text{-}H}$ 8.0, $^4J_{H\text{-}H}$ 1.6, 1H, H^{10}), 7.76 (s, 1H, H^{22}), 7.59 (t, $^3J_{H\text{-}H}$ 8.4, 1H, H^{26}), 7.48 (d, $^4J_{H\text{-}H}$ 1.6, 1H, H^8), 7.38–7.33 (m, 2H, H^{19} + H^{25}), 7.26 (dd, $^3J_{H\text{-}H}$ 8.0, $^4J_{H\text{-}H}$ 1.6, 1H, H^{17}), 7.18–7.15 (m, 1H, H^{30}), 6.91 (s, 1H, H^{23}), 6.87–6.80 (m, 3H, H^3 + H^{32} overlapping), 6.75 (s, 2H, H^1), 6.71–6.66 (m, 1H, H^{31}), 6.64 (d, $^3J_{H\text{-}H}$ 8.0, 1H, H^{18}), 6.15 (d, $^3J_{H\text{-}H}$ 7.6, 1H, H^{33}), 5.63 (s, 2H, H^2), 4.70–4.67 (m, 1H, linker proton), 3.94–3.85 (m, 2H, linker protons), 3.74–3.72 (m, 4H, H^{28}), 3.25–3.12 (m, 6H, H^6+H^{29}), 2.82 (s, 3H, H^{21}), 2.05–2.00 (m, 1H, linker proton), 1.92–1.84 (m, 2H, linker protons), 1.76 (s, 6H, H^4), 1.50 (s, 12H, H^5), 1.40–1.26 (m, 22H, H^7 + H^{20} + linker protons overlapping), 0.92–0.86 (m, 2H, linker protons); UV/Vis (MeOH): λ_{max} (ε) = 586 nm (77,000 mol^{-1} dm^3 cm^{-1}); fluorescence (MeOH): λ_{ex} = 545 nm; λ_{em} = 611 nm; Φ_{fl} = 58%; HRMS (ESI$^+$) *m/z* calcd for $C_{78}H_{82}N_7O_6^+$: 1212.632 [M$^+$]; found: 1212.627.

Synthesis of 'dummy dyad' 6

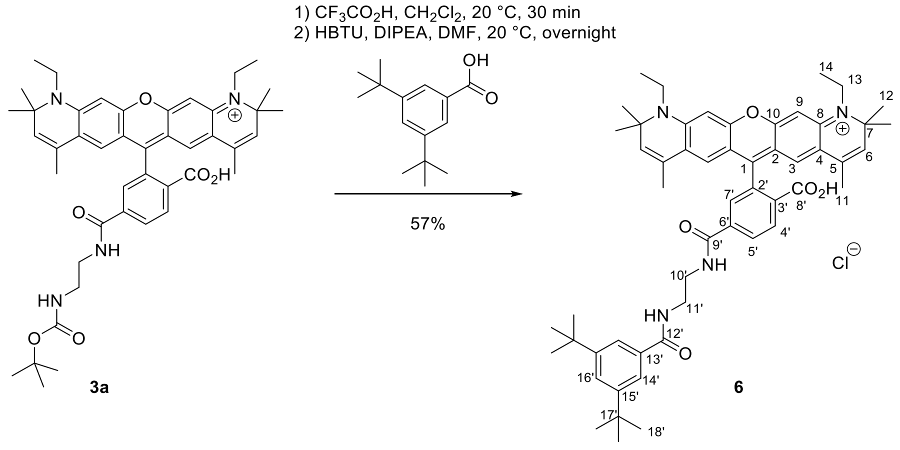

Scheme S4 Synthesis of 'dummy dyad' **6**.

6'-Atto590-ethylenediamine-3,5-di-*tert*-butylbenzoic acid (6)

Dye **3a** (11.8 mg, 15 µmol) was dissolved in anhydrous dichloromethane (1.8 mL) and trifluoroacetic acid (0.2 mL) was added. The resulting purple solution was stirred at 20 °C under argon for 30 min, after which time the solvent was removed under high vacuum. Dichloromethane (1 mL) was added to the residue and the solvent removed under vacuum. This process was repeated 5 times. The resulting blue solid was used immediately without further purification assuming quantitative conversion; R_f: 0.06 (SiO_2, 90:10 CH_2Cl_2:CH_3OH).

3,5-Di-*tert*-butylbenzoic acid (4.3 mg, 18 µmol) was dissolved in anhydrous DMF (0.5 mL), and HBTU (7.0 mg, 18 µmol) and DIPEA (30 µL, 21 mg, 170 µmol) were added as stock solutions in DMF, giving a total solvent volume of 1.5 mL. This solution was stirred at 20 °C under argon. After 1 h, the yellow solution was transferred into a dry Schlenk tube containing crude deprotected dye **3a** (13 mg, 15 µmol). The resulting blue solution was stirred at 20 °C under argon overnight. Water (2 mL) was added and the solution was stirred for 30 min. The solvent was removed under reduced pressure and the residue was dissolved in dichloromethane (20 mL) and washed with dilute aq. HCl (2%, 20 mL). The aqueous solution was extracted with dichloromethane (3 × 20 mL), and the organic layers were combined, washed with brine (20 mL), and concentrated. The crude product was purified by column chromatography (SiO_2, CH_2Cl_2 to 85:15 CH_2Cl_2:MeOH) to give dyad **6** as a dark blue solid (7.8 mg, 57%); R_f: 0.76 (SiO_2, 90:10 CH_2Cl_2:CH_3OH); ^{1}H NMR (500 MHz, CD_3OD) δ 8.16 (d, $^3J_{H\text{-}H}$ 8.0, 1H, $H^{5'}$), 8.10 (dd, $^3J_{H\text{-}H}$ 8.0, $^4J_{H\text{-}H}$ 1.5, 1H, $H^{4'}$), 7.75 (d, $^4J_{H\text{-}H}$ 1.5, 1H, $H^{7'}$), 7.63 (d, $^4J_{H\text{-}H}$ 1.8, 2H, $H^{14'}$), 7.56 ($^4J_{H\text{-}H}$ 1.8, 1H, $H^{16'}$), 6.85 (s, 2H, H^9), 6.79 (s, 2H, H^3), 5.56 (s, 2H, H^6), 3.72 (q, $^3J_{H\text{-}H}$ 7.0, 4H, H^{13}), 3.68–3.66 (m, 2H, $H^{10'}$), 3.63–3.60 (m, 2H, $H^{11'}$), 1.70 (s, 6H, H^{11}), 1.49 (s, 12H, H^{12}), 1.37 (t, $^3J_{H\text{-}H}$ 7.0, 6H, H^{14}), 1.27 (s, 18H, $H^{18'}$); ^{13}C NMR (126 MHz, CD_3OD) δ 171.5 ($C^{12'}$), 169.2 ($C^{9'}$), 159.5 (C^1 and C^{10} overlapping), 152.3 ($C^{15'}$), 153.5 (C^8), 143.9 ($C^{2'}$), 139.2 ($C^{13'}$), 136.6 ($C^{6'}$), 133.7 ($C^{3'}$), 133.6 (C^6), 131.3 ($C^{5'}$), 129.9 ($C^{7'}$), 129.4 ($C^{4'}$), 126.8 (C^5), 126.6 ($C^{16'}$), 124.7 (C^4), 123.9 (C^9), 122.6 ($C^{14'}$), 115.2 (C^2), 96.7 (C^3), 61.4 (C^7), 41.3 ($C^{11'}$), 41.1 (C^{13}), 40.8 ($C^{10'}$), 35.8 ($C^{17'}$), 31.8 ($C^{18'}$), 29.6 (C^{12}), 18.3 (C^{11}), 13.6 (C^{14}); UV/Vis (MeOH): λ_{max} (ε) = 586 nm (102,000 mol^{-1} dm^3 cm^{-1}); fluorescence (MeOH): λ_{ex} = 545 nm; λ_{em} = 611 nm; Φ_{fl} = 91%; HRMS (ESI$^+$) *m/z* calcd for $C_{54}H_{65}N_4O_5^+$: 849.4950 [M$^+$]; found: 849.4949.

Atto590 synthesis

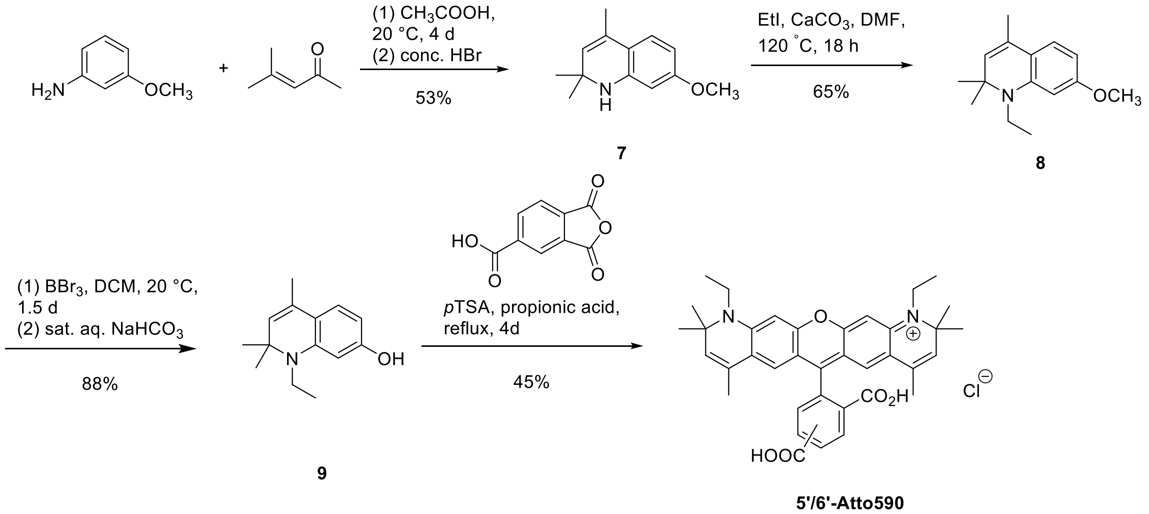

Scheme S5 Synthesis of **5'-** and **6'-Atto590** adapted from reference [4].

7-Methoxy-2,2,4-trimethyl-1,2-dihydroquinoline (7)

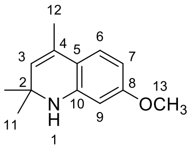

Acetic acid (0.54 mL, 9.0 mmol) was added slowly to *m*-anisidine (5.2 mL, 46 mmol) with stirring. Mesityl oxide (5.4 mL, 46 mmol) was added slowly and the reaction mixture was stirred at 20 °C for 6 h, after which time further mesityl oxide (3.0 mL, 26 mmol) was added. The reaction was left to stir at 20 °C for a further 4 days. Hydrobromic acid (10 mL of 33% wt solution in AcOH) was added and the resulting green precipitate was collected by filtration and washed with acetone. The solid was suspended in water (20 mL) and the pH adjusted to 7 using aqueous sodium hydroxide (10 M). The aqueous solution was extracted with chloroform (3 × 50 mL) and the organic layers were combined, dried over sodium sulfate, filtered and concentrated under reduced pressure to yield a dark green solid, which was recrystallized from petroleum ether to yield **7** as pale brown crystals (5.01 g, 53%); R_f 0.52 (SiO_2, 90:10 petroleum ether:ethyl acetate); ^{1}H NMR (400 MHz, $CDCl_3$) δ 6.98 (d, $^3J_{H\text{-}H}$ 8.5, 1H, H^6), 6.21 (dd, $^3J_{H\text{-}H}$ 8.5, $^4J_{H\text{-}H}$ 2.5, 1H, H^7), 6.02 (d, $^4J_{H\text{-}H}$ 2.5, 1H, H^9), 5.20 (s, 1H, H^3), 3.75 (s, 3H, H^{13}), 3.70 (br s, 1H, H^1), 1.97 (s, 3H, H^{12}), 1.27 (s, 6H, H^{11}); ^{13}C NMR (101 MHz, $CDCl_3$) δ 160.3 (C^8), 144.7 (C^{10}), 128.3 (C^4), 126.1 (C^3), 124.8 (C^6), 115.5 (C^5), 102.4 (C^7), 98.7 (C^9), 55.2 (C^{13}), 52.1 (C^2), 31.2 (C^{11}), 18.8 (C^{12}); HRMS (ESI$^+$) *m/z* calcd for $C_{13}H_{18}NO^+$: 204.1383 [M+H$^+$]; found: 204.1382.

1-Ethyl-7-methoxy-2,2,4-trimethyl-1,2-dihydroquinoline (8)

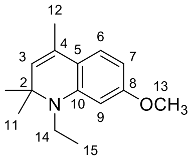

7-Methoxy-2,2,4-trimethyl-1,2-dihydroquinoline **7** (1.75 g, 8.6 mmol) was dissolved in anhydrous DMF (15 mL). Calcium carbonate (1.03 g, 10.3 mmol) and ethyl iodide (0.83 mL, 1.61 g, 10.3 mmol) were subsequently added and the suspension was stirred at 120 °C for 18 h. The reaction mixture was cooled to 20 °C and poured into water (100 mL). The resulting suspension was filtered through a Celite pad. The filtrate was discarded and the solid was washed through the pad with chloroform. This solution was dried over sodium sulfate, filtered and concentrated to yield a dark green oil which was purified by silica gel column chromatography (neat CH_2Cl_2), giving **8** as a clear colorless oil (1.29 g, 65%); R_f 0.75 (SiO_2, 90:10 petroleum ether:ethyl acetate); ^{1}H NMR (400 MHz, $CDCl_3$) δ 6.97 (d, $^3J_{H\text{-}H}$ 8.4, 1H, H^6), 6.15 (dd, $^3J_{H\text{-}H}$ 8.4, $^4J_{H\text{-}H}$ 2.4, 1H, H^7), 6.09 (d, $^4J_{H\text{-}H}$ 2.4, 1H, H^9), 5.11 (d, $^4J_{H\text{-}H}$ 1.2, 1H, H^3), 3.80 (s, 3H, H^{13}), 3.31 (q, $^3J_{H\text{-}H}$ 7.2, 2H, H^{14}), 1.95 (d, $^4J_{H\text{-}H}$ 1.2, 3H, H^{12}), 1.31 (s, 6H, H^{11}), 1.21 (t, $^3J_{H\text{-}H}$ 7.2, 3H, H^{15}); ^{13}C NMR (101 MHz, $CDCl_3$) δ 160.6 (C^8), 145.1 (C^{10}), 127.5 (C^4), 127.1 (C^3), 124.6 (C^6), 116.7 (C^5), 98.9 (C^7), 97.6 (C^9), 57.0 (C^2), 55.2 (C^{13}), 38.3 (C^{14}), 28.8 (C^{11}), 18.9 (C^{12}), 14.4 (C^{15}); MS (ESI$^+$) *m/z* 232 [M+H]$^+$; HRMS (ESI$^+$) *m/z* calcd for $C_{15}H_{23}NO^+$: 232.1696 [M$^+$]; found: 232.1693.

1-Ethyl-2,2,4-trimethyl-1,2-dihydroquinolin-7-ol (9)

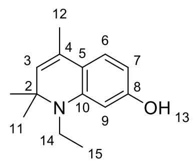

1-Ethyl-7-methoxy-2,2,4-trimethyl-1,2-dihydroquinoline **8** (263 mg, 1.14 mmol) was dissolved in anhydrous dichloromethane (50 mL) and cooled to –78 °C under argon. Boron tribromide (2.51 mL of a 1.0 M solution in CH_2Cl_2, 2 eq) was added. The cooling bath was removed and the resulting orange solution was stirred for 20 h. TLC showed the presence of starting material so further BBr_3 solution (0.5 mL of a 1 M solution in CH_2Cl_2) was added and the reaction mixture was stirred for a further 18 h. The reaction mixture was added dropwise into saturated aqueous $NaHCO_3$ (50 mL). The organic layer was collected and the aqueous layer extracted with dichloromethane (3 × 50 mL). The organic layers were combined, dried over Na_2SO_4, filtered and concentrated to yield **9** as an orange oil which solidified under high vacuum (216 mg, 88%) and was used without further purification; R_f 0.32 (SiO_2, 90:10 petroleum ether:ethyl acetate); ^{1}H NMR (400 MHz, $CDCl_3$) δ 6.89 (d, $^3J_{H\text{-}H}$ 8.0, 1H, H^6), 6.07 (dd, $^3J_{H\text{-}H}$ 8.0, $^4J_{H\text{-}H}$ 2.4, 1H, H^7), 6.05 (d, $^4J_{H\text{-}H}$ 2.4, 1H, H^7), 5.08 (d, $^4J_{H\text{-}H}$ 1.2, 1H, H^3), 3.28 (q, $^3J_{H\text{-}H}$ 7.2, 2H, H^{14}), 1.93 (d, $^4J_{H\text{-}H}$ 1.2, 3H, H^{12}), 1.30 (s, 6H, H^{11}), 1.19 (t, $^3J_{H\text{-}H}$ 7.2, 3H, H^{15}); ^{13}C NMR (101 MHz, $CDCl_3$) δ 156.5 (C^8), 145.4 (C^{10}), 131.5 (C^4), 127.5 (C^5), 126.8 (C^3), 124.9 (C^6), 101.6 (C^7), 97.7 (C^9), 57.1 (C^2), 38.4 (C^{14}), 28.8 (C^{11}), 18.9 (C^{12}), 14.4 (C^{15}); ESI$^+$ *m/z* 218.2 $[M+H]^+$.

5'- and 6'-Atto590

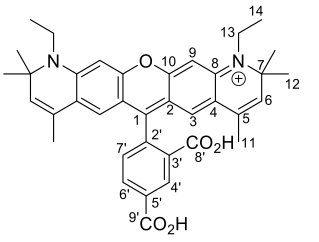

5'-Atto590

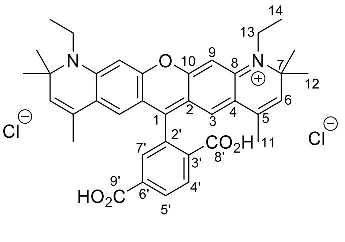

6'-Atto590

1-Ethyl-2,2,4-trimethyl-1,2-dihydroquinolin-7-ol **9** (1.44 g, 6.6 mmol), trimellitic anhydride (0.64 g, 3.3 mmol) and *p*-toluenesulfonic acid (63 mg, 0.3 mmol) were dissolved in propionic acid (5.0 mL). The mixture was stirred at reflux for 4 d under argon after which time it was cooled to 20 °C and poured into 2% aqueous HCl (100 mL) and extracted with dichloromethane (4 x 100 mL). All the organic layers were combined and concentrated to give a dark blue solid (2.02 g). The crude material was purified by column chromatography (SiO_2, 97:2:1 CH_2Cl_2:CH_3OH:NEt_3 to 82:15:3 CH_2Cl_2:CH_3OH:NEt_3) in small batches (approx. 200 mg) to yield dark blue solids for both isomers (90 mg, 45% total yield).

5'-Atto590: R_f: 0.43 (SiO_2, 87:10:3 CH_2Cl_2:CH_3OH:NEt_3); ^{1}H NMR (500 MHz, CD_3OD) δ 8.84 (d, $^4J_{H\text{-}H}$ 1.6, 1H, $H^{4'}$), 8.37 (dd, $^3J_{H\text{-}H}$ 8.0, $^4J_{H\text{-}H}$ 1.6, 1H, $H^{6'}$), 7.51 (d, $^3J_{H\text{-}H}$ 8.0, 1H, $H^{7'}$), 6.82 (s, 2H, H^9), 6.77 (s, 2H, H^3), 5.61 (br s, 2H, H^6), 3.73 (q, $^3J_{H\text{-}H}$ 7.2, 4H, H^{13}), 1.75 (s, 6H, H^{11}), 1.50 (s, 12H, H^{12}), 1.37 (t, $^3J_{H\text{-}H}$ 7.2, 6H, H^{14}); ^{13}C NMR (126 MHz, CD_3OD) δ 169.2 ($C^{8'}$), 168.6 ($C^{9'}$), 159.4 (C^{10}), 157.7 ($C^{2'}$), 153.6 (C^8), 138.3 (C^1), 136.2 ($C^{3'}$), 134.7 ($C^{5'}$), 134.0 (C^6), 133.5 ($C^{6'}$), 132.9 ($C^{4'}$), 131.7 ($C^{7'}$), 126.5 (C^2), 125.0 (C^5), 123.0 (C^3), 114.9 (C^4), 96.8 (C^9), 61.5 (C^7), 41.2 (C^{13}), 29.6 (C^{12}), 18.2 (C^{11}), 13.6 (C^{14}); UV/Vis (MeOH): λ_{max} (ε) = 583 nm (85,000 $mol^{-1}\,dm^3\,cm^{-1}$); fluorescence (MeOH): λ_{ex} = 545 nm; λ_{em} = 608 nm; Φ_{fl} = 89%; HRMS (ESI$^+$) *m/z* calcd for $C_{37}H_{39}N_2O_5^+$: 591.2854 [M^+]; found: 591.2852.

6'-Atto590: R_f: 0.59 (SiO_2, 87:10:3 CH_2Cl_2:CH_3OH:NEt_3); ^{1}H NMR (500 MHz, CD_3OD) δ 8.38 (m, 2H, $H^{4'}$ + $H^{5'}$ overlapping), 8.04 (d, $^4J_{H\text{-}H}$ 1.0, 1H, $H^{7'}$), 6.85 (s, 2H, H^9), 6.74 (s, 2H, H^3), 5.63 (d, $^4J_{H\text{-}H}$ 1.2, 2H, H^6), 3.75 (q, $^3J_{H\text{-}H}$ 7.2, 4H, H^{13}), 1.74 (d, $^4J_{H\text{-}H}$ 1.2, 6H, H^{11}), 1.51 (s, 12H, H^{12}), 1.38 (t, $^3J_{H\text{-}H}$ 7.2, 6H, H^{14}); ^{13}C NMR (126 MHz, CD_3OD) δ 168.0 ($C^{8'}$), 167.7 ($C^{9'}$), 159.5 (C^{10}), 156.4 ($C^{2'}$), 153.7 (C^8), 136.8 (C^1), 135.9 ($C^{3'}$), 134.8 ($C^{6'}$), 134.2 (C^6), 132.5 ($C^{4'}$ + $C^{5'}$), 132.4 ($C^{7'}$), 126.4 (C^2), 125.1 (C^5), 122.6 (C^3), 115.0 (C^4), 97.0 (C^9), 61.6 (C^7), 41.3 (C^{13}), 29.6 (C^{12}), 18.2 (C^{11}), 13.6 (C^{14}); UV/Vis (MeOH): λ_{max} (ε) = 583 nm (73,000 $mol^{-1}\,dm^3\,cm^{-1}$); fluorescence (MeOH): λ_{ex} = 545 nm; λ_{em} = 606 nm; Φ_{fl} = 92%; HRMS (ESI$^+$) *m/z* calcd for $C_{37}H_{39}N_2O_5^+$: 591.2854 [M^+]; found: 591.2852.

Spironaphthoxazine NHS ester (SO-NHS)

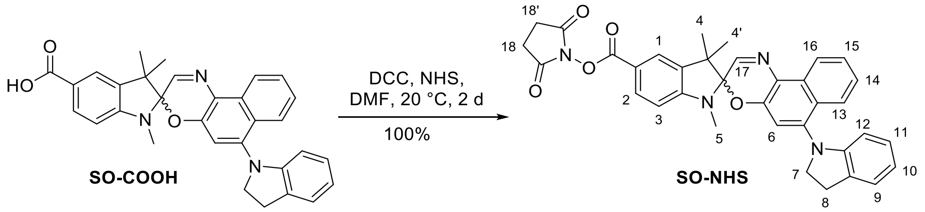

Scheme S6 Conversion of **SO-COOH** to **SO-NHS.**

A solution of **SO-COOH** (10 mg, 0.02 mmol), *N*-hydroxysuccinimide (3.5 mg, 0.03 mmol), *N,N'*-dicyclohexylcarbodiimide (6.2 mg, 0.03 mmol) in DMF (0.5 mL) was stirred at 20 °C overnight. To the mixture was added *N*-hydroxysuccinimide (3.5 mg, 0.03 mmol), *N,N'*-dicyclohexylcarbodiimide (6.2 mg, 0.03 mmol) and the solution was allowed to stir at 20 °C for 1 d. The solution was concentrated and the crude product was purified using column chromatography (silica, 100% CH_2Cl_2 to 95:5 CH_2Cl_2/EtOAc) yielding a pale yellow solid **SO-NHS** (13.2 mg, 100 %). ^{1}H NMR (400 MHz, $CDCl_3$) δ 8.61 (ddd, $^3J_{H,H}$ = 8.3, 1.3, 0.8 Hz, 1H, H^{16}), 8.07 (dd, $^3J_{H,H}$ = 8.3, 1.7 Hz, 1H, H^2), 7.97 (dt, $^3J_{H,H}$ = 8.3, 1.0, 1.0 Hz, 1H, H^{13}), 7.79 (d, $^3J_{H,H}$ = 1.7 Hz, 1H, H^1), 7.67 (s, 1H, H^{17}), 7.61 (ddd, $^3J_{H,H}$ = 8.3, 6.8, 1.3 Hz, 1H, H^{15}), 7.37 (ddd, $^3J_{H,H}$ = 8.3, 6.8, 1.3 Hz, 1H, H^{14}), 7.20 (d, $^3J_{H,H}$ = 7.4 Hz, 1H, H^9), 6.99 – 6.87 (m, 2H, H^6 + H^{11}), 6.75 (td, $^3J_{H,H}$ = 7.4, 1.0 Hz, 1H, H^{10}), 6.60 (d, $^3J_{H,H}$ = 8.3 Hz, 1H, H^3), 6.31 (d, $^3J_{H,H}$ = 7.8 Hz, 1H, H^{12}), 4.02 – 3.87 (br. s, 2H, H^7), 3.29 – 3.07 (br. s, 2H, H^8), 2.97 – 2.82 (m, 6H, H^5, H^{18}, $H^{18'}$), 1.39 (s, 6H, H^4, $H^{4'}$); HRMS (ESI$^+$) *m/z* calcd for $C_{35}H_{31}N_4O_5^+$: 587.2289 [M+H$^+$]; found: 587.2287.

3. Photophysical Properties

Table S1 Key photophysical properties of Boc-protected dyes and dye **Cy3.5 NHS**, dye **2c** and **6'-Atto590** (MeOH, 298 K).

Compound	λ_{max} abs / nm	λ_{max} em / nm	$\varepsilon(\lambda_{max})$ / mol^{-1} dm^3 cm^{-1}	τ / ns	Φ_{fl} / %	Brightness / mol^{-1} dm^3 cm^{-1}
Dyad **1**	589	604[a]	56000	0.9	9	5040
Dyad **2**	587	607[a]	97000	< 0.6	11	10670
Dyad **3**	586	615[b]	56000	3.3	47	26320
Dyad **4**	587	613[b]	77000	4.0	66	50820
Dyad **5**	586	611[b]	77000	3.8	58	44660
'Dummy Dyad' **6**	586	611[b]	102000	4.3	91	92820
Dye **1a**	587	604[a]	77000	1.3	7	5390
Dye **2d**	590	607[a]	101000	< 0.6	15	15150
Dye **3a**	586	612[b]	123000	4.3	87	107010
Dye **4a**	587	612[b]	71000	4.3	97	68870
Dye **5a**	586	612[b]	88000	4.3	86	75680
**Cy3.5 NHS**	589 [a]	607 [a], [c]	n.d.	n.d.	12[c]	–
Dye **2c**	590	606[a]	91000	< 0.6	12	10920
**6'-Atto590**	583	606[b]	73000	4.1	92	67160

[a] λ_{ex} = 500 nm; [b] λ_{ex} = 545 nm; [c] measured in ethanol

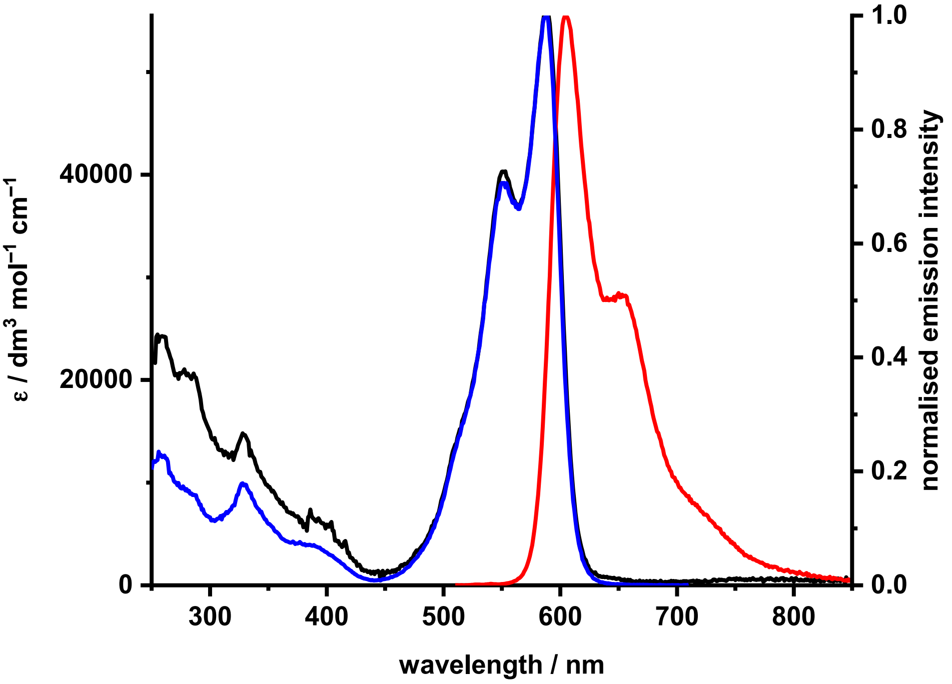

Figure S1 Absorption (black), excitation (blue) and emission (red) spectra of dyad **1** (MeOH, 298 K, λ_{ex} 500 nm, λ_{em} 720 nm).

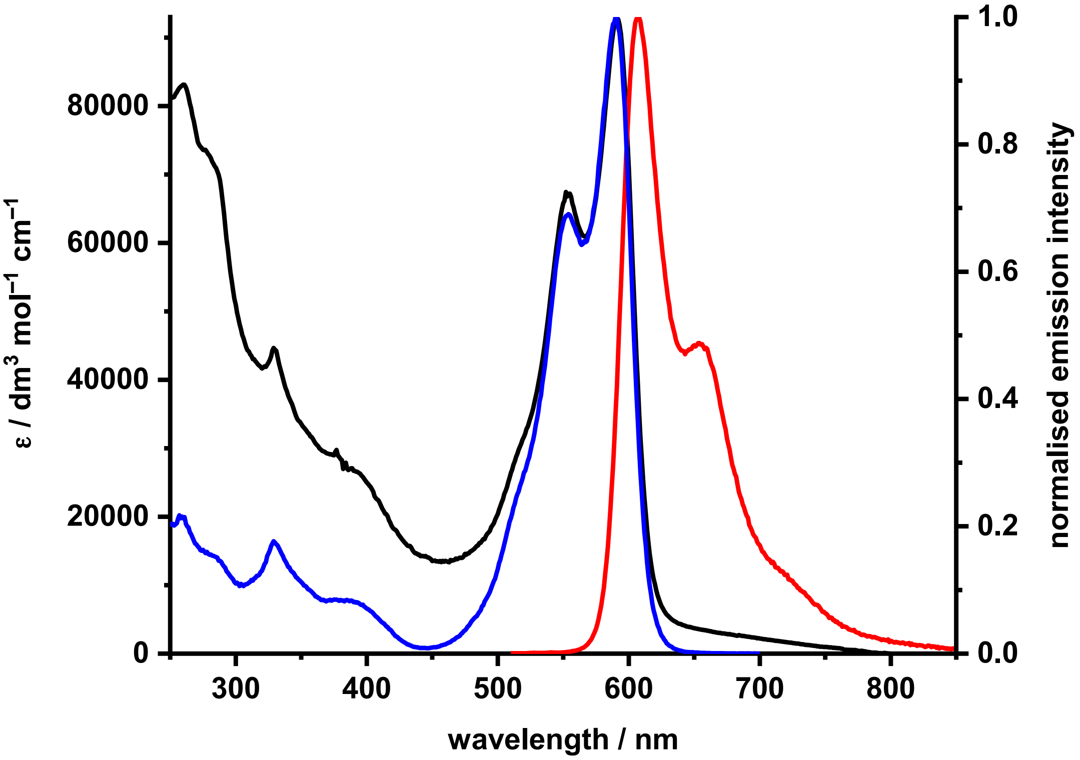

Figure S2 Absorption (black), excitation (blue) and emission (red) spectra of dyad **2** (MeOH, 298 K, λ_{ex} 500 nm, λ_{em} 720 nm).

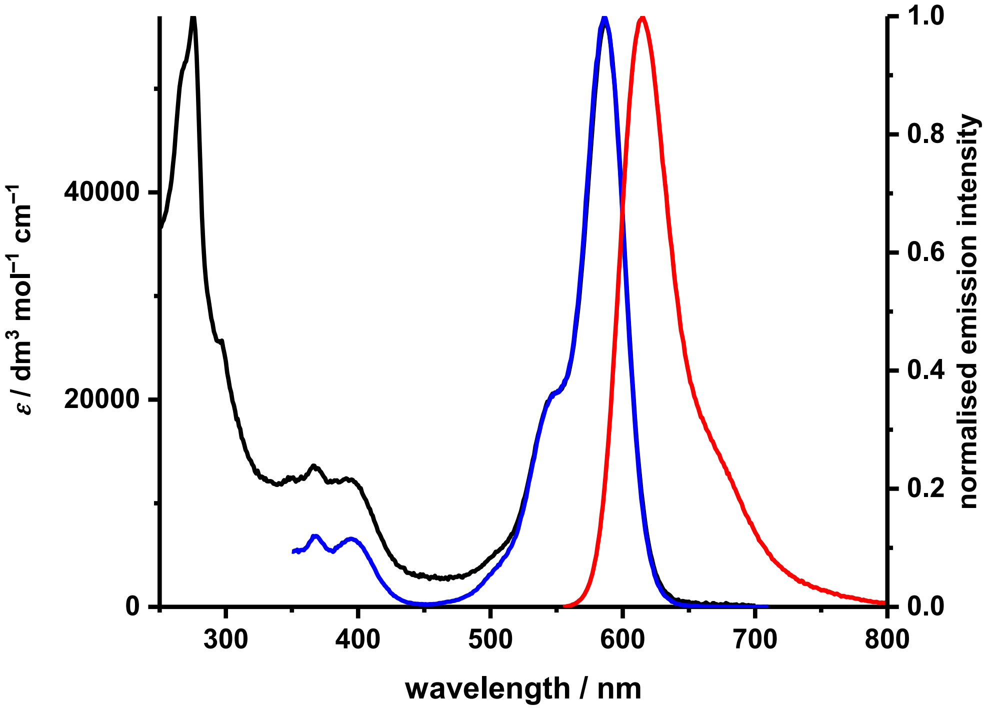

Figure S3 Absorption (black), excitation (blue) and emission (red) spectra of dyad **3** (MeOH, 298 K, λ_{ex} 545 nm, λ_{em} 720 nm).

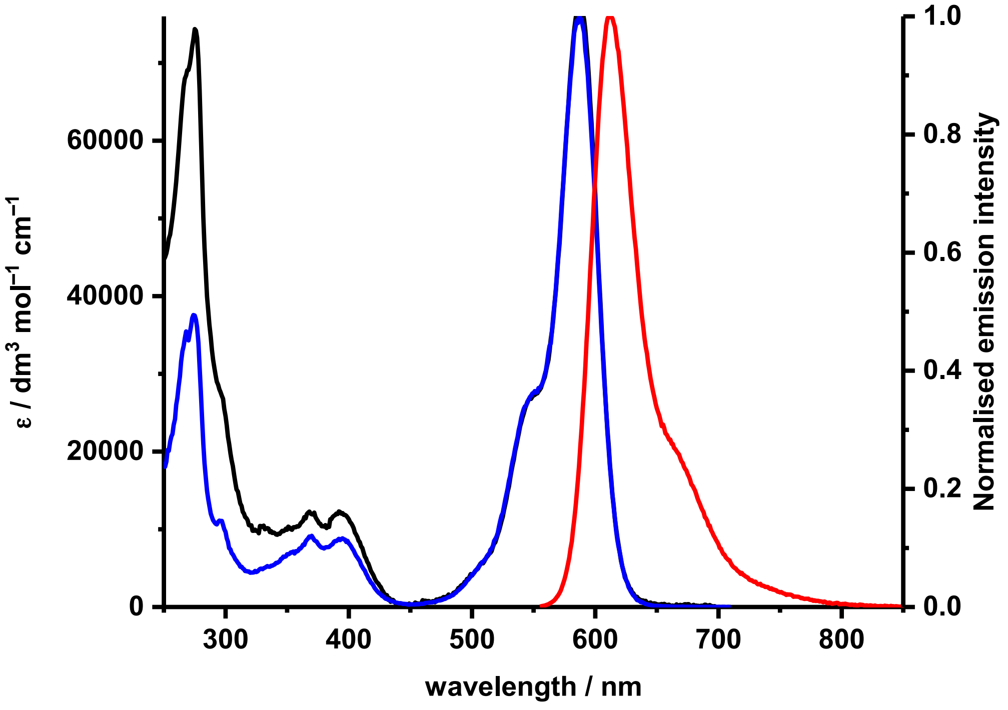

Figure S4 Absorption (black), excitation (blue) and emission (red) spectra of dyad **4** (MeOH, 298 K, λ_{ex} 545 nm, λ_{em} 720 nm).

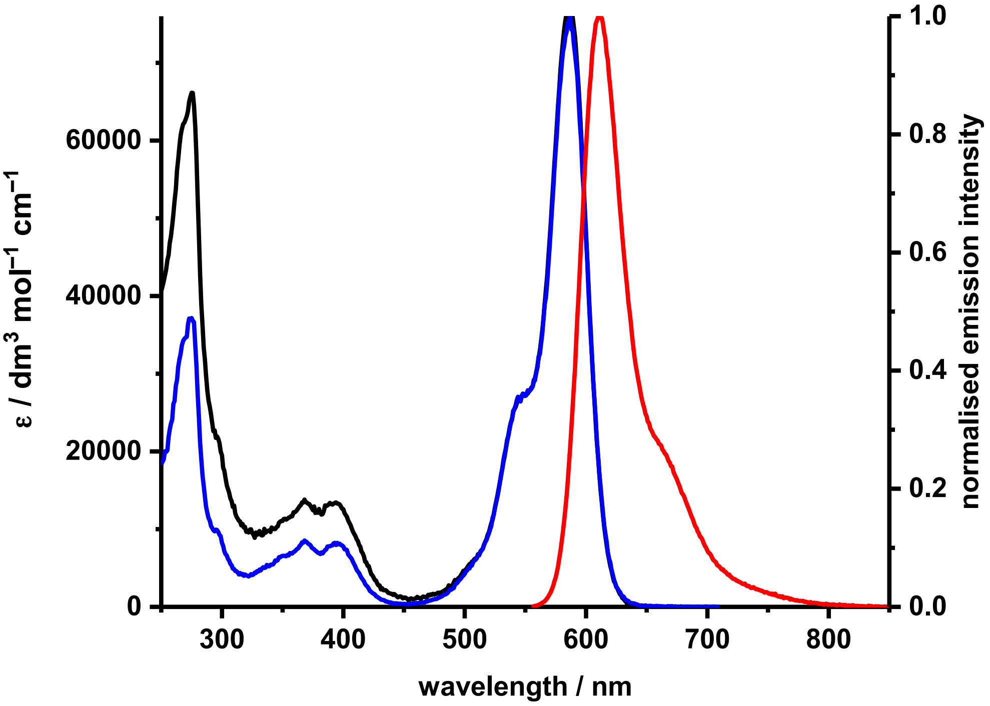

Figure S5 Absorption (black), excitation (blue) and emission (red) spectra of dyad **5** (MeOH, 298 K, λ_{ex} 545 nm, λ_{em} 720 nm).

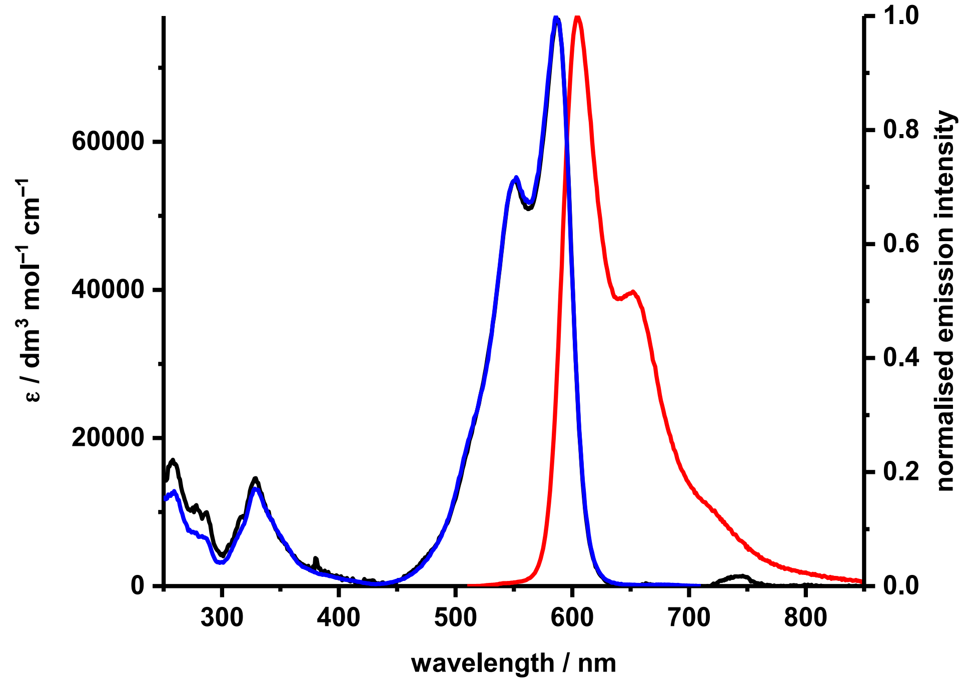

Figure S6 Absorption (black), excitation (blue) and emission (red) spectra of dye **1a** (MeOH, 298 K, λ_{ex} 500 nm, λ_{em} 720 nm).

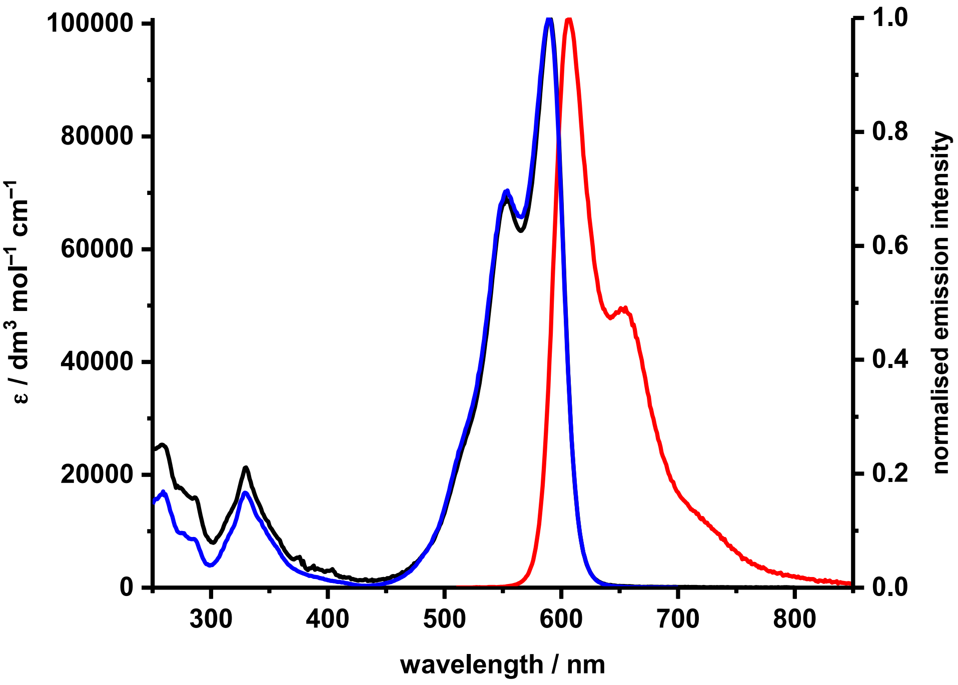

Figure S7 Absorption (black), excitation (blue) and emission (red) spectra of dye **2d** (MeOH, 298 K, λ_{ex} 500 nm, λ_{em} 720 nm).

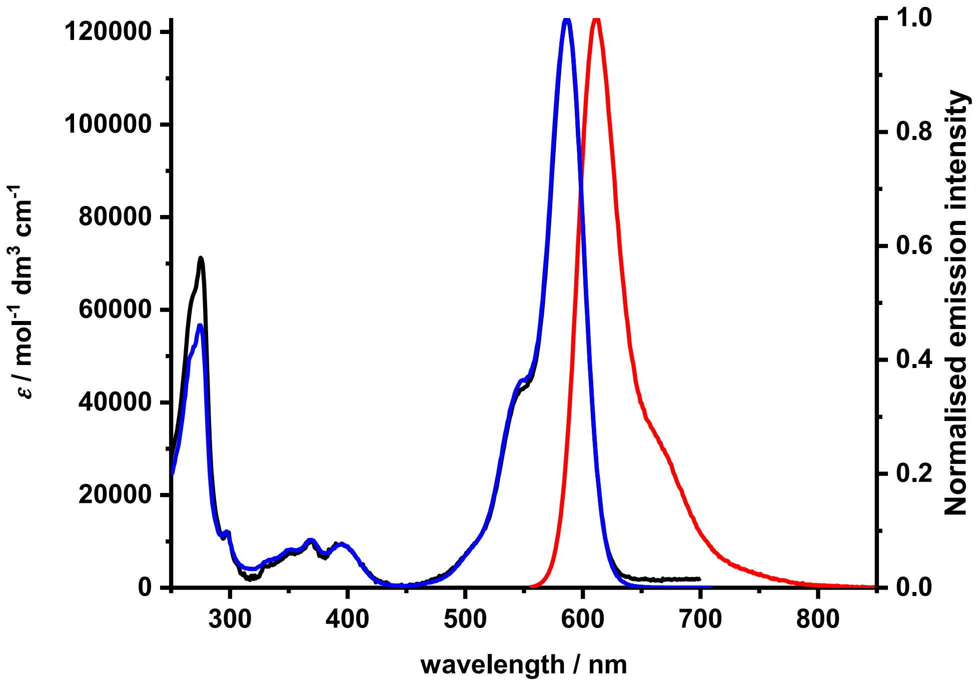

Figure S8 Absorption (black), excitation (blue) and emission (red) spectra of dye **3a** (MeOH, 298 K, λ_{ex} 545 nm, λ_{em} 720 nm).

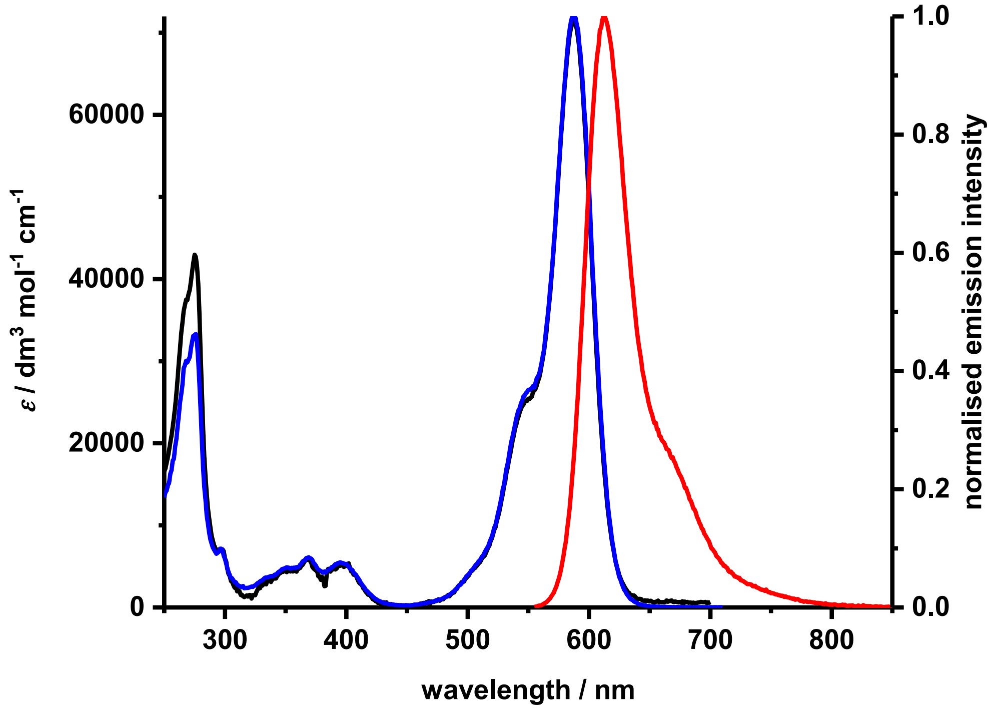

Figure S9 Absorption (black), excitation (blue) and emission (red) spectra of dye **4a** (MeOH, 298 K, λ_{ex} 545 nm, λ_{em} 720 nm).

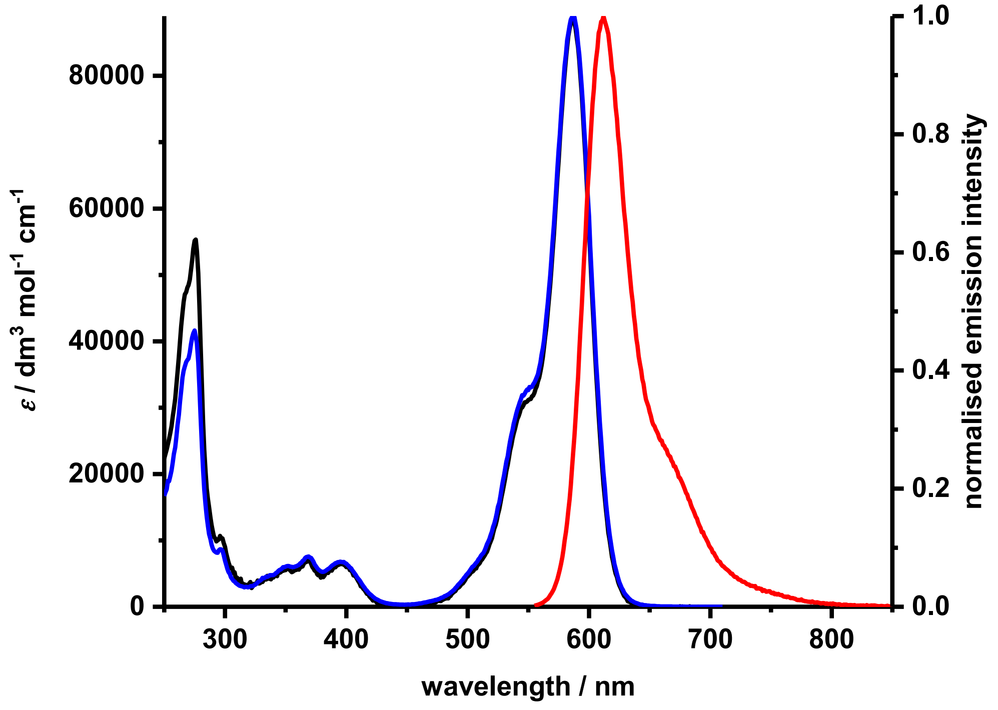

Figure S10 Absorption (black), excitation (blue) and emission (red) spectra of dye **5a** (MeOH, 298 K, λ_{ex} 545 nm, λ_{em} 720 nm).

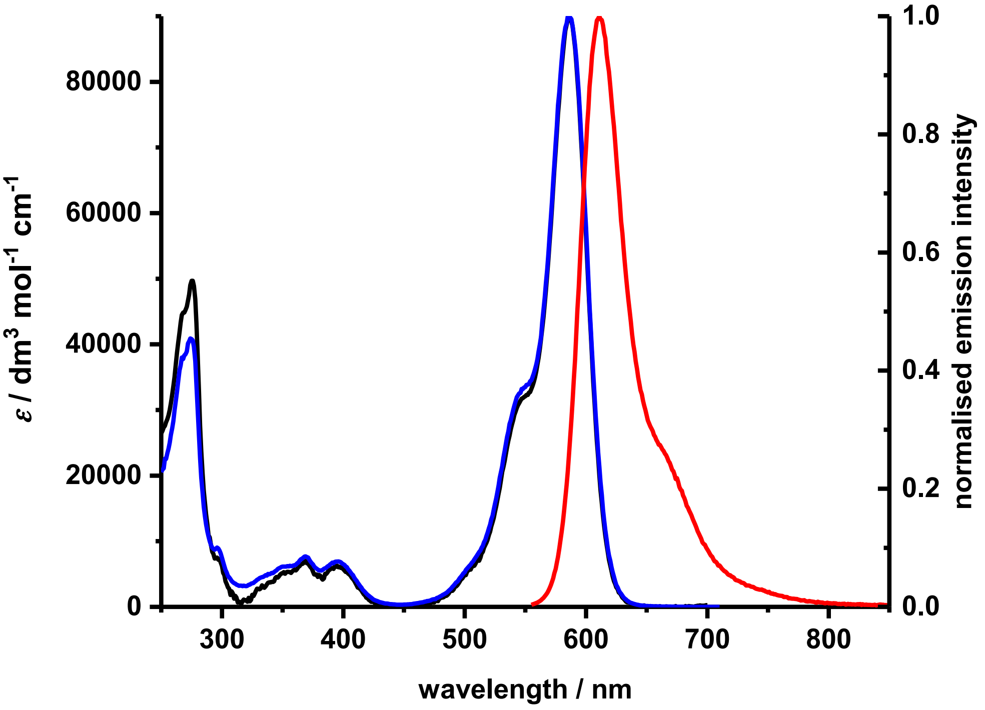

Figure S11 Absorption (black), excitation (blue) and emission (red) spectra of dye **6** (MeOH, 298 K, λ_{ex} 545 nm, λ_{em} 720 nm).

4. Calculation of Förster Distances

Equation S1 was used to calculate Förster radii, R_0, for the dyads (see Table S2):

$$R_0 = 0.2108\left[\frac{\kappa^2 \Phi_D J(\lambda)}{n^4}\right]^{\frac{1}{6}} \qquad \text{(Eq. S1)}$$

where κ^2 is the dipole orientation factor (assumed to be 2/3 given free rotation between the donor and the acceptor), Φ_D is the fluorescence quantum yield of the donor in the absence of the acceptor (in this case, dyes with linkers attached), n is the refractive index of the solvent (1.33 for methanol, 1.34 for PBS buffer), and $J(\lambda)$ is the spectral overlap integral, which is given by Equation S2.

$$J(\lambda) = \int f_D \varepsilon_A(\lambda)\lambda^4 \, d\lambda \qquad \text{(Eq. S2)}$$

where f_D is the normalized donor emission spectrum, $\varepsilon_A(\lambda)$ is the acceptor molar extinction coefficient at wavelength λ. Efficiency of the FRET process, E, can be calculated from the Förster radius, R_0, and the distance between the donor and acceptor, r, according to Equation S3. We have used distances measured from molecular mechanics structural optimizations of the dyads.

$$E = \frac{1}{1 + \left(\frac{r}{R_0}\right)^6} \qquad \text{(Eq. S3)}$$

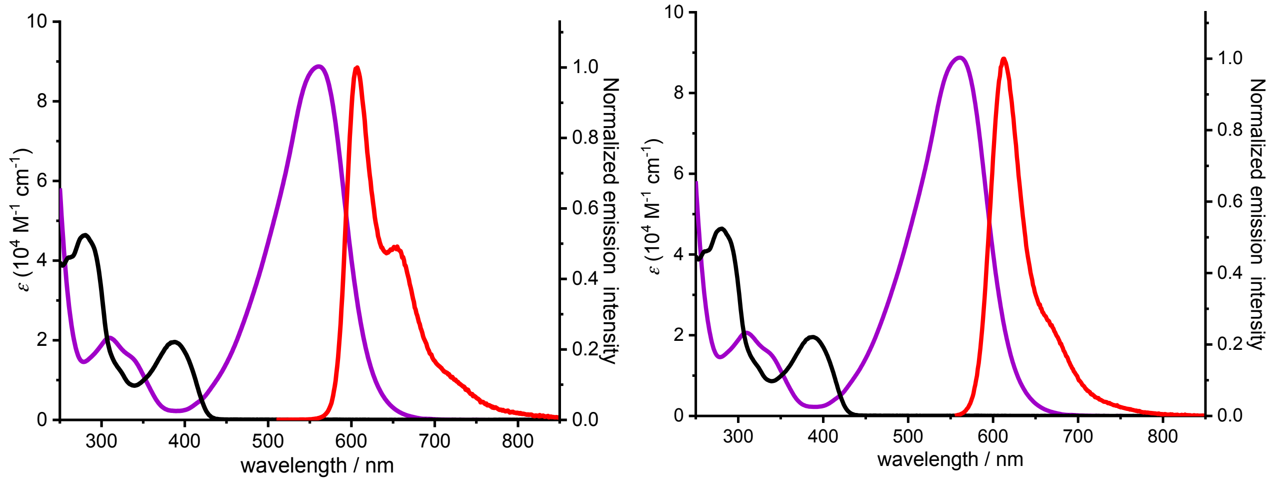

Figure S12 Representative spectral overlap between the absorption open merocyanine form of **SO** (purple) and the emission spectrum (red) of dye **2d** (left) and dye **4a** (right), and spectrum of the closed (spiro) form of **SO** (black)

Table S2 Förster radii and FRET efficiencies for dyads **1**–**5**

Compound	R_0 / Å	r / Å	Calc. FRET efficiency / %
Dyad **1**	37.5	18.4	98.63
Dyad **2**	42.2	18.6	99.28
Dyad **3**	58.0	15.7	99.96
Dyad **4**	59.1	14.3	99.98
Dyad **5**	57.9	18.5	99.89

5. Photoinduced Electron Transfer as a Potential Quenching Mechanism

Experimental redox potential measurements

Redox potentials of Cy3.5, Atto590 and the spironaphthoxazine switch **SO** (Figure S13) were measured using square wave voltammetry. Electrochemical experiments were performed in acetonitrile at compound concentrations of 0.1–1.0 mM with tetrabutylammonium hexafluorophosphate as the electrolyte (0.1 M). Square wave voltammograms were collected with a modulation amplitude of 50 mV and a frequency of 2 Hz. Glassy carbon (Ø = 1 mm), platinum wire and Ag|$AgNO_3$ (10 mM) were used as working, counter and reference electrodes, respectively. The potentials were referenced at the end of each experiment by addition of ferrocene.

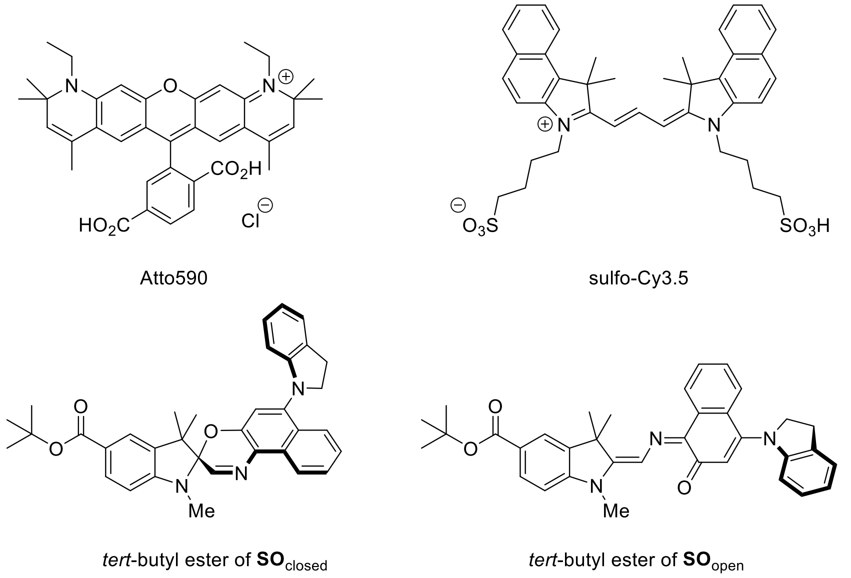

Figure S13 Structures of the dye derivatives and spironaphthoxazine *tert*-butyl ester used for electrochemistry measurements.

Calculated redox potentials

Density functional (DFT) calculations were carried out using the ORCA 4.1.1 program,[5] on simplified chemical structures from the dyads, using previously-published analysis to guide structure conformation (Figure S14).[6] Tight optimization criteria were used for all geometries, and numerical frequency calculations used to confirm stationary points as minima, and to calculate thermal energy corrections. Resolution of identity was used to speed up the SCF process employing the RIJCOSX approximation. Grid6 and GridX6 were used in all calculations. Implicit solvation was introduced employing the SMD model.

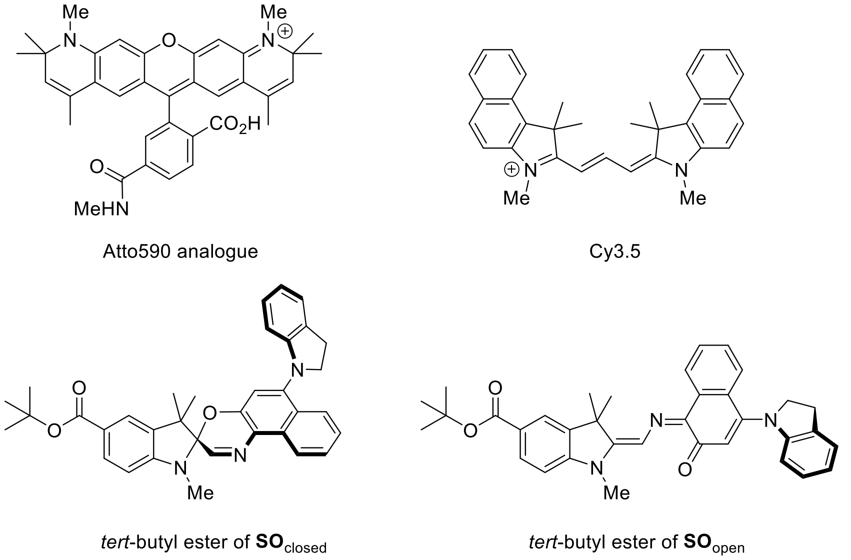

Figure S14 Structures of the dye derivatives and spironaphthoxazine used for calculations.

Redox potentials were calculated from free energies using Equation S4, where z is the number of transferred electrons (z = 1) and F is the Faraday constant, and referenced to the SCE electrode with an absolute E° of 4.429 V, corrected for the liquid junction potential in acetonitrile.[7] These values were further corrected to ferrocene (0.40 V vs. SCE)[8] for comparison with experimental values. Free energies can be calculated in the gas phase and in solvent, and the change in free energy for the redox process calculated according to Equation S5, as illustrated in the Born-Haber Cycle (Figure S15).[7] However, this process can be simplified, and the free energies in solvent of the two species directly calculated and compared, as for the example of a reduction in Equation S6. Calculations were performed at the PBE0 level of theory in combination with the def2-TZVPP basis set and the SMD solvation model (acetonitrile), using the optimized PBE/def2-SVP geometries.

$$E^{\circ} = -\Delta G^{\circ}(\mathrm{sln, redox})/zF \quad \text{(Eq. S4)}$$

$$\Delta G^{\circ}(\mathrm{MeCN, redox}) = \Delta G^{\circ}(\mathrm{gas, redox}) + \Delta G^{\circ}(\mathrm{solv, red}) - \Delta G^{\circ}(\mathrm{solv, ox}) \quad \text{(Eq. S5)}$$

$$\Delta G^{\circ}(\mathrm{MeCN, redox}) = G^{\circ}X^{-}(\mathrm{MeCN}) - G^{\circ}X(\mathrm{MeCN}) \quad \text{(Eq. S6)}$$

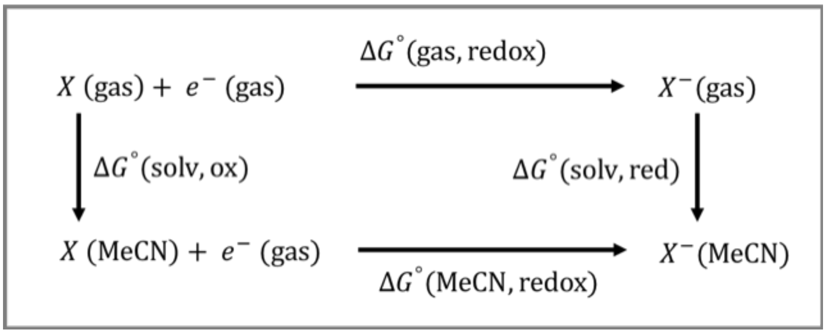

Figure S15 Born-Haber cycle for calculating the reduction potential of a species, *X*. An analogous cycle applies for oxidation.

Results

A comparison of the calculated and experimental values for the redox potentials of each compound is shown in Table S3. The calculated values consistently under-estimate the experimental redox potentials. We have applied these values to a thermodynamic analysis of PET.

Table S3 Calculated and measured oxidation and reduction potentials of the measured compounds in acetonitrile.

Compound	Process	Calculated		Experimental
		ΔG° / kJ mol^{-1}	E° / V (*vs.* Fc)	E°/ V (*vs.* Fc)
Cy3.5	Oxidation	–503	+0.38	+0.56
	Reduction	–311	–1.60	–1.47
Atto590	Oxidation	–498	+0.33	+0.95
	Reduction	–327	–1.44	–1.34
**SO** closed	Oxidation	–476	+0.11	+0.35
	Reduction	–231	–2.44	not observed
**SO** open	Oxidation	–472	+0.06	-[a]
	Reduction	–315	–1.56	-[a]

[a] We cannot measure the oxidation and reduction potentials of the spironaphthoxazine in its open (merocyanine) form as it does not persist in solution.

The ΔG_{PET} of electron transfer for all possible scenarios was calculated using the Weller equation (Equation S7),[9] in which $E(\mathrm{D/D^{+}})$ and $\Delta E(\mathrm{D})$ are the oxidation potential of the donor and singlet excitation energy of the fluorophore, respectively, and $E(\mathrm{A^{-}/A})$ is the reduction potential of the acceptor. The final term ($e^2/\varepsilon r$) accounts for the free energy gained by bringing the ions together in solution, however it is smaller than the errors in the redox potential measurements, so it can be ignored for this calculation. In this analysis, excited state energies of 2.09 and 2.07 eV for

Atto590 and Cy3.5, respectively, were calculated as the midpoint between the absorption and emission maxima. ΔG_{PET} of the possible photoinduced electron transfer processes are shown in Table S4.

$$\Delta G_{\mathrm{PET}} = E(\mathrm{D}/\mathrm{D}^{+}) - \Delta E(\mathrm{D}) - E(\mathrm{A}^{-}/\mathrm{A}) - \frac{e^2}{\varepsilon r} \qquad \text{(Eq. S7)}$$

Table S4 Calculated ΔG_{PET} values for different donor and acceptor pairs, compared to experimental results where possible.

Donor	Acceptor	Calculated ΔG_{PET} / eV	Experimental ΔG_{PET} / eV
**SO** closed	Cy3.5	–0.36	–0.29
**SO** open	Cy3.5	–0.41	-[a]
Cy3.5	**SO** closed	+0.75	-[b]
Cy3.5	**SO** open	–0.12	-[a]
**SO** closed	Atto590	–0.55	–0.43
**SO** open	Atto590	–0.59	-[a]
Atto590	**SO** closed	+0.68	-[b]
Atto590	**SO** open	–0.19	-[a]

[a] no experimental result as the redox potentials of **SO** open cannot be measured experimentally. [b] no reduction of **SO** closed was observed experimentally.

As might be expected for the relatively electron rich photoswitch and electron poor dyes, electron transfer is more favorable when the switch acts as the donor in both dye combinations. The negative ΔG_{PET} values in these scenarios imply that some PET quenching of fluorescence is possible in the dyads, both when the spironaphthoxazine is closed and open. Such a process may account for the decrease in quantum yield observed when the dyes are conjugated to the switch (Table S1). In particular, the Atto590 dyads (**3**–**5**), which have shorter linkers than the cyanine dyads (**1** and **2**), show a larger reduction in fluorescence upon conjugation to the switch. This could be due to the highly distant-dependent nature of electron transfer, which becomes very unfavorable as the donor-acceptor distance increases. Indeed, the fluorescence quantum yield variation between dyads **3**–**5**, where the linkers have different flexibility, may be accounted for by changes in the electron transfer process from the closed spironaphthoxazine to the Atto590 fluorophore.

Although electron transfer is thermodynamically favorable from both the open and closed spironaphthoxazine to the fluorophores, the difference in ΔG_{PET} is probably not large enough to account for the magnitude of the observed switching in the dyads. It is likely that FRET is the main mechanism by which the open spironaphthoxazine quenches the fluorophores, but PET may contribute to the quenching.

6. Vesicle Preparation

Preparation of GUVs for imaging

GUVs were freshly prepared on the same day as imaging, according to the following electroformation procedure:[10] The electroformation chamber was cleaned with ethanol and dried thoroughly under N_2 flow. A solution of DOPC (5 µL of a 1 mg mL^{-1} solution in $CHCl_3$) was spread evenly over two Pt electrodes and dried under N_2 flow. A solution of sucrose (370 µL, 300 mM aqueous) was placed in the electroformation chamber and the Pt electrodes were inserted into this solution. Using a function generator (frequency 10 Hz, amplitude 5.7 $V_{\text{peak-to-peak}}$ = 2 V_{RMS}) an electrical potential was applied to the sample for 1 h to form the GUVs. After 1 h, the frequency was reduced to 2 Hz and left for a further 30 min to detach the GUVs from the electrodes. For confocal and RESOLFT imaging, part the GUV solution (75 µL) was transferred into an Eppendorf tube and a solution of dyad (1 µL, 1 mM in DMSO) was added and left for 15 min. For FCS measurements, a solution of the dyad (0.5 µL, 1 µM in DMSO) was added to the vesicle solution (50 µL). The vesicle solution was then placed into PBS (250 µL) in the well of an 8-well ibidi plate for imaging. All transfers of GUVs were carried out using trimmed pipette tips.

Phosphate buffered saline was made by dissolution of tablets (ThermoFisher) in deionized water. The concentrations of the components are as follows: NaCl at 8.0 g L^{-1}, KCl at 0.2 g L^{-1}, Na_2HPO_4 at 1.15 g L^{-1}, KH_2PO_4 at 0.2 g L^{-1}.

Preparation of SLBs for Zeiss confocal imaging

PBS solution (100 µL) containing GUVs was taken from the 8-well plate and deposited on a plasma-cleaned glass slide which induces the vesicles to collapse and form supported lipid bilayers on the glass surface. A further 500 µL of PBS was added to the imaging chamber to hydrate the bilayers.

7. Microscopy

RESOLFT microscopy

RESOLFT microscopy was performed on a modified Abberior Instruments RESOLFT microscope (Abberior Instruments, Göttingen, Germany), equipped with excitation lasers at 594 nm (pulsed 80 ps at 80 MHz, LightUp594, Abberior Instruments) and 640 nm (pulsed (80 ps at 80 MHz) or CW, LDH-D-C-640P, Picoquant), and customized to provide a 405 nm doughnut shaped laser via a vortex phase mask (VPP-1b, RPC Photonics, Rochester, NY), placed in the path of the 405 nm source (Cobolt diode laser, CW) Shuttering of the lasers was achieved with acousto-optical modulators (MT110-A1.5-VIS, Photon Lines, Banbury, UK). Images were acquired with 100×/1.4 NA oil immersion objective lens (UPlanSApo 100×/1.4 oil, Olympus, Japan) and detected with photon counting avalanche photo diodes (restricted to detection range either 605 to 625 nm or 650 to 755 nm). The microscope was operated with Imspector software (Abberior Instruments, Göttingen, Germany) and image analysis was carried out in Imspector and FIJI ImageJ. Lookup tables for the images are based on min/max pixel intensities for each image, with no correction for background. Zero on the lookup table corresponds to a zero photon count. For a schematic diagram of the beam paths, see Fig. S30.

Laser powers, P, were measured at the back focal plane of the objective. Together with the FWHM of the focal laser intensity distribution, they allow for the calculation of the time-averaged intensity $I = P / [\pi(\mathrm{FWHM}/2)^2]$ and a pulse peak intensity $I_{peak} = I/(\tau f)$ with pulse width τ = 80 ps and repetition rate f = 80 MHz. The FWHM of the laser spot can be calibrated from images of 100 nm TetraSpeck beads taken with each laser, and are approximately 205 nm for the 405 nm laser, 225 nm for the 594 nm laser, and 235 nm for the 640 nm laser. These measurements give intensities for each laser as follows: 405 nm (P = 80 – 355 µW corresponds to I = 0.24 – 1.08 MW cm^{-2}); 594 nm (P = 2.0 – 5.0 µW corresponds to I = 5.0 – 12.6 kW cm^{-2}); 640 nm (P = 3.6 – 20 µW corresponds to I = 8.3 – 46 kW cm^{-2}).

For imaging on the Abberior RESOLFT microscope, glass ibidi µ-Slide 8-well plates were passivated with poly-L-lysine for 45 min, before washing with PBS three times, leaving 250 µL in the chamber. Then, 75 µL of the GUV solution was transferred into the wells.

In order to acquire RESOLFT images, we used a pixel-by-pixel imaging sequence where each pixel is irradiated with a number of laser beams sequentially. Each pixel was irradiated as follows: firstly, a Gaussian-shaped 594 nm excitation spot acquired the confocal signal; secondly, a donut-shaped 405 nm pulse converted the spironaphthoxazine to its active open form; thirdly, the Gaussian-shaped 594 nm excitation spot acquired the RESOLFT signal by inducing fluorescence from the central (unquenched) region of the pixel. Each laser was applied sequentially to each pixel in the image, and raster scanning was used to build up the image. Each RESOLFT image is accompanied by a confocal image acquired simultaneously which acts as an internal control. Excitation (594 nm) powers were set such that the detector was not saturated. Because of the nature of super resolution imaging, it was necessary to have small pixel sizes (40 × 40 nm here) to allow sufficient sampling. The sample area irradiated by a laser beam is much bigger than the size of the pixels. In addition to the pixel at the center of the laser beam, surrounding pixels are also irradiated, switching neighboring molecules into their dark states. This manifests itself in subsequent pixels appearing much darker than they should in the bright images, because molecules are still in their dark states following irradiation of earlier pixels.

In our initial experiments we introduced a waiting time to allow the molecules in neighboring pixels to recover before moving to the next pixel. This allowed us to acquire our first images with sub-diffraction limit resolution (see Figure S16). However, we found that several hundreds of milliseconds of waiting time were necessary for full recovery of the molecules, making the imaging very slow (33 minutes for a 2 × 2 µm field of view).

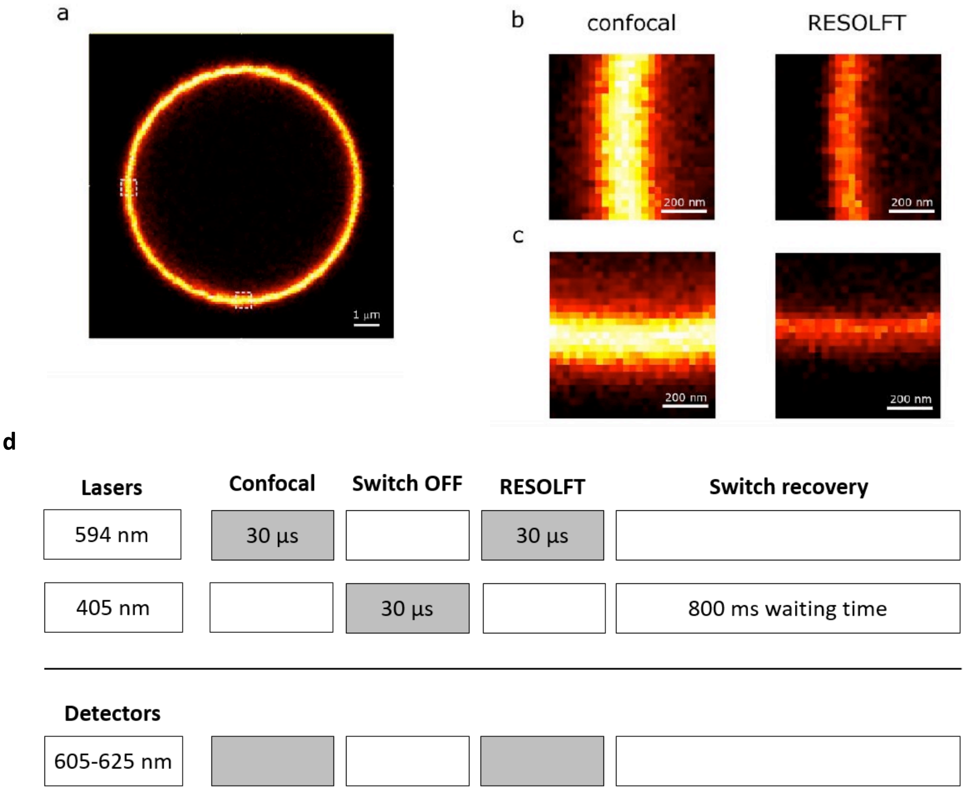

Figure S16 RESOLFT images of GUVs stained with an Atto565 analogue of dyad **3** (Dyad **2** in Ref:[6]): (a) confocal image of a GUV; (b) and (c) confocal and RESOLFT images of sections of the GUV membrane highlighted in (a); (d) pixel-by-pixel sequence used to acquire the image; λ_{ex} 594 nm, 4.4 µW power, $\lambda_{switch\text{-}off}$ 405 nm, 120 µW power, FWHM (confocal) = 300 nm, FWHM (RESOLFT)) = 185 nm.

As an alternative solution, we introduced a fourth laser pulse to photochemically accelerate the ring closing reaction of the open merocyanine to the closed spiro form. We tested lasers at three available wavelengths and found that 640 nm was the most suitable. This allowed significantly faster image acquisition. The pixel-by-pixel imaging sequence is shown in Figure 17.

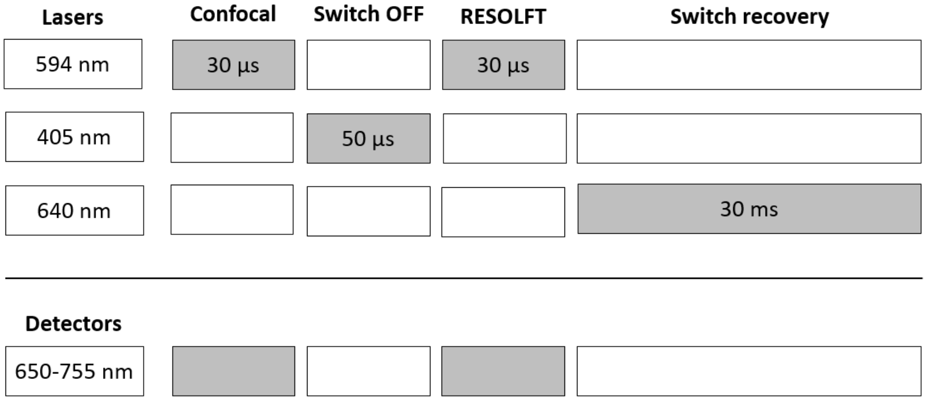

Figure S17 Example imaging sequence for pixel-by-pixel RESOLFT microscopy using 3 lasers: 594 nm for excitation of the fluorophore; 405 nm for photochemical ring opening of the spironaphthoxazine; 640 nm for photochemical acceleration of the thermal ring closing of the merocyanine form of the switch.

Before attempting super-resolution imaging using this setup, we explored the fluorescence switching behavior in pixel-by pixel acquisition by confocal imaging. Confocal imaging of the GUVs clearly showed that the dyad was incorporated into the lipid bilayer and was not present in significant concentration in the aqueous solution either inside or outside the vesicle. Switching off of the dyad was achieved using a Gaussian-shaped 405 nm beam rather than a donut-shaped beam. We varied the dwell times and laser powers to find the optimal conditions for switching

(see S18–S21). We observed that optimal switching for dyads **1** and **2** was achieved using 400–500 µW of the 405 nm laser, but for dyads **3**–**5** lower 405 nm laser powers were required. For dyad **1**, increasing the power beyond 500 µW did not seem to lead to poorer quenching, whereas for the other dyads, high laser powers led to a drop in quenching efficiency. For all dyads, higher 405 nm laser powers resulted in faster switching fatigue, as demonstrated later. Having optimized the power of the switch-off laser, we used similar experiments to find optimal parameters for the 640 nm laser.

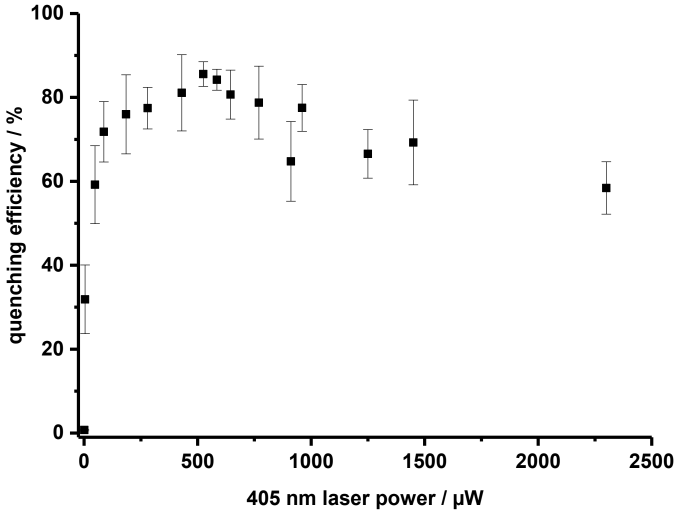

Figure S18 Dependence of fluorescence quenching of dyad **2** on 405 nm laser power; pixel by pixel, λ_{ex} 594 nm, 2.0 µW, $\lambda_{recovery}$ 640 nm, 20 µW (continuous wave), pixel size 40 nm, 5 repeats.

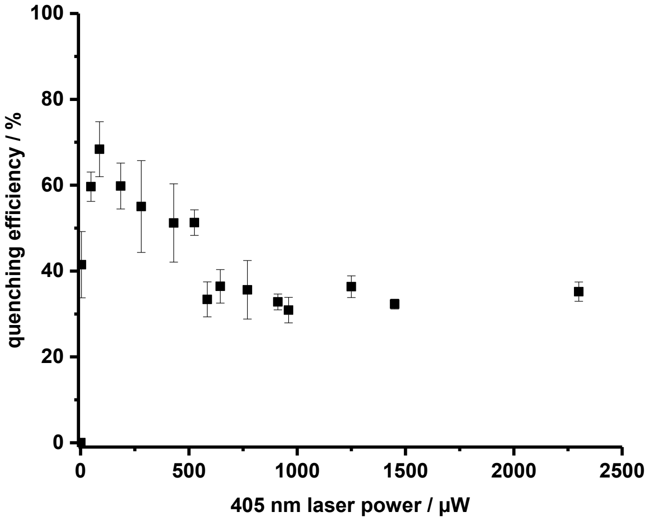

Figure S19 Dependence of fluorescence quenching of dyad **3** on 405 nm laser power; pixel by pixel, λ_{ex} 594 nm, 2.0 µW, $\lambda_{recovery}$ 640 nm, 3.6 µW (pulsed), pixel size 40 nm, 4 repeats.

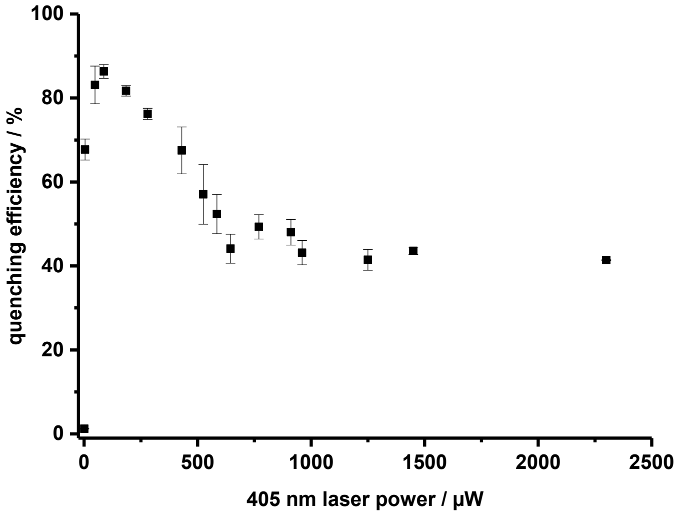

Figure S20 Dependence of fluorescence quenching of dyad **4** on 405 nm laser power; pixel by pixel, λ_{ex} 594 nm, 2.0 µW, $\lambda_{recovery}$ 640 nm, 3.6 µW (pulsed), pixel size 40 nm, 4 repeats.

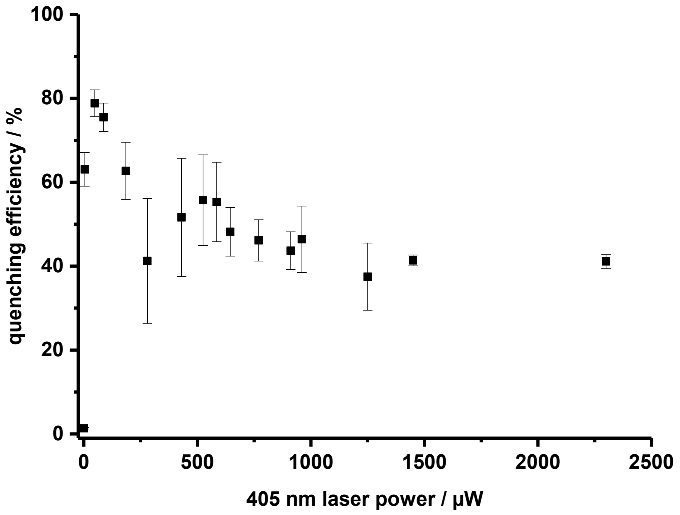

Figure S21 Dependence of fluorescence quenching of dyad **5** on 405 nm laser power; pixel by pixel, λ_{ex} 594 nm, 2.0 µW, $\lambda_{recovery}$ 640 nm, 3.6 µW (pulsed), pixel size 40 nm, 4 repeats.

Addition of a phase plate in the path of the 405 nm laser produced a donut-shaped beam with zero intensity at the center. With this in place, we observed resolution enhancement in our images (Figure 4 and Figures S24–28), with around a 2-fold improvement in FWHM for all dyads (see Table S5 and Figure S29). In some of our images, we observed that the resolution enhancement was good in the top rows of pixels of the image (rows acquired early in the raster scan) but deteriorated towards the bottom of the image. This is probably due to the diffusion of the dyads through the membrane. In the time that it takes to record the image, the molecules can diffuse through the membrane such that as we reach the bottom of the image, we are irradiating photoswitches that have already suffered fatigue.

Analysis of Microscopy Line Profiles

The RESOLFT microscopy experiments on DOPC GUVs stained with dyads **1**–**5** resulted in the intensity line profiles, perpendicular to the membrane at the equatorial plane of the vesicles, as shown in Figures 4 and S24–S28. Each experiment simultaneously generated two line profiles: one from the conventional confocal imaging mode and one from the RESOLFT super-resolution imaging mode.

The confocal line profiles are well described by a simple Gaussian curve (Equation S8),

$$I = I_0 + I_p\, e^{\frac{-4(ln2)(x-x_p)^2}{w_{con}^2}} \qquad \text{(Eq. S8)}$$

where I is the signal intensity (photon counts), I_0 is a baseline correction, I_p is the peak intensity, x is the position along the line, x_p is the center of the peak, and w_{con} is the peak width (FWHM, Table S5).

The RESOLFT line profiles are better fitted by a Lorentzian model,[11,12] rather than a single Gaussian (Figs S22 and S23), giving the FWHM values ($w_{SRM\text{-}L}$ in Table S5). In Figures 4 and S24–S28, we have shown Gaussian fits for confocal profiles and Lorentzian fits for RESOLFT profiles, and we quantify the increased resolution by the ratio $w_{con}/w_{SRM\text{-}L}$ (see also values in Table S5 and Figure S29).

The RESOLFT line profiles are also well described by a double Gaussian model[13] (Equation S9),

$$I = I_0 + I_{con}\, e^{\frac{-4(ln2)(x-x_p)^2}{w_{con}^2}} + I_{SRM}\, e^{\frac{-4(ln2)(x-x_p)^2}{w_{SRM\text{-}2G}^2}} \qquad \text{(Eq. S9)}$$

where w_{con} is fixed as the peak width from the corresponding confocal profile and $w_{SRM\text{-}2G}$ is the fitted width of the RESOLFT component (parameters summarized in Table S5). The $w_{SRM\text{-}2G}$ values extracted from these fits are smaller than the corresponding $w_{SRM\text{-}L}$ values obtained from Lorentzian fits, suggesting that the unsuppressed confocal contribution limits the resolution enhancement we can achieve. The observed enhancement in resolution ($w_{con}/w_{SRM\text{-}2G}$) also increases (see Table S5).

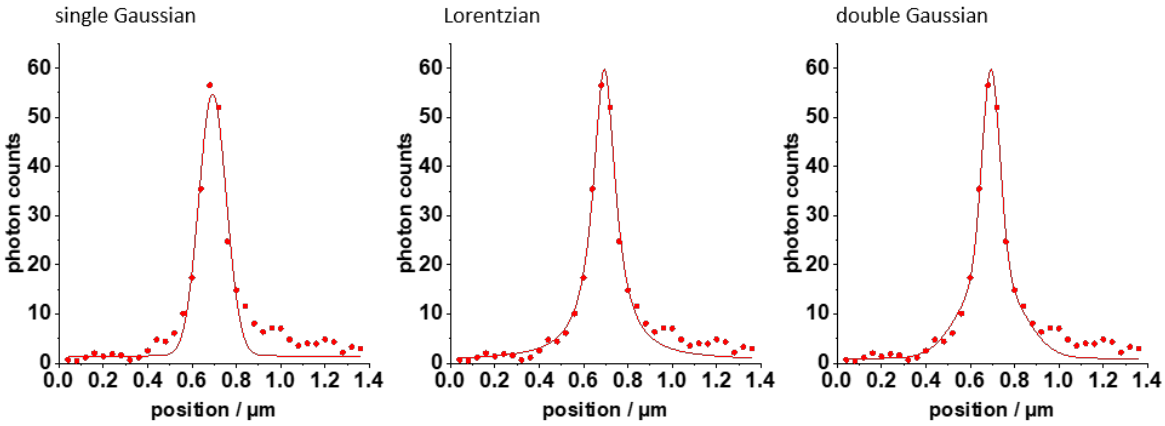

Figure S22 RESOLFT line profile duplicated from Figure 4d (dyad **1**), fitted with a single Gaussian (left, R^2 = 0.944), a Lorentzian (center, R^2 = 0.981), or a double Gaussian model of the form in Equation S9 (right, R^2 = 0.987).

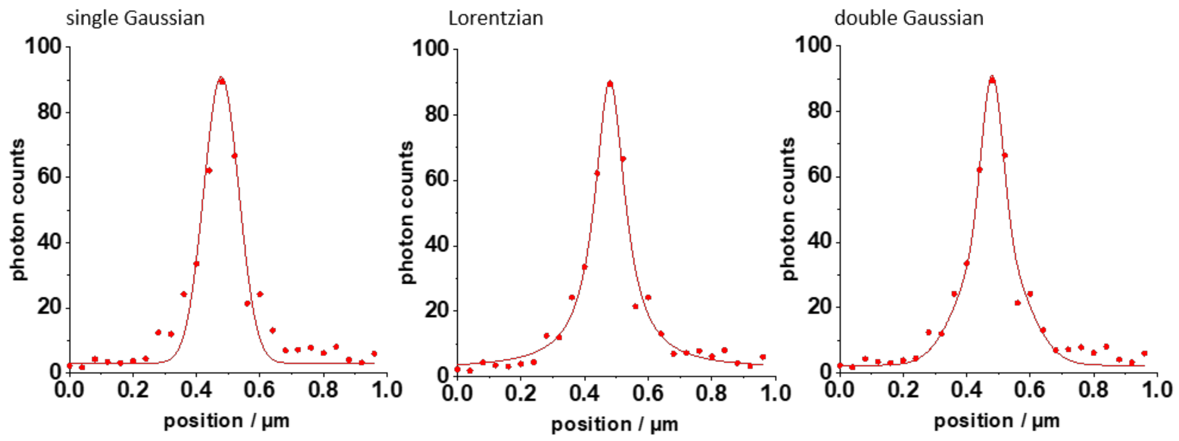

Figure S23 RESOLFT line profile duplicated from Figure 4f (dyad **3**), fitted with a single Gaussian (left, R^2 = 0.929), a Lorentzian (center, R^2 = 0.979), or a double Gaussian model of the form in Equation S9 (right, R^2 = 0.975).

Table S5 Analysis of microscopy line profiles

Dyad	Figure	Confocal FWHM (Gaussian) w_{con} / nm	RESOLFT FWHM (Lorentzian) $w_{SRM\text{-}L}$ / nm	ratio $w_{con}/w_{SRM\text{-}L}$	RESOLFT FWHM (double Gaussian) $w_{SRM\text{-}2G}$ / nm	ratio $w_{con}/w_{SRM\text{-}2G}$	$I_{SRM}/(I_{SRM} + I_{con})$
**1**	4d	337	124	2.7	98	3.2	0.76
**1**	S24b	272	144	1.9	97	2.8	0.62
**1**	S24d	337	138	2.4	105	3.2	0.71
**2**	S25b	320	135	2.4	99	3.2	0.66
**2**	S25d	297	131	2.3	105	2.8	0.70
**3**	4f	227	116	2.0	81	2.8	0.62
**3**	S26b	243	117	2.1	92	2.6	0.70
**3**	S26d	248	138	2.0	92	2.7	0.66
**4**	S27b	238	110	2.1	90	2.6	0.70
**4**	S27d	212	107	2.0	106	2.0	0.83
**5**	S28b	255	153	1.7	100	2.5	0.57
**5**	S28d	249	137	1.8	79	3.1	0.53

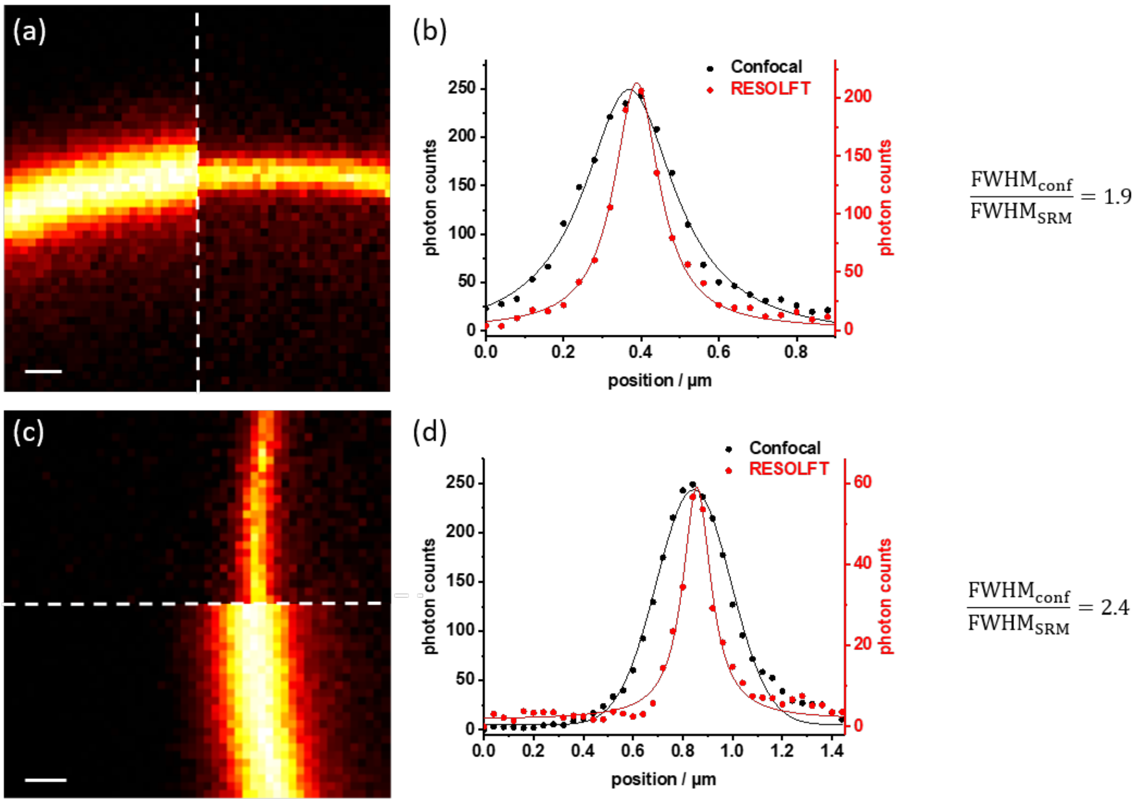

Figure S24 RESOLFT images of DOPC GUV membranes stained with dyad **1**: (a) and (c) confocal and RESOLFT comparison of a section of a GUV membrane, taken in the equatorial plane; (b) and (d) corresponding radial line profiles showing fits used to find FWHM values (Gaussian for confocal, Lorentzian for RESOLFT); λ_{ex} 594 nm, 2.0 µW, 30 µs; $\lambda_{switch\text{-}off}$ 405 nm 155 µW, 20 µs (image a), 335 µW, 50 µs (image c); $\lambda_{recovery}$ 640 nm, 20 µW (continuous wave), 30 ms; scale bar = 200 nm.

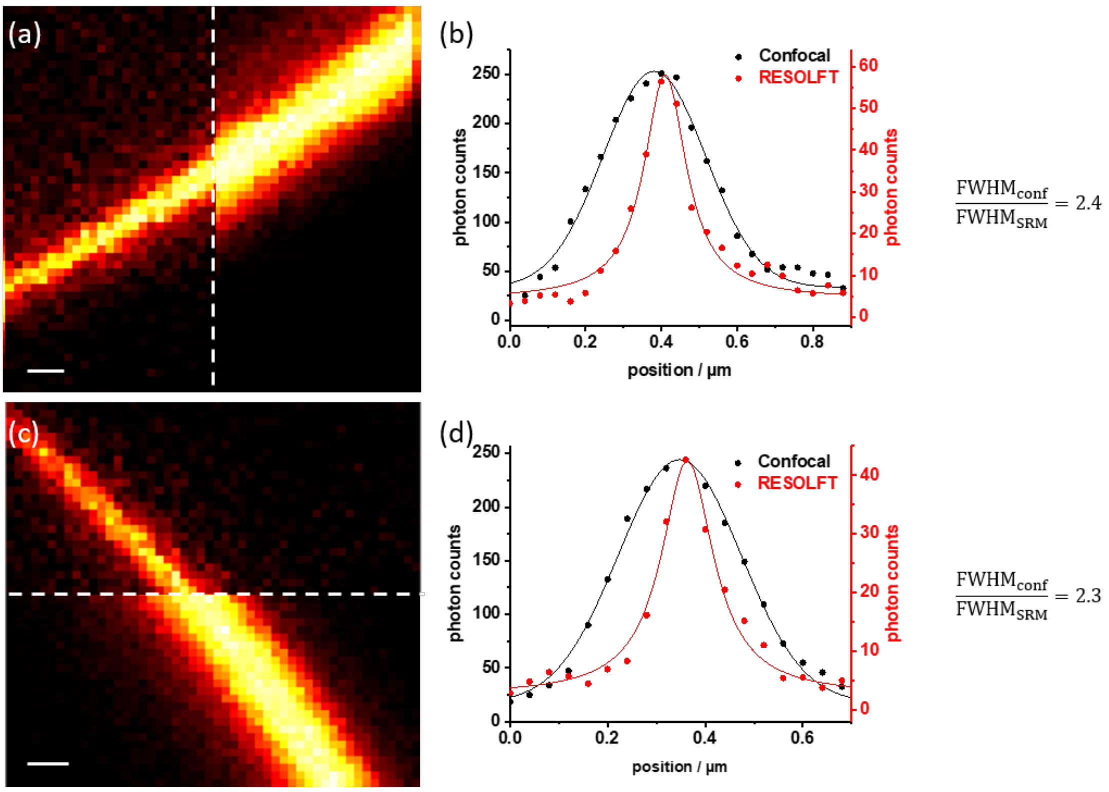

Figure S25 RESOLFT images of DOPC GUV membranes stained with dyad **2**: (a) and (c) confocal and RESOLFT comparison of a section of a GUV membrane, taken in the equatorial plane; (b) and (d) corresponding radial line profiles showing fits used to find FWHM values (Gaussian for confocal, Lorentzian for RESOLFT); λ_{ex} 594 nm, 2.0 µW, 30 µs; $\lambda_{switch\text{-}off}$ 405 nm 498 µW (image a) 335 µW (image c), 50 µs; $\lambda_{recovery}$ 640 nm, 20 µW (continuous wave), 30 ms; scale bar = 200 nm.

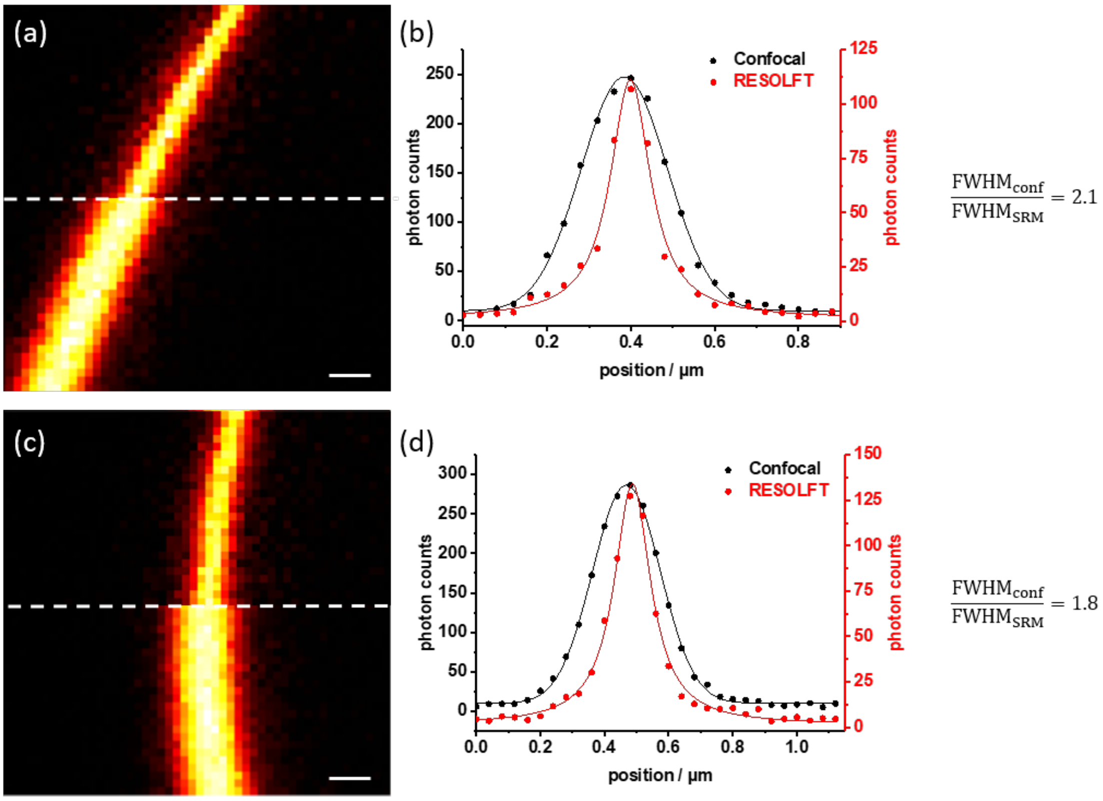

Figure S26 RESOLFT images of DOPC GUV membranes stained with dyad **3**: (a) and (c) confocal and RESOLFT comparison of a section of a GUV membrane, taken in the equatorial plane; (b) and (d) corresponding radial line profiles showing fits used to find FWHM values (Gaussian for confocal, Lorentzian for RESOLFT); λ_{ex} 594 nm, 2.0 µW, 30 µs; $\lambda_{switch\text{-}off}$ 405 nm 155 µW (image a), 80 µW (image c), 50 µs; $\lambda_{recovery}$ 640 nm, 3.6 µW (pulsed), 30 ms; scale bar = 200 nm.

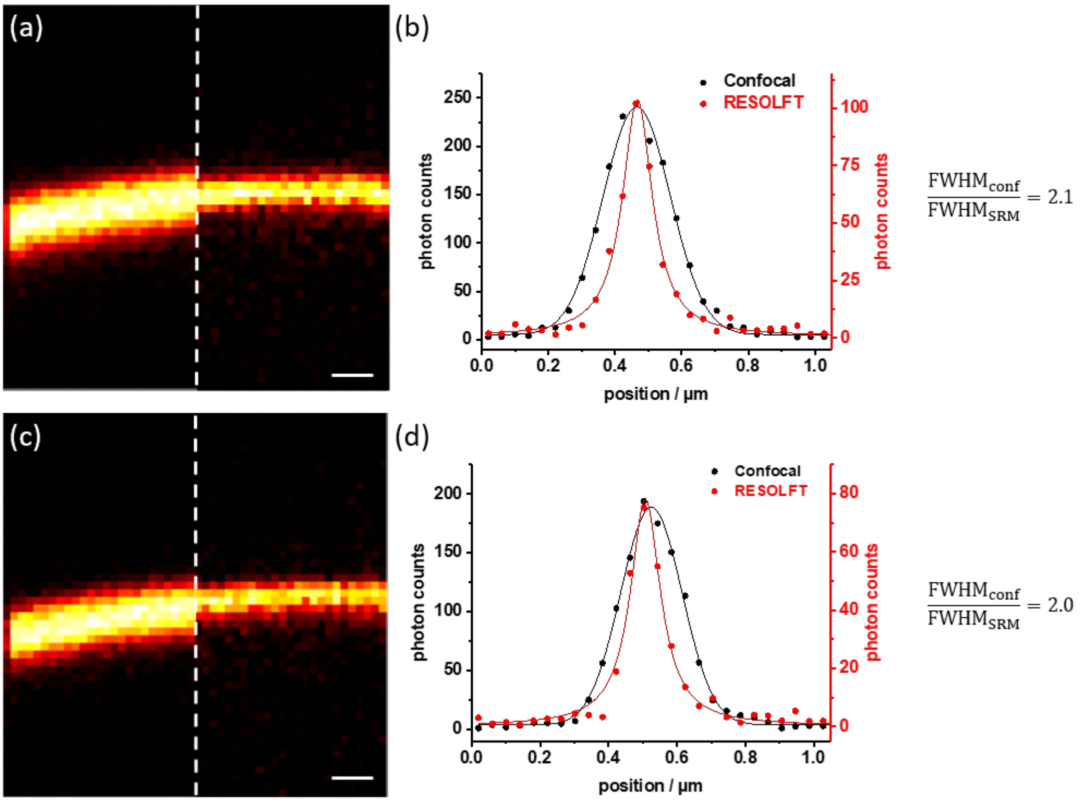

Figure S27 RESOLFT images of DOPC GUV membranes stained with dyad **4**: (a) and (c) confocal and RESOLFT comparison of a section of a GUV membrane, taken in the equatorial plane; (b) and (d) corresponding radial line profiles showing fits used to find FWHM values (Gaussian for confocal, Lorentzian for RESOLFT); λ_{ex} 594 nm, 2.0 µW, 30 µs; $\lambda_{switch\text{-}off}$ 405 nm, 80 µW, 50 µs; $\lambda_{recovery}$ 640 nm, 3.6 µW (pulsed), 30 ms; scale bar = 200 nm.

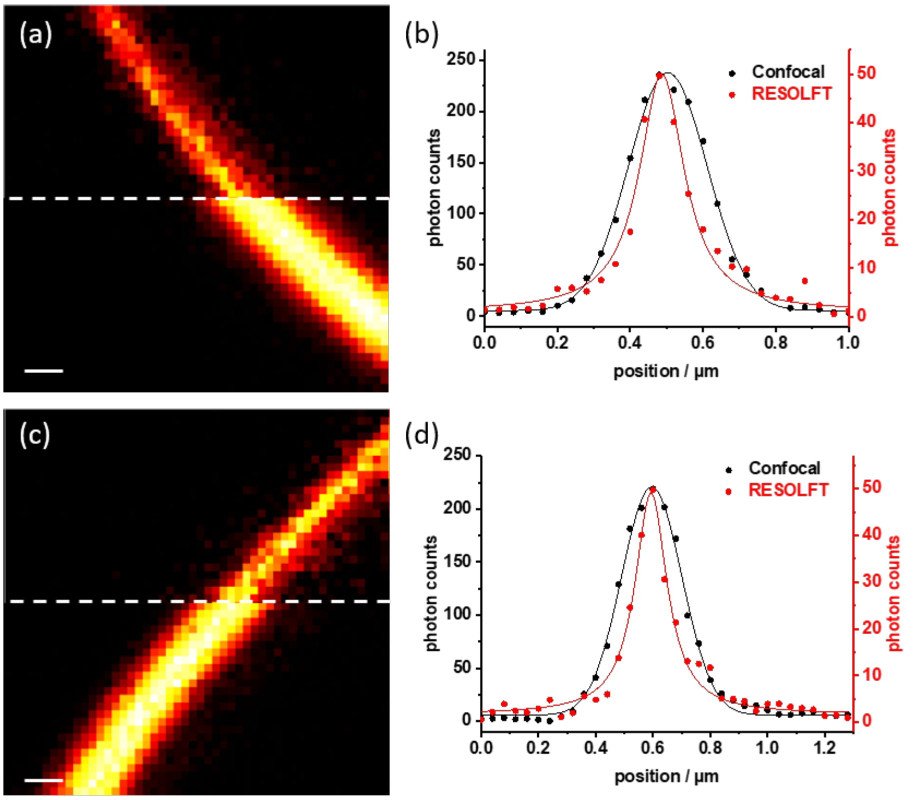

$$\frac{\mathrm{FWHM_{conf}}}{\mathrm{FWHM_{SRM}}} = 1.7$$

$$\frac{\mathrm{FWHM_{conf}}}{\mathrm{FWHM_{SRM}}} = 1.8$$

Figure S28 RESOLFT images of DOPC GUV membranes stained with dyad **5**: (a) and (c) confocal and RESOLFT comparison of a section of a GUV membrane, taken in the equatorial plane; (b) and (d) corresponding radial line profiles showing fits used to find FWHM values (Gaussian for confocal, Lorentzian for RESOLFT); λ_{ex} 594 nm, 2.0 µW, 30 µs; $\lambda_{switch\text{-}off}$ 405 nm, 155 µW, 50 µs; $\lambda_{recovery}$ 640 nm, 3.6 µW (pulsed), 30 ms; scale bar = 200 nm.

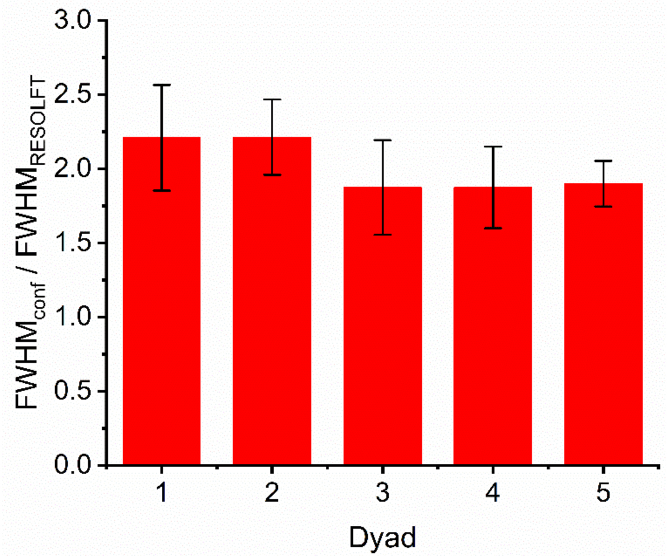

Figure S29 Average ratios of FWHM values obtained from the line profiles drawn in confocal and RESOLFT microscopy images of GUVs stained with dyads **1**–**5**. The FWHM values used here are from Gaussian confocal and Lorentzian RESOLFT fits. Error bars represent standard deviations. Number of images analyzed for each dyad: dyad **1**, 27; dyad **2**, 15; dyad **3**, 20; dyad **4**, 19; dyad **5**, 13.

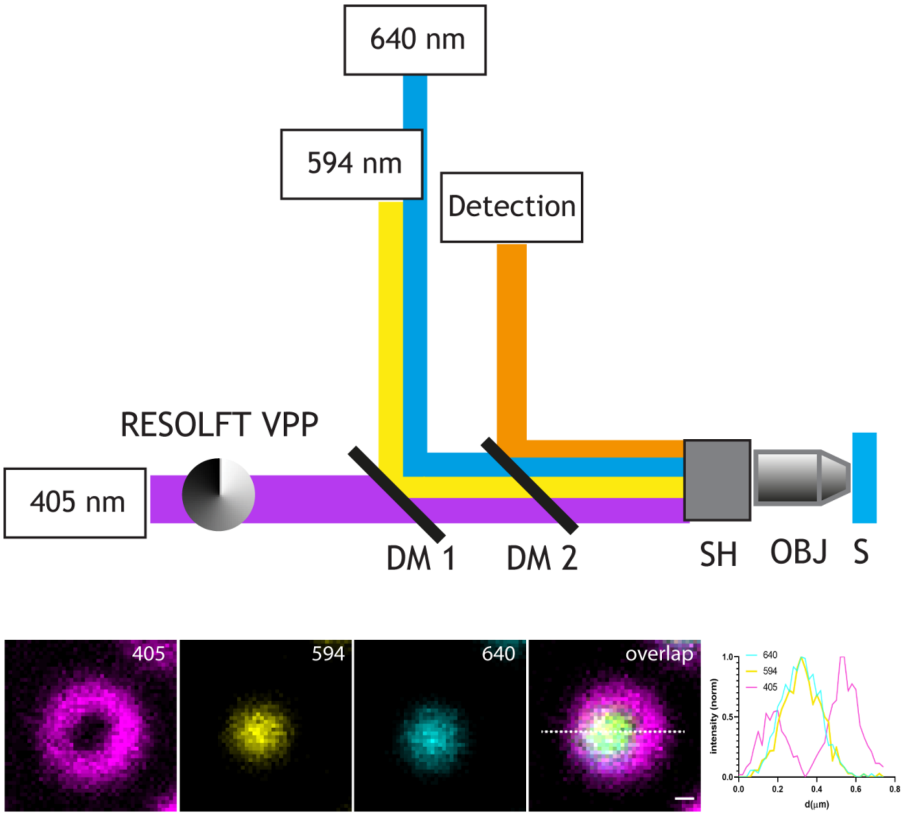

Figure S30 Top: Schematic representation of the beam paths on the RESOLFT microscope (VPP = vortex phase plate, DM = dichroic mirror, SH = scanning head, OBJ = objective, S = sample); Bottom: confocal images of 100 nm TetraSpeck beads excited with 405 nm (phase plate in place), 594 nm and 640 nm, the overlapped image, and the line profiles showing the beam alignments (scale bar = 100 nm).

Confocal microscopy

Confocal images were obtained using a Zeiss LSM780 inverted laser scanning confocal microscope equipped with various excitation sources: 405 nm (diode laser, CW, 815 µW at 100%) and 594 nm (HeNe laser, CW, 84 µW at 100%). Images were acquired using either a Plan Apochromat 63×/1.40 NA DIC M27 oil immersion objective or an LD C-Apochromat 40×/1.1 NA DIC water immersion objective. Unless otherwise stated, images are 512 × 512 pixels, using 80 nm pixels, giving an image size of 42.5 × 42.5 µm. The microscope was operated via a PC running Zen software. Image analysis was carried out in FIJI ImageJ.

For images recorded on the Zeiss LSM780 microscope, the GUV solution was placed in a pre-treated plastic ibiTreat µ-Slide 8-well plate (No. 1.5 coverslip).

All imaging carried out on the Zeiss LSM780 microscope used line-by-line laser switching sequence, rather than the laser sequence changing between pixels (see Figure S31). For example, in the ‘bright image‘, only the 594 nm laser was used to excite the dyads. In the ‘dark image‘, the top line of pixels was first irradiated with the 405 nm light to switch the molecules to their dark state, then this line was scanned again with the 594 nm laser to read out the dark image. This process was repeated for all 512 lines of the image.

It can be seen that in images taken on the Zeiss LSM780 (which has a linearly polarized excitation laser), the vesicles appeared brighter at the top and bottom due to better overlap of the transition dipole moment of the dye with the incident light. This observation confirmed that the dyes were aligned in the tails of the membrane lipids rather than being randomly associated with the surface.

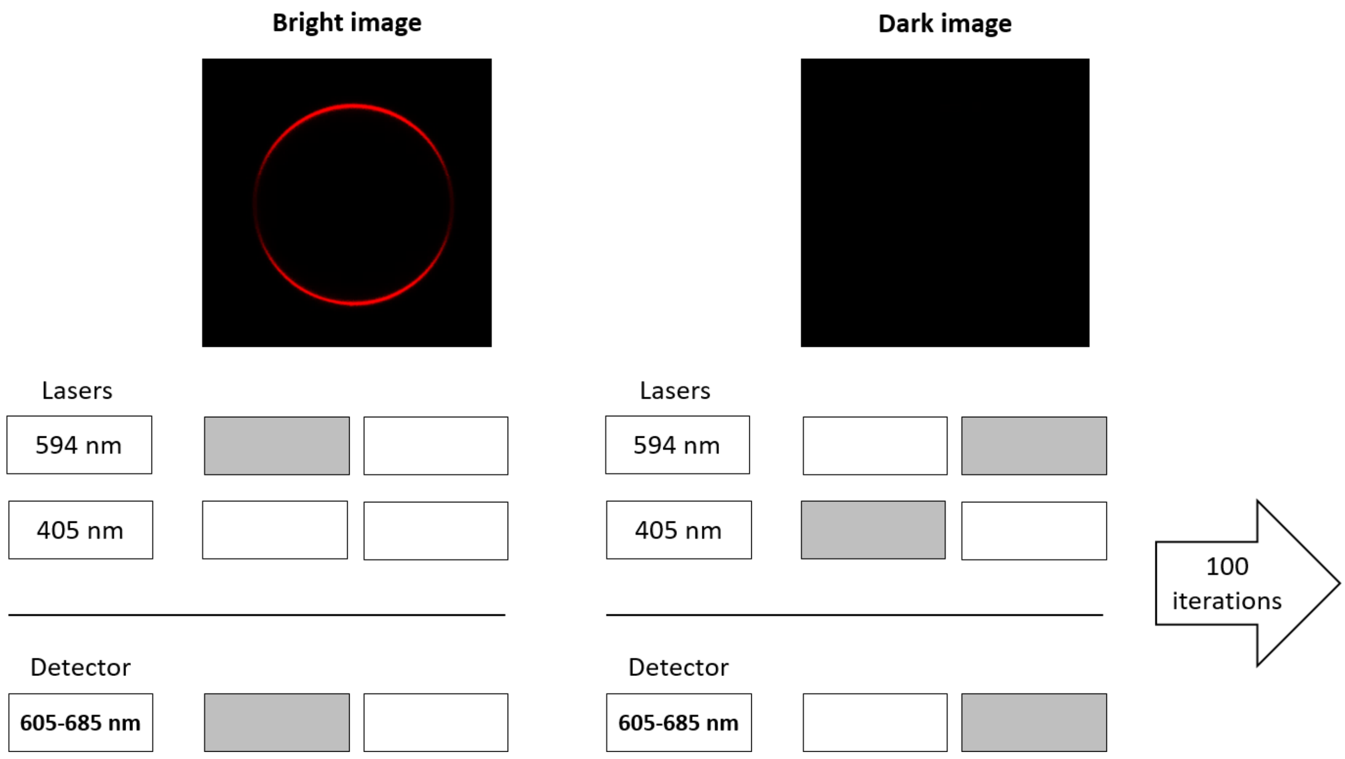

Figure S31 Schematic representation of image acquisition sequence in line-by-line laser switching on the Zeiss LSM780 confocal microscope.

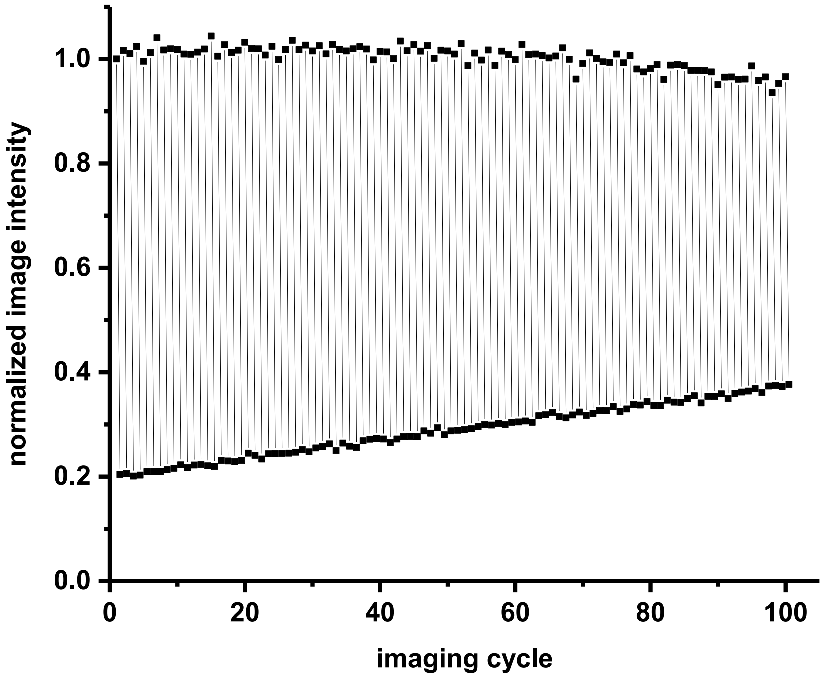

Figure S32 Normalized image intensity of the bright and dark states of dyad **1** over 100 imaging cycles in GUVs (λ_{ex} 594 nm, 0.66 µW power, $\lambda_{switch\text{-}off}$ 405 nm, 15 µW power, pixel dwell time 1.58 µs, pixel size 80 nm).

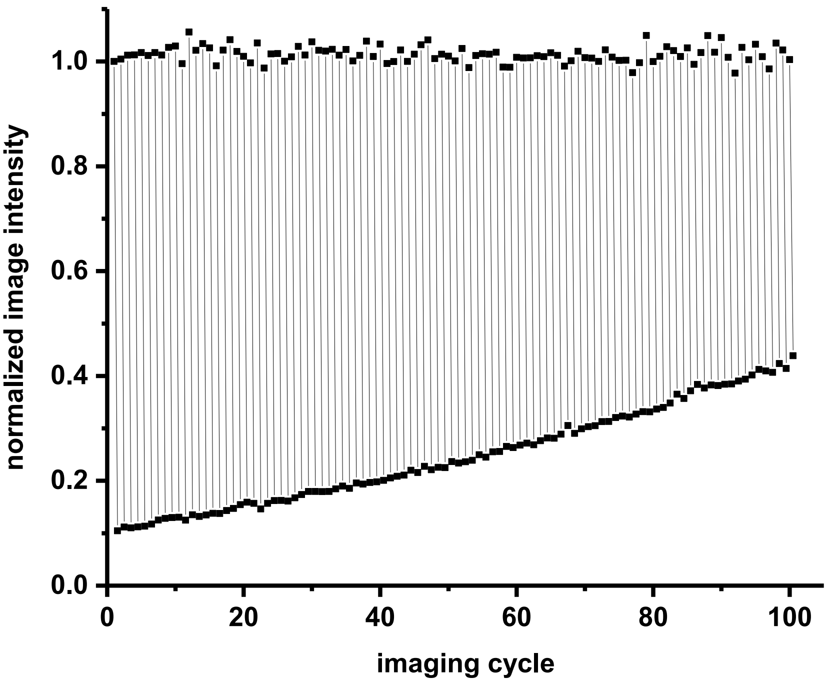

Figure S33 Normalized image intensity of the bright and dark states of dyad **1** over 100 imaging cycles in GUVs (λ_{ex} 594 nm, 0.66 µW power, $\lambda_{switch\text{-}off}$ 405 nm, 32 µW power, pixel dwell time 1.58 µs, pixel size 80 nm).

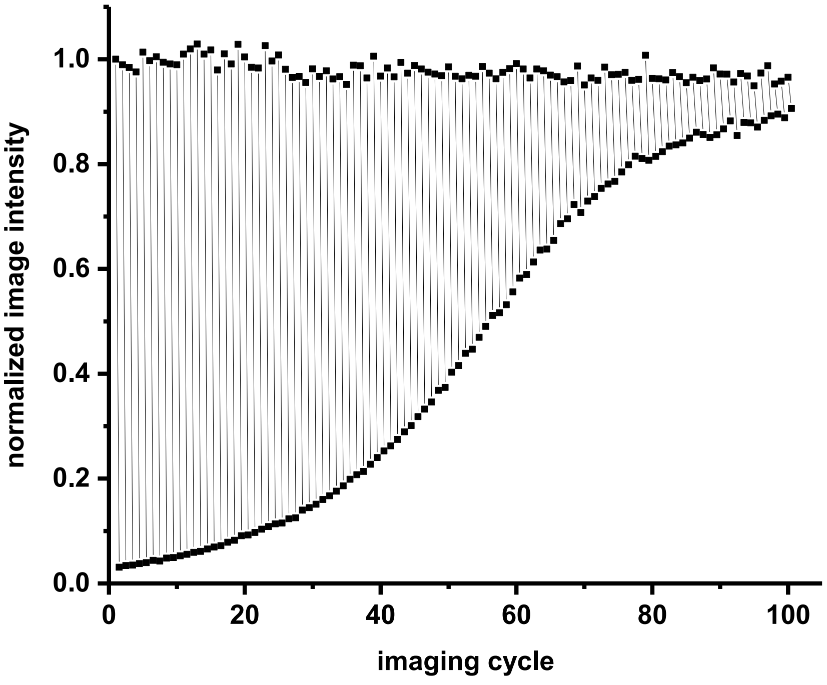

Figure S34 Normalized image intensity of the bright and dark states of dyad **1** over 100 imaging cycles in GUVs (λ_{ex} 594 nm, 0.66 µW power, $\lambda_{switch\text{-}off}$ 405 nm, 85 µW power, pixel dwell time 1.58 µs, pixel size 80 nm).

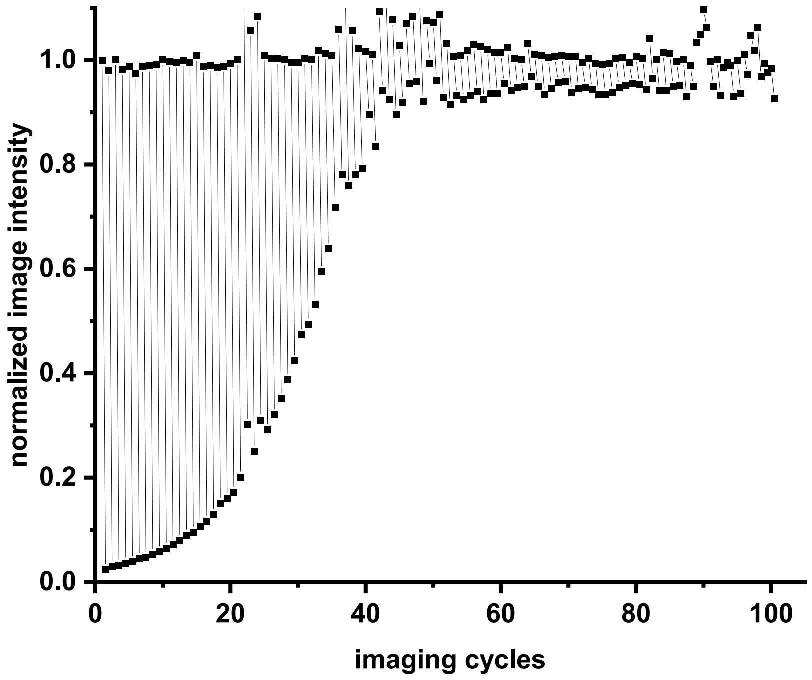

Figure S35 Normalized image intensity of the bright and dark states of dyad **1** over 100 imaging cycles in GUVs (λ_{ex} 594 nm, 0.66 µW power, $\lambda_{switch\text{-}off}$ 405 nm, 171 µW power, pixel dwell time 1.58 µs, pixel size 80 nm).

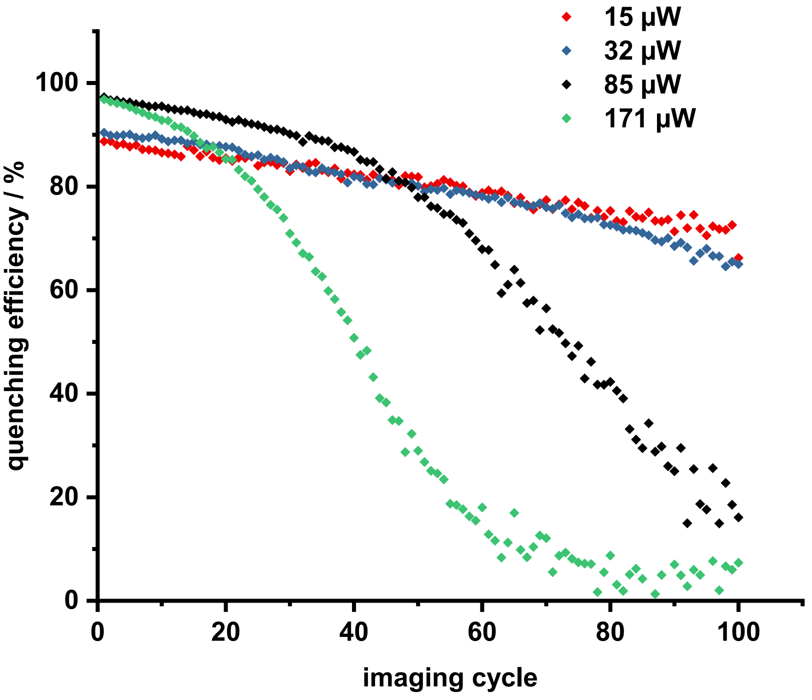

Figure S36 Fatigue of dyad **2** over 100 imaging cycles in GUVs at various 405 nm laser powers (λ_{ex} 594 nm, 0.66 µW power, pixel dwell time 1.58 µs, pixel size 80 nm).

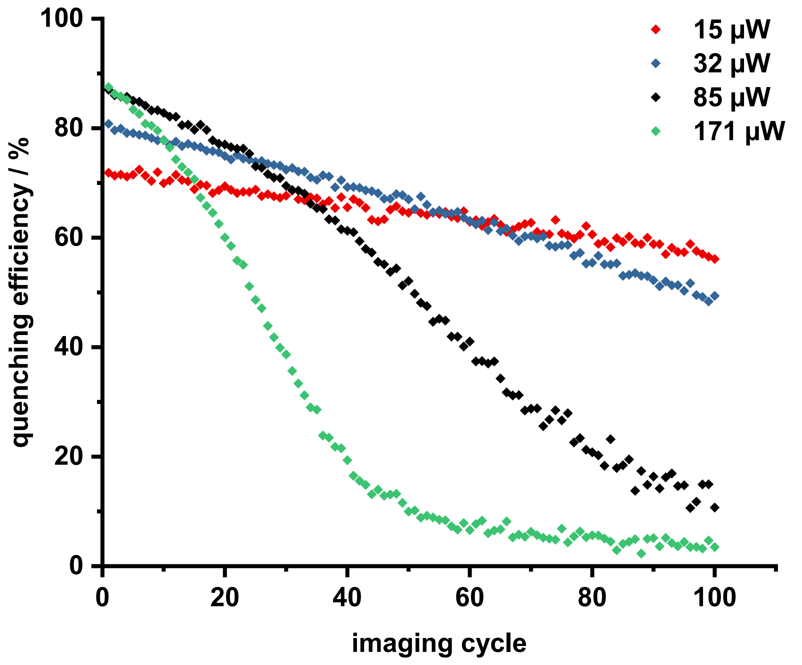

Figure S37 Fatigue of dyad **3** over 100 imaging cycles in GUVs at various 405 nm laser powers (λ_{ex} 594 nm, 0.66 µW power, pixel dwell time 1.58 µs, pixel size 80 nm**).**

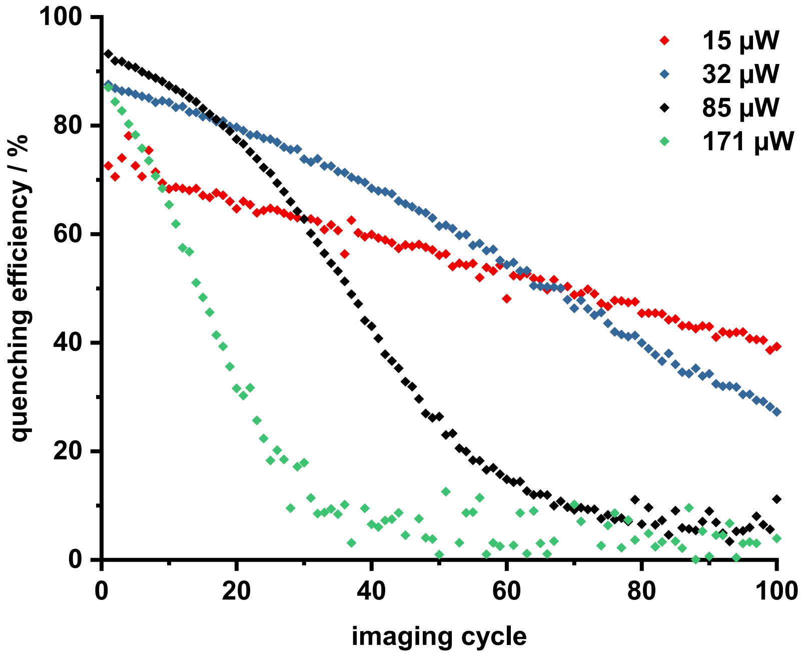

Figure S38 Fatigue of dyad **4** over 100 imaging cycles in GUVs at various 405 nm laser powers (λ_{ex} 594 nm, 0.66 µW power, pixel dwell time 1.58 µs, pixel size 80 nm).

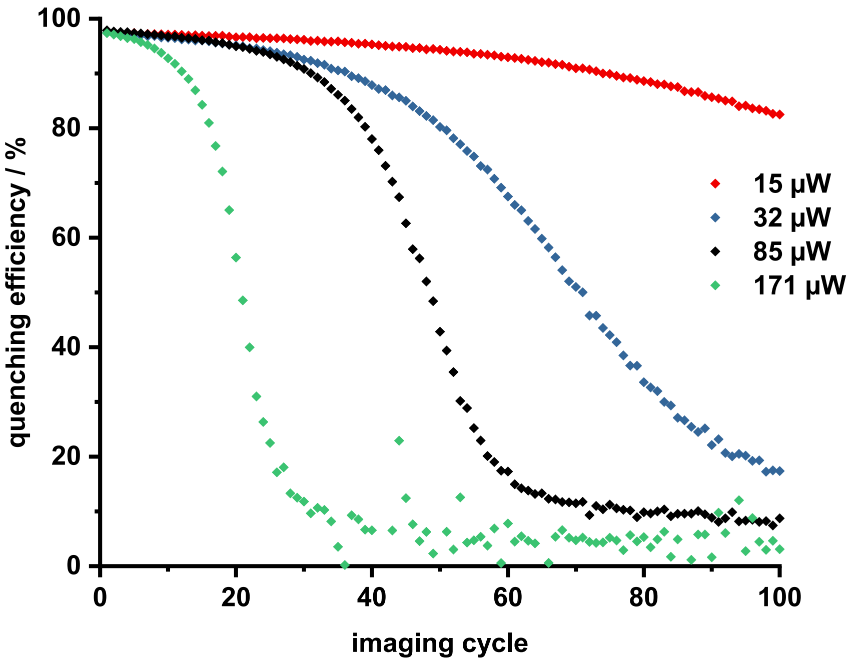

Figure S39 Fatigue of dyad **5** over 100 imaging cycles in GUVs at various 405 nm laser powers (λ_{ex} 594 nm, 0.66 µW power, pixel dwell time 1.58 µs, pixel size 80 nm).

We synthesized a 'dummy dyad' **6** which does not contain a switch. We subjected dye **1a** (containing Cy3.5) and dummy dyad **6** (containing Atto590) to the same 100 imaging cycles experiment as the dyads to see whether there was significant photobleaching of the fluorophore in the experiment (Figure S40). With 0 µW of 405 nm laser power (i.e. only 594 nm excitation), there was no observable photobleaching of either the Atto590 or Cy3.5 dye. At 85 µW of 405 nm laser power there some photobleaching of Cy3.5, and the Atto590 dye exhibits some photobleaching at higher 405 nm laser power. These photobleaching effects are dwarfed by the loss of switching observed for the spironaphthoxazine unit in the dyads.

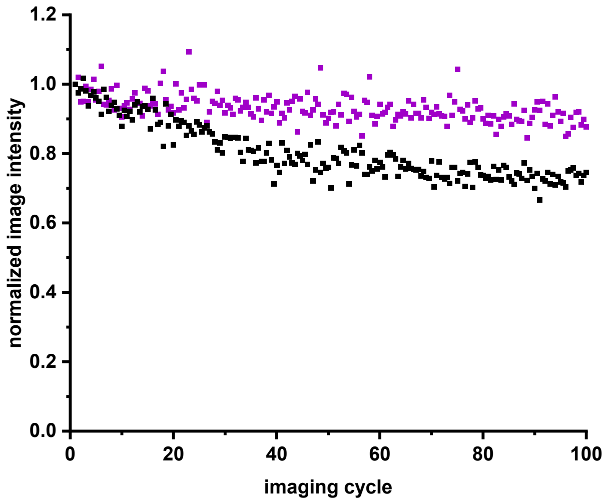

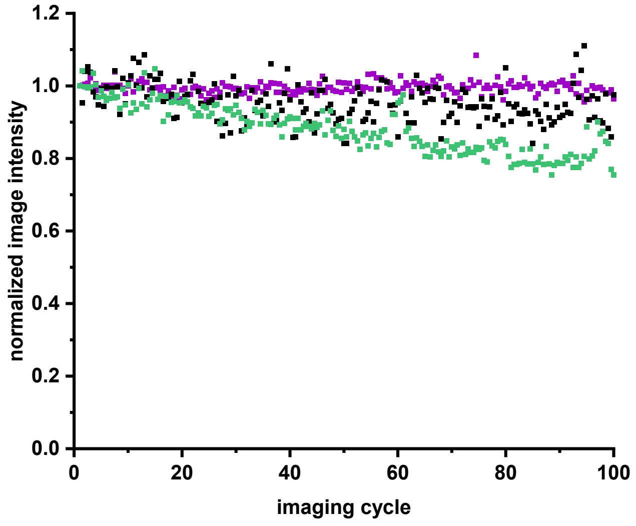

Figure S40 Normalized image intensity of dye **1a** (left) and dummy dyad **6** (right) in a GUVs subjected to 100 switching cycles at various 405 nm laser powers (λ_{ex} 594 nm, 0.66 µW power, $\lambda_{switch\text{-}off}$ 405 nm, 0 µW (purple), 85 µW (black) or 171 µW (green), pixel dwell time 1.58 µs, pixel size 80 nm).

The fatigue curves in Figures S36–S39 are sigmoidal in shape. If simple chemical degradation of the switch occurred, one might expect a first order decay with respect to irradiation time. There are a number of possible explanations for

the sigmoidal shape of the fatigue. One possibility is that the dyad molecules are sufficiently close together that the spironaphthoxazine on one molecule can quench the fluorescent dye of neighboring molecules. Because of the excellent FRET overlap between the fluorescent dye and the active spironaphthoxazine quencher, FRET is efficient over long distances. In this instance, we would expect to see an initial period where we have deterioration of some switches, but neighboring molecules can still efficiently quench the emission, so there is no loss in overall quenching efficiency. Once a critical number of switches has been degraded, the quenching efficiency begins to fall. In a 1:1 mixture of dummy dyad **6** with dyad **3** which has a switch, where there is no quenching of neighboring molecules, one would expect the quenching efficiency to fall significantly (below 50%, as **6** has a higher fluorescence quantum yield than dyad **3**). In fact, our experiments showed that the initial quenching efficiency for a 1:1 mixture was over 80% (Figure S41), confirming that molecules are quenching each other. However, the fatigue resistance of this system was very poor as there is a smaller pool of switches available to quench fluorescence, so the critical number of degraded switches is reached much faster.

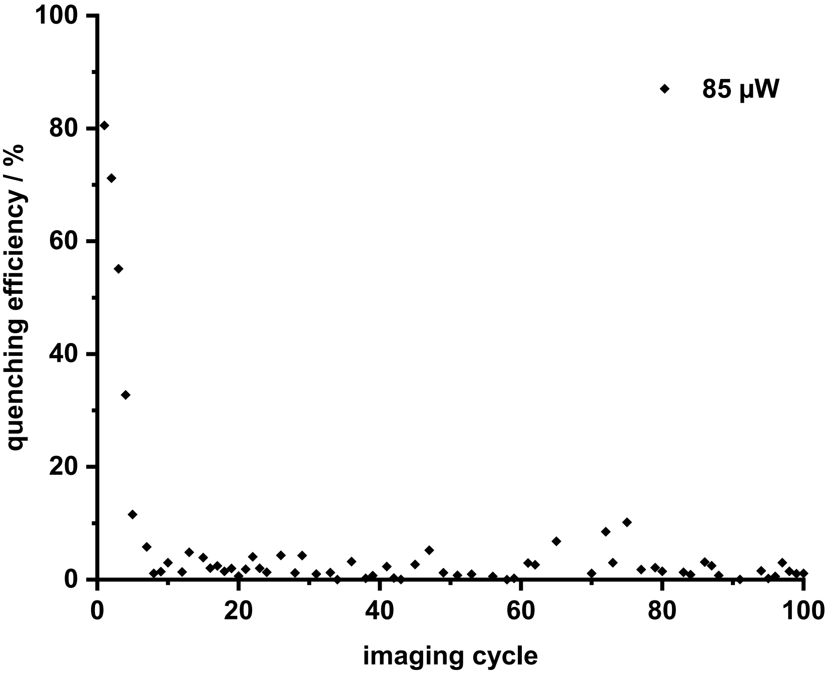

Figure S41 Fatigue of a mixture dyad **3** and 'dummy' dyad **6** in a 1:1 ratio over 100 imaging cycles in GUVs at 85 µW 405 nm laser power (λ_{ex} 594 nm, 0.66 µW, pixel dwell time 1.58 µs, pixel size 80 nm).

Another possibility is that diffusion of non-degraded dyad molecules from outside the imaging plane can replenish the partially-degraded population in the imaging plane between the imaging cycles. For this to be true, the rate of diffusion would need to be slower than the time required to acquire a single image (otherwise no dark image would be observed) but fast enough to replenish the dyad over the course of the imaging cycles. This would result in an initial period where fresh dyad replaces degraded dyad in the imaging plane, meaning minimal loss in quenching efficiency. After a period of time, the supply of fresh dyad is exhausted, and the quenching efficiency falls. We tested this hypothesis using supported lipid bilayers (SLBs; see ESI Section 6 for preparation), in which all the dyad molecules in the membrane were irradiated in each cycle, leaving no 'spare' pool out of the imaging plane from which to replenish between imaging cycles. There is still the possibility for diffusion between pixels during the raster scanning, as with imaging GUVs. However, diffusion of molecules that have already been irradiated will lead to poorer observed quenching efficiency. We observed that the fatigue curves in SLBs were still sigmoidal, but that the initial period before the decay began appeared shorter for SLBs than for GUVs (Figure S42–S43). These results confirm that replenishment by diffusion is occurring but that it is not the only process which contributes to the sigmoidal shape.

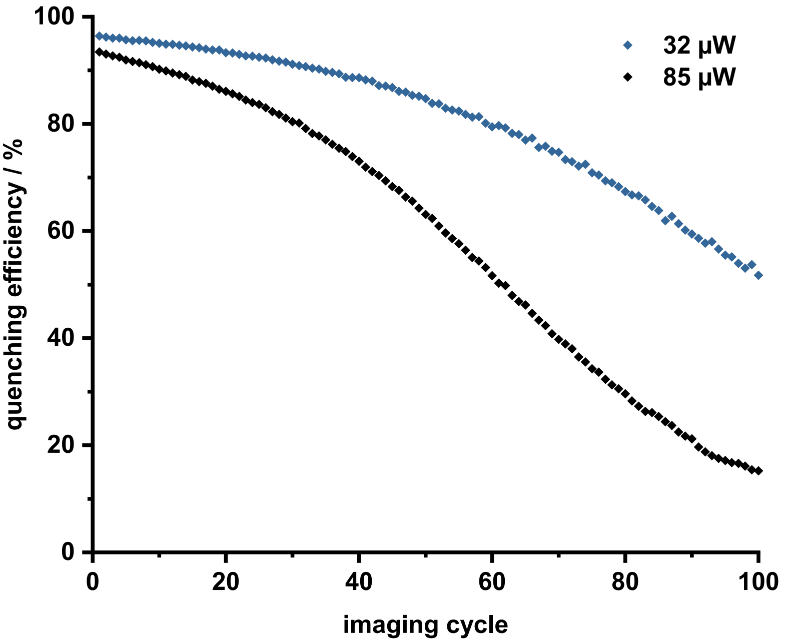

Figure S42 Fatigue of dyad **1** over 100 imaging cycles in supported lipid bilayers at various 405 nm laser powers (λ_{ex} 594 nm, 0.66 µW power, pixel dwell time 1.58 µs, pixel size 80 nm).

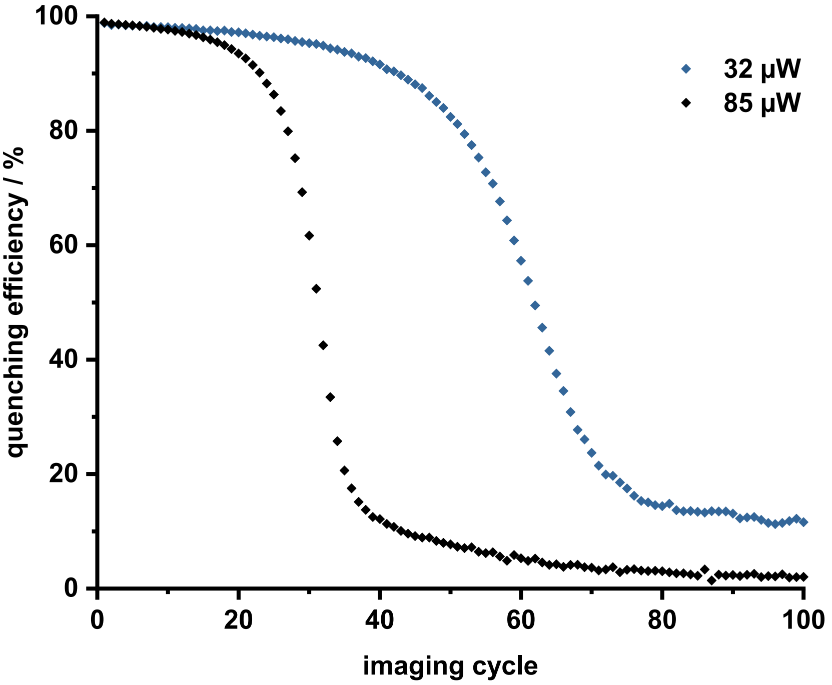

Figure S43 Fatigue of dyad **3** over 100 imaging cycles in supported lipid bilayers at various 405 nm laser powers (λ_{ex} 594 nm, 0.66 µW power, pixel dwell time 1.58 µs, pixel size 80 nm).

8. Modeling Sigmoidal Fatigue

We can use a simple kinetic model to describe the sigmoidal appearance of the quenching efficiency fatigue. In the following explanation, we assume that the rate of switch fatigue is constant.

In the simplest example, we can consider that molecules behave independently of each other. We start with a state (A) in which the switch is capable of quenching the dye so there is no emission. Our concentration of quenched species is 1. Our switches are degraded with rate constant *k* to a state (B) where the switch cannot quench the dye, so the dye becomes emissive. The concentration of quenching species follows 1st order kinetics, giving an exponential decay.

If our molecules interact in pairs, in which one switch can quench both the dyes in the pair, we have 3 possible states (Scheme S7): State A, where both switches are active so the system is fully quenched; State B, where one switch has been degraded but the other can still quench both dyes so the system is still dark; State C where both switches are degraded so the system becomes emissive.

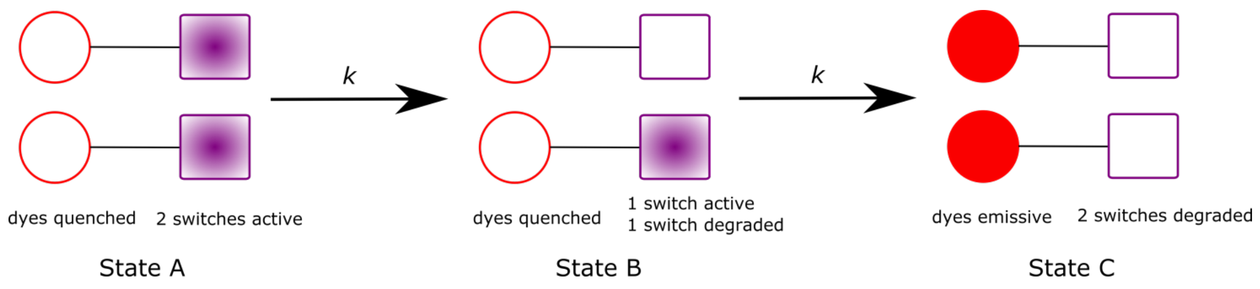

Scheme S7 Illustrative representation of kinetic model used to model the shape of quenching efficiency fatigue curves for an aggregate of two dyad molecules.

Assuming that the total concentration is given as [A] + [B] + [C] = 1, our concentration of quenched species is now [A] + [B] = 1 – [C]. This produces a set of differential equations which can be solved graphically (using Berkeley Madonna v. 9.1.14 software):

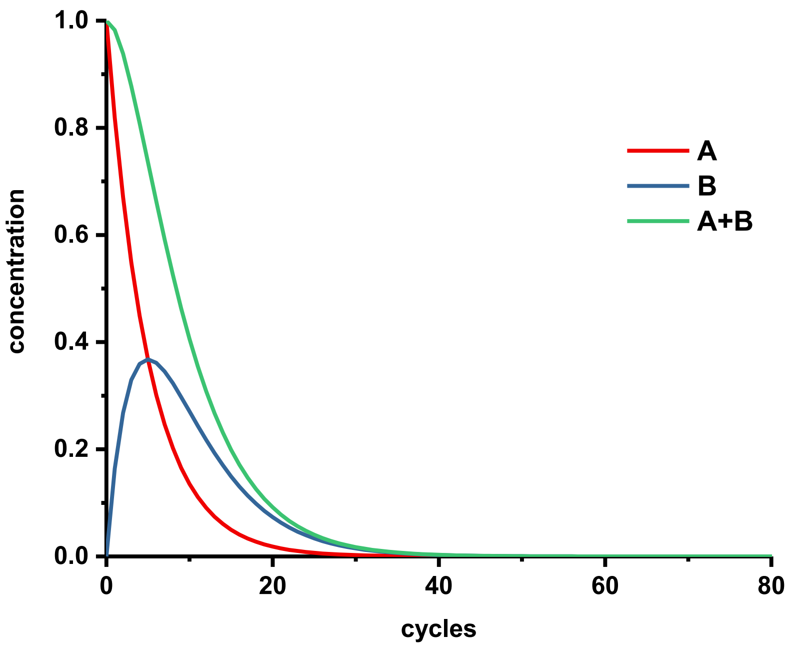

Figure S43 Calculated concentrations of the States A and B (see Scheme S7).

We can extend this principle to any number of interacting molecules, *n*. As you increase the number of interacting molecules, the initial period of high quenching efficiency gets longer (Figure S45).

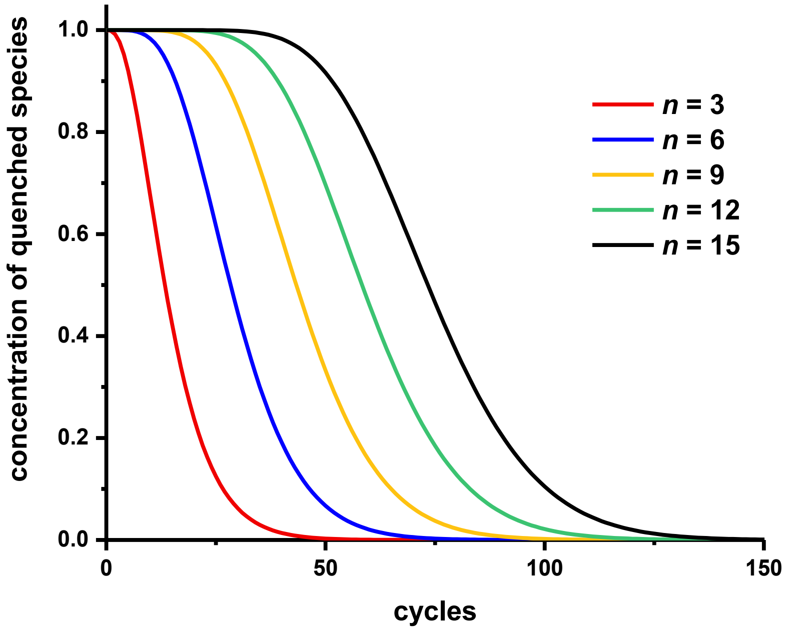

Figure S45 Extension of the modeling for different numbers of interacting molecules sizes, where one switch is capable of quenching n dyes.

Unfortunately, because of the competing diffusion replenishment processes, it is impossible to fit our experimental data using these simple models, but the models do demonstrate that cooperative quenching between molecules leads to sigmoidal fatigue behavior.

9. Fluorescence Correlation Spectroscopy

For details of sample preparation for FCS measurements see Supporting Information Section 6. FCS measurements were carried out on a Zeiss LSM880 inverted laser scanning confocal microscope equipped with a 561 nm (HeNe) excitation laser. Data were acquired using an LD C-Apochromat 40×/1.1 NA water immersion objective. The microscope is operated via a PC running Zen software. FCS curves were fitted using FoCuS-point to extract transit times.[14] For each dyad, measurements were recorded on 10 different vesicles, with 5 repeats on each vesicle. In all cases FCS was measured at the top membrane of the vesicle.

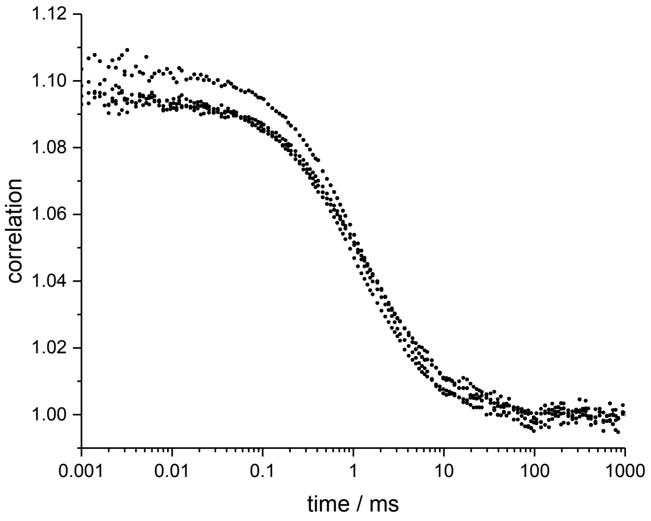

Figure S46 FCS autocorrelation curve for GUVs stained with **Atto565-DPPE**.

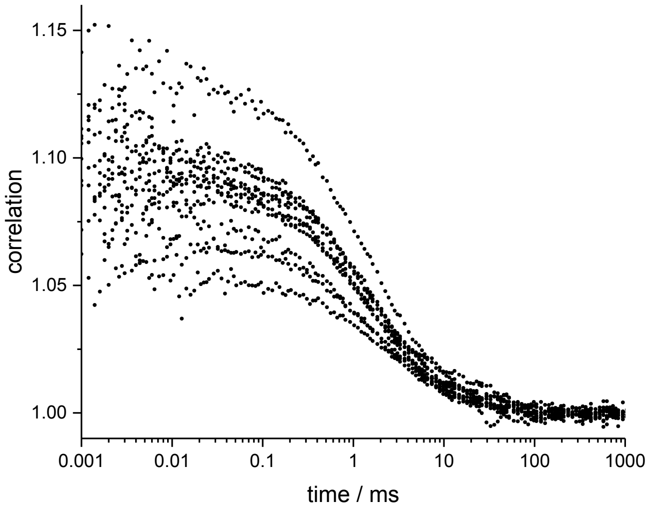

Figure S47 FCS autocorrelation curve for GUVs stained with dyad **1**.

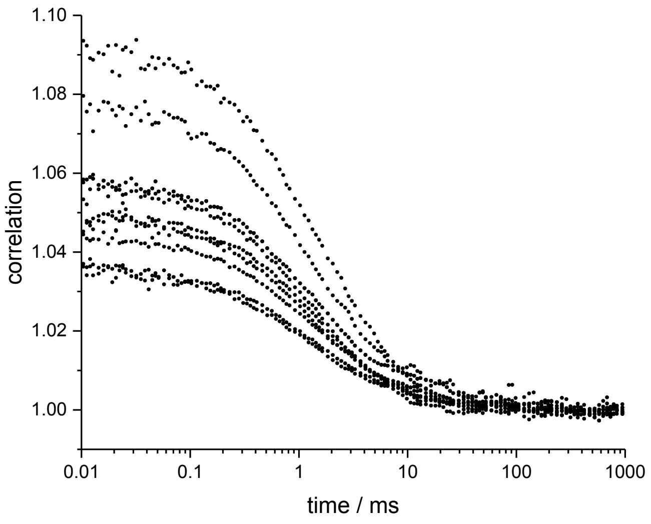

Figure S48 FCS autocorrelation curve for GUVs stained with dyad **2**.

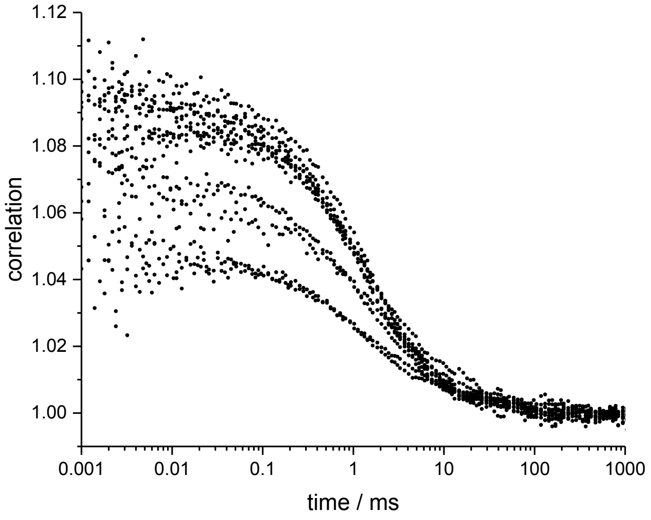

Figure S49 FCS autocorrelation curve for GUVs stained with dyad **3**.

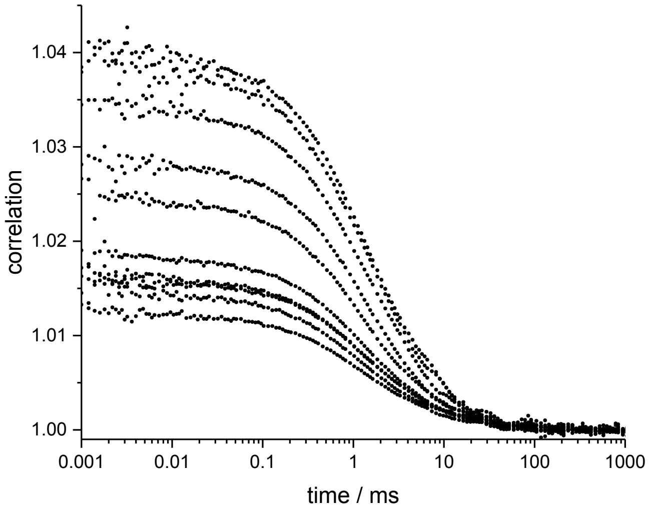

Figure S50 FCS autocorrelation curve for GUVs stained with dyad **4**.

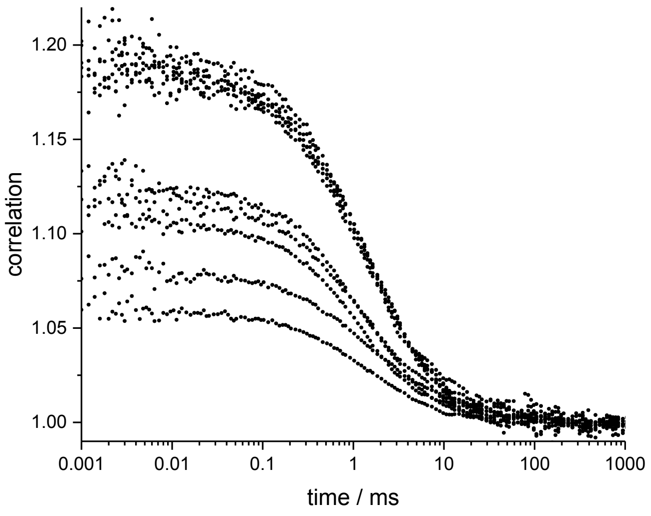

Figure S51 FCS autocorrelation curve for GUVs stained with dyad **5**.

Transit times determined from FCS measurements were used to calculate diffusion coefficients. Firstly, the size of the observation spot was first calibrated according to previously reported procedures.[15] A FWHM of 297 nm was measured for the observation spot, and diffusion coefficients, *D*, were calculated according to Equation S10, where τ_D is the transit time measured by FCS:

$$D = \frac{FWHM^2}{8\ln 2\tau_D} \quad \text{(Eq. S10)}$$

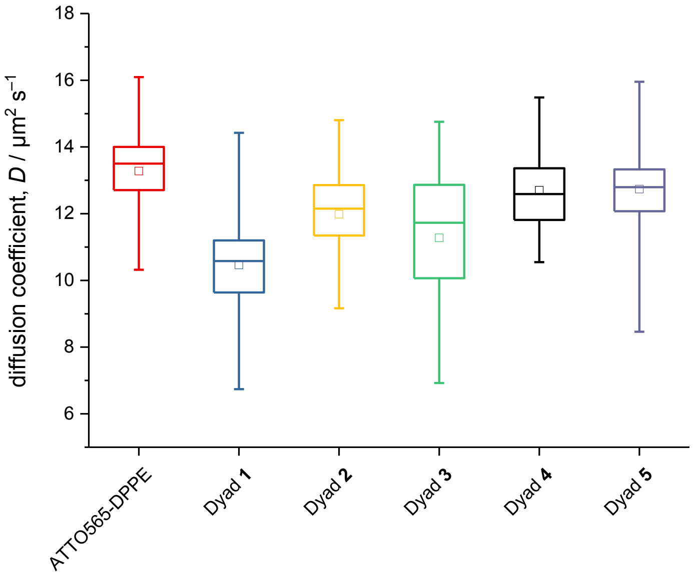

Figure S52 Diffusion coefficients of **ATTO-565-DPPE** and dyads **1–5** in GUVs. Data are shown as box-and-whisker plots showing median, first and third quartiles, and the mean (small squares). The whiskers represent the minimum and the maximum values.

10. HPLC Analysis

Reverse phase HPLC was performed at 298 K using an Agilent 1100 Series system comprising an autosampler (G1313A), a vacuum degassing unit (G1379A), a quaternary pump (G1311A), a column oven (G1316A), a diode array detector (G1315B), and a fraction collector (G1364C). The instrument was operated using ChemStation software. For analytical HPLC an Agilent Eclipse XDB-C18 column (4.6 × 150 mm, 5 µm particle size) was used with a flow rate of 1.0 mL/min. For semi-preparative HPLC an Agilent Eclipse XDB-C18 column (9.4 × 250 mm, 5 µm) was used with a flow rate of 4.18 mL/min.

Method A (analytical)

**Time / min**	**%H_2O**	**%CH_3OH**
0	95	5
20	0	100
35	0	100
37	95	5

Method B (semi-preparative)

**Time / min**	**%H_2O**	**%CH_3OH**
0	95	5
33	0	100
58	0	100
62	95	5

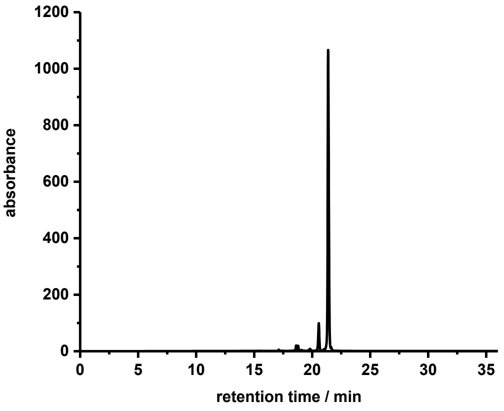

Figure S53 HPLC chromatogram of dyad **1** at 590 nm.

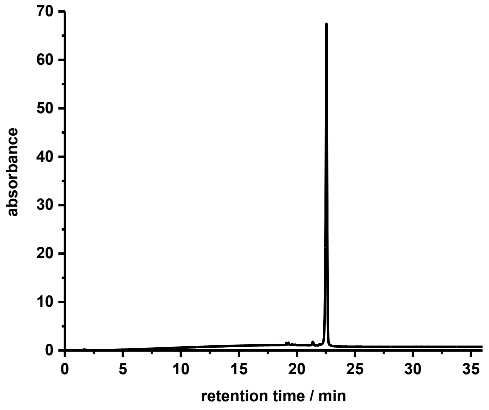

Figure S54 HPLC chromatogram of dyad **2** at 590 nm.

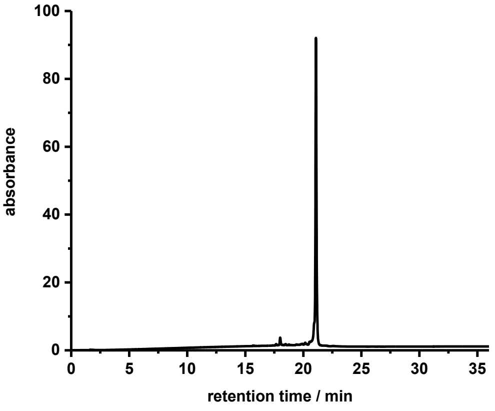

Figure S55 HPLC chromatogram of dyad **3** at 590 nm.

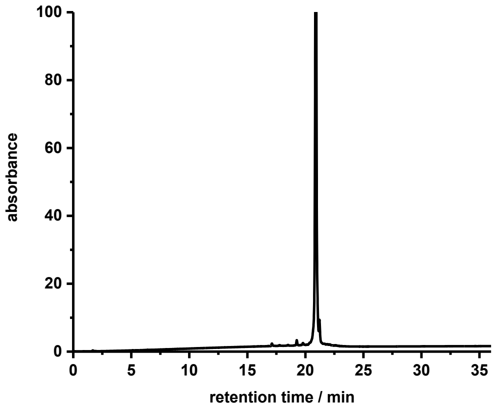

Figure S56 HPLC chromatogram of dyad **4** at 590 nm.

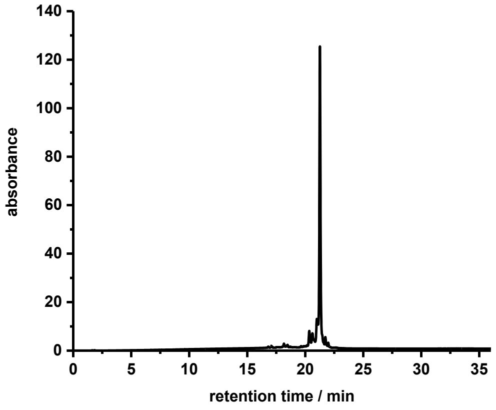

Figure S57 HPLC chromatogram of dyad **5** at 590 nm.

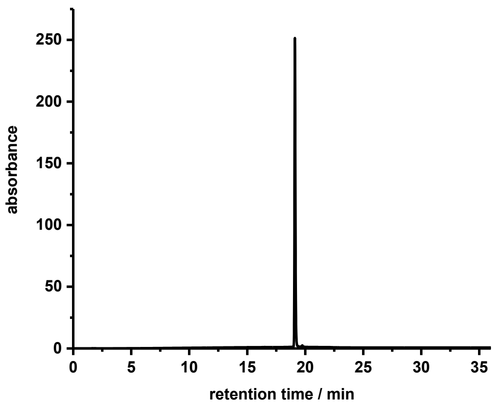

Figure S58 HPLC chromatogram of dye **1a** at 590 nm.

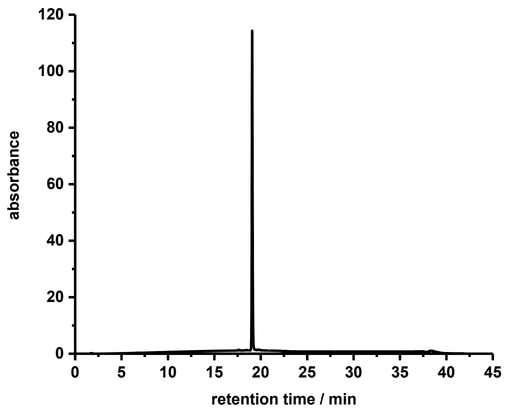

Figure S59 HPLC chromatogram of dye **2c** at 590 nm.

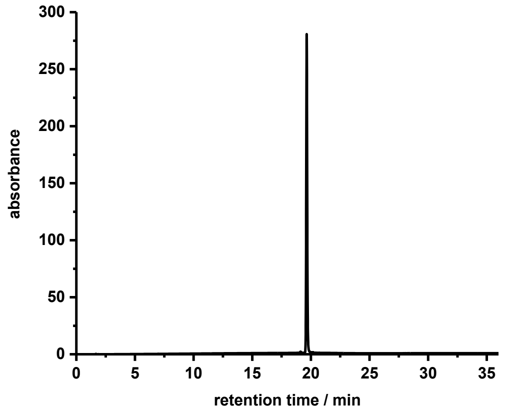

Figure S60 HPLC chromatogram of dye **2d** at 590 nm.

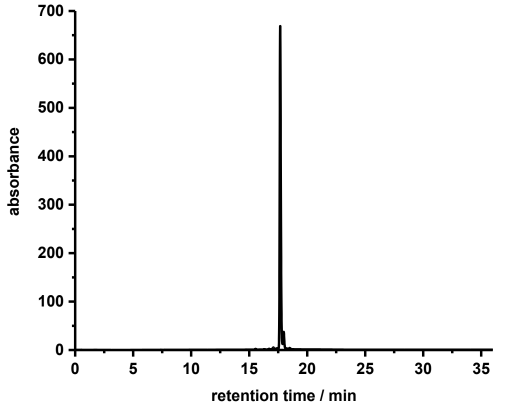

Figure S61 HPLC chromatogram of dye **3a** at 590 nm.

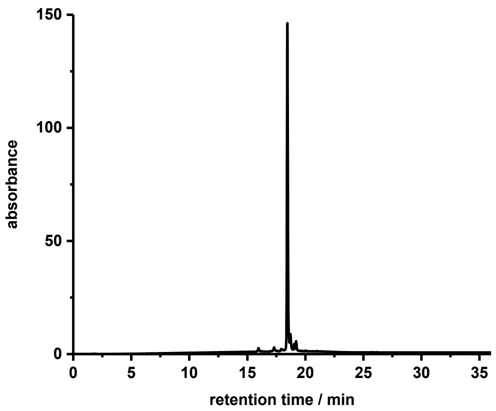

Figure S62 HPLC chromatogram of dye **4a** at 590 nm.

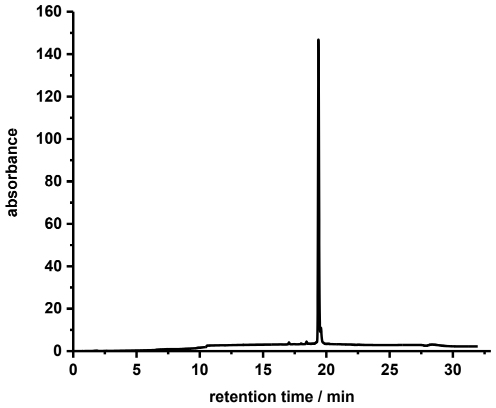

Figure S63 HPLC chromatogram of dye **5a** at 590 nm.

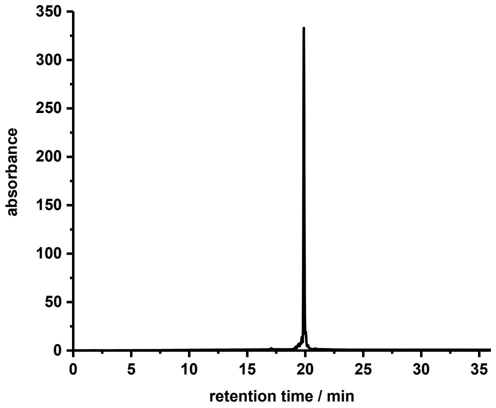

Figure S64 HPLC chromatogram of dye **10** at 590 nm.

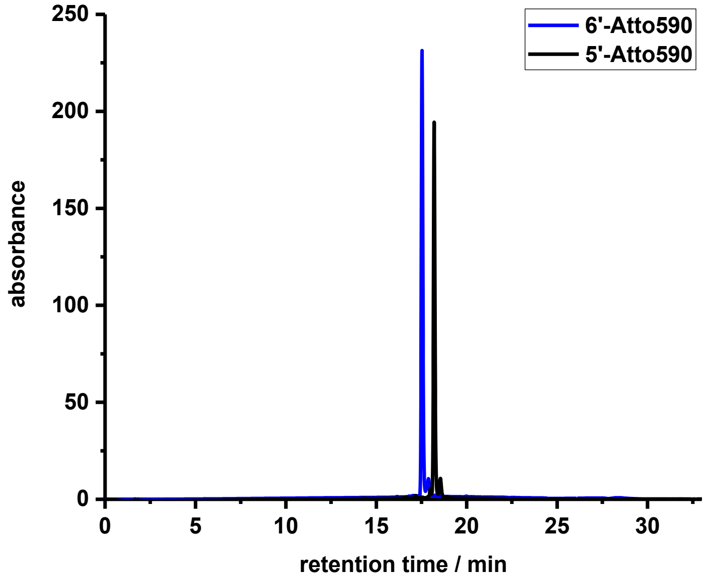

Figure S65 HPLC chromatogram of **5'-Atto590** (blue) and **6'-Atto590** (black) at 590 nm.

11. NMR Spectra

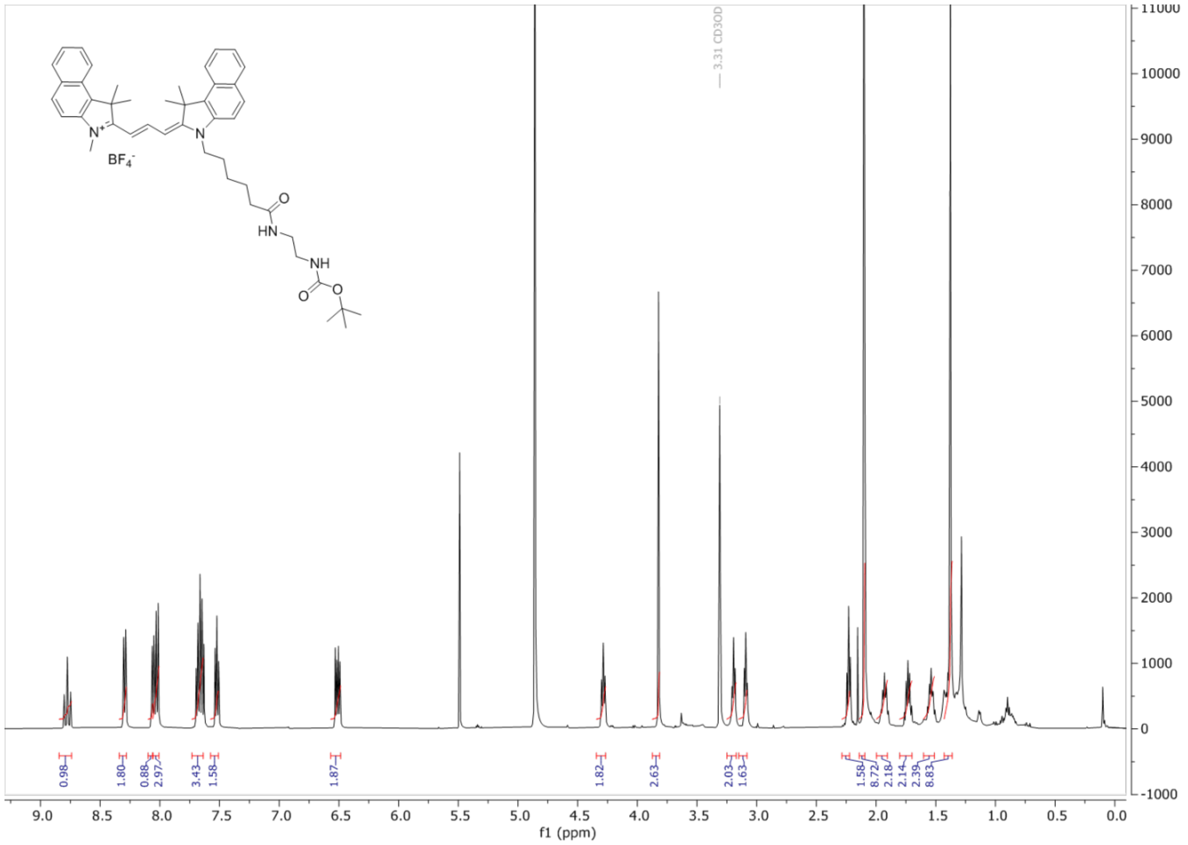

Figure S66 ^{1}H NMR spectrum of dye **1a** (CD_3OD, 500 MHz, 298 K).

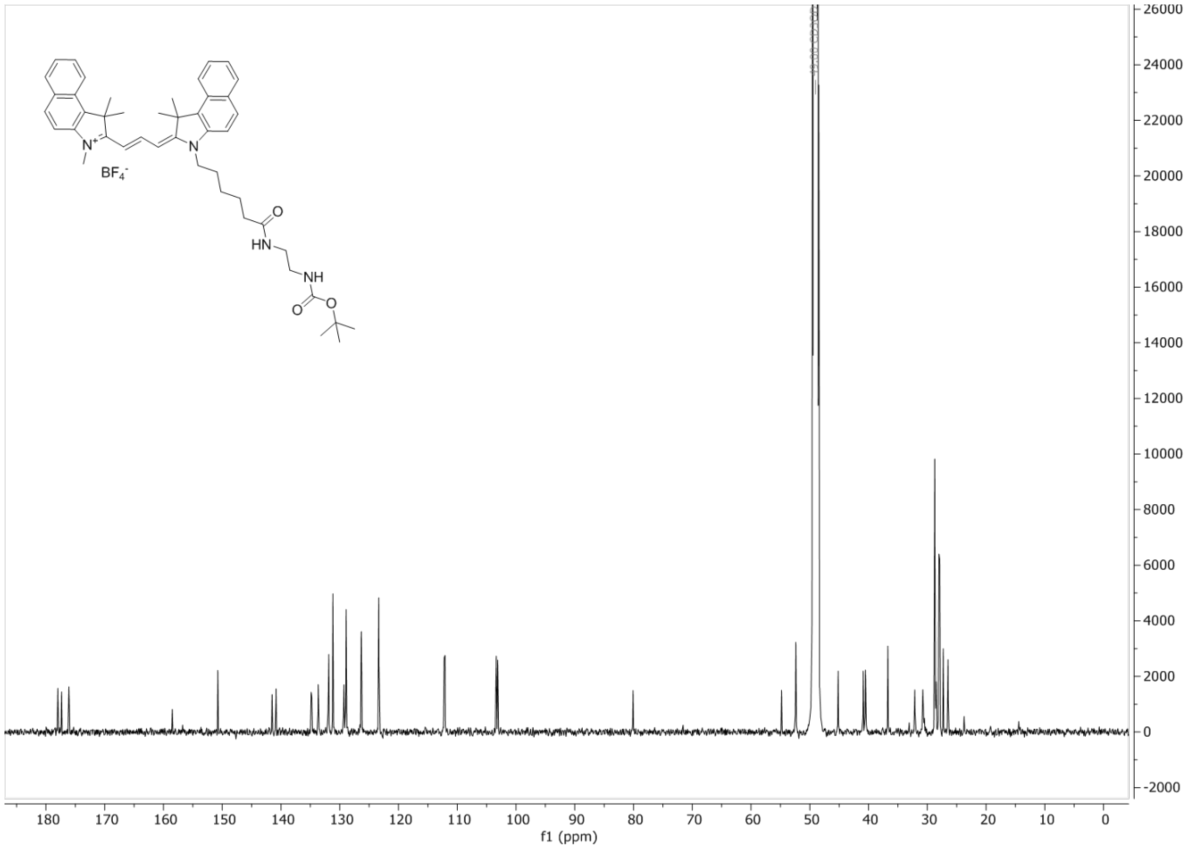

Figure S67 ^{13}C NMR spectrum of dye **1a** (CD_3OD, 126 MHz, 298 K).

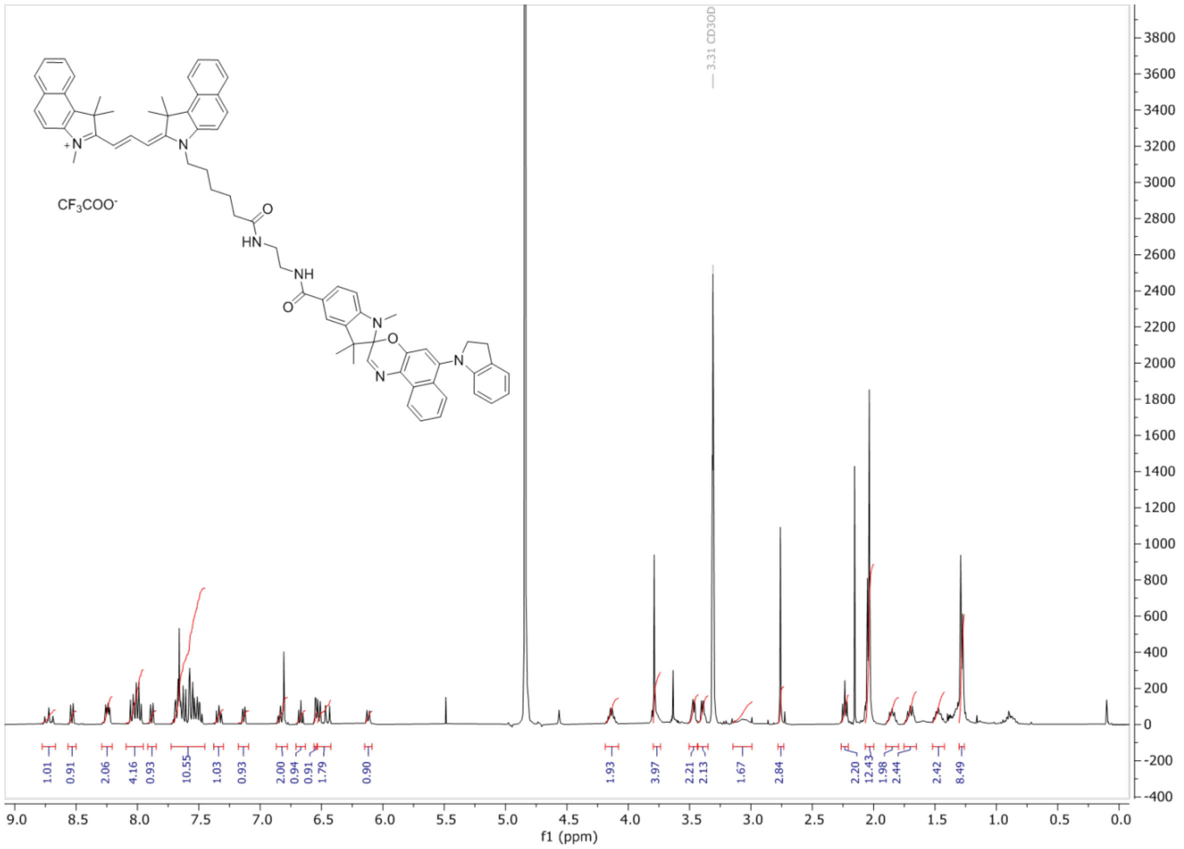

Figure S68 ^{1}H NMR spectrum of dyad **1** (CD_3OD, 500 MHz, 298 K).

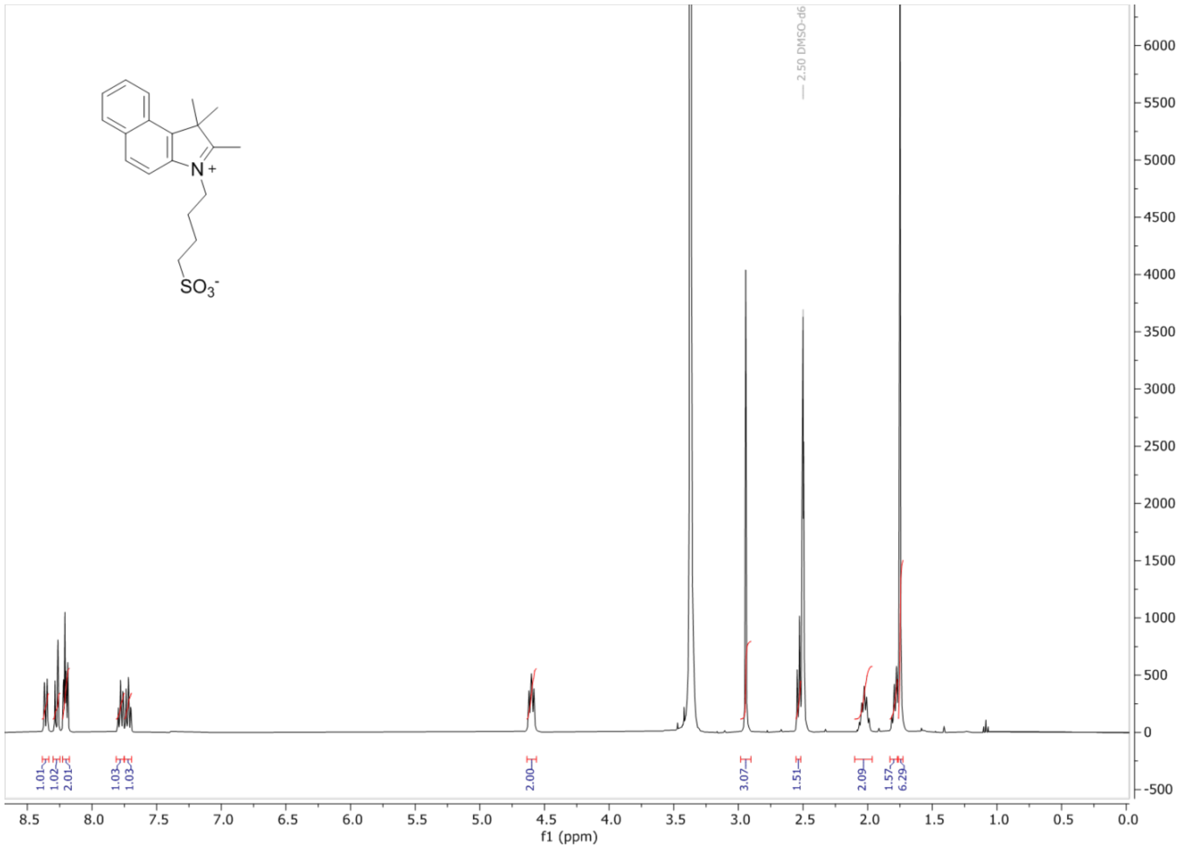

Figure S69 ^{1}H NMR spectrum of compound **2a** (DMSO-d_6, 400 MHz, 298 K).

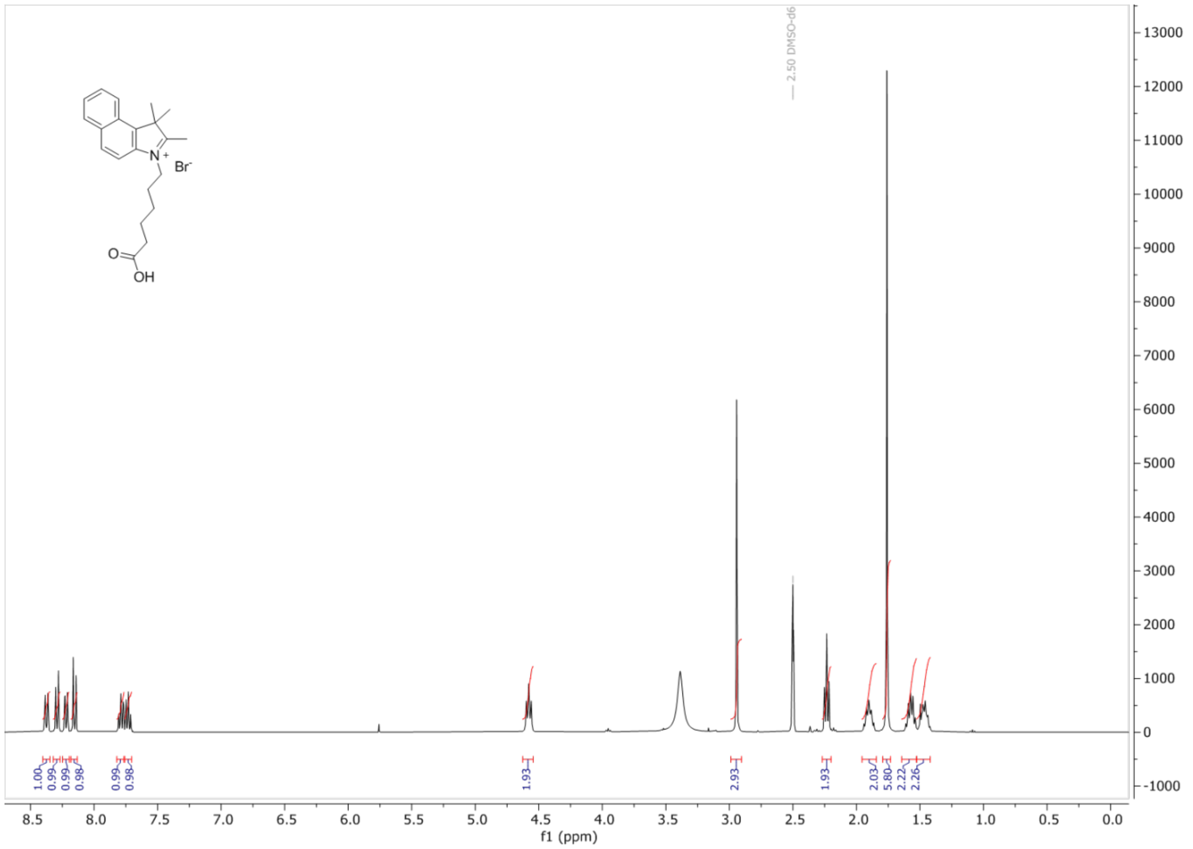

Figure S70 ^{1}H NMR spectrum of compound **2b** (DMSO-d_6, 400 MHz, 298 K).

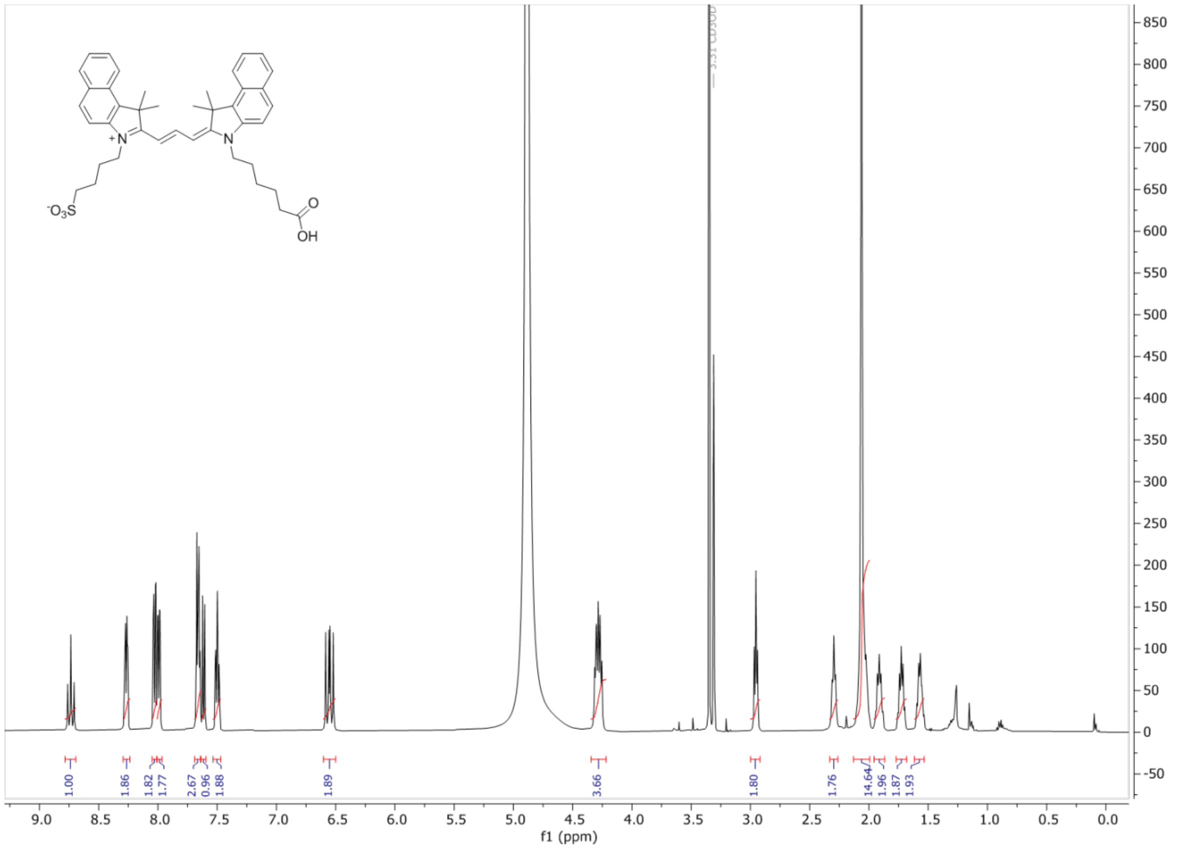

Figure S71 ^{1}H NMR spectrum of dye **2c** (CD_3OD, 500 MHz, 298 K).

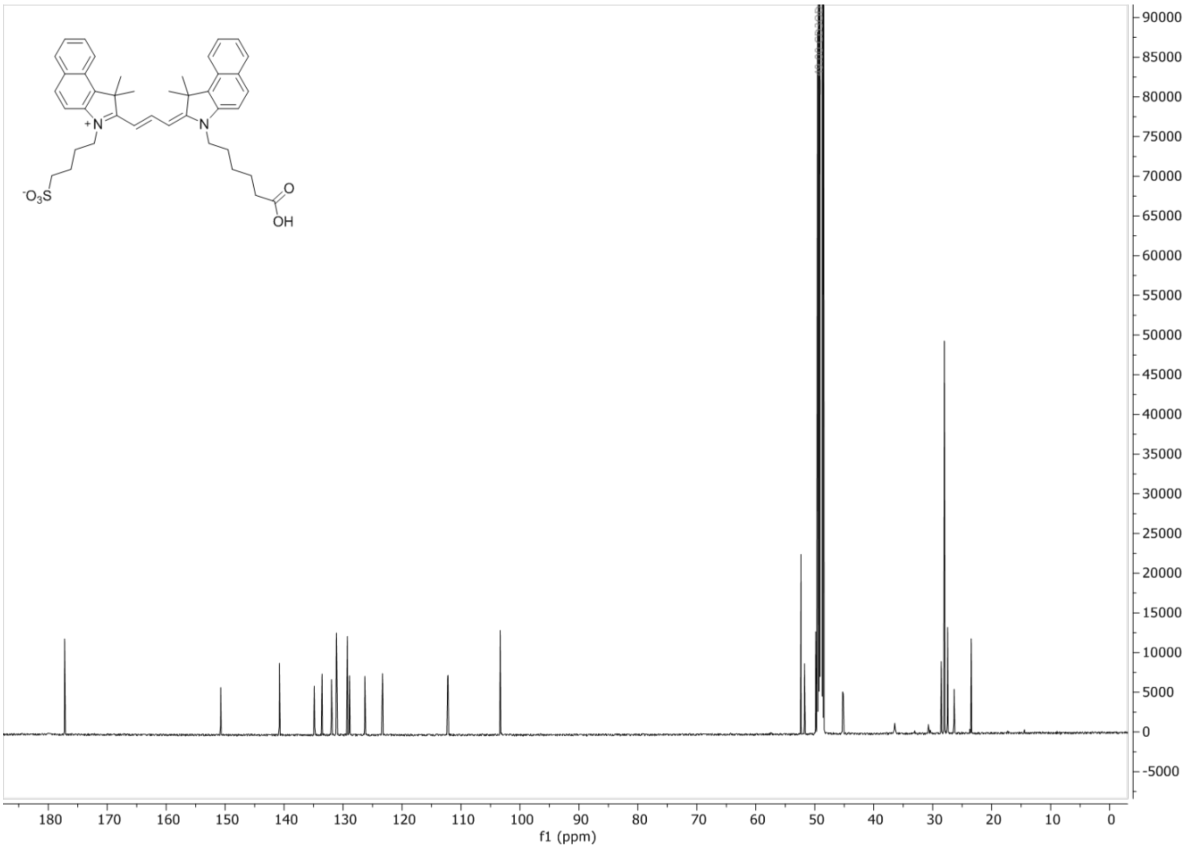

Figure S72 ^{13}C NMR spectrum of dye **2c** (CD_3OD, 126 MHz, 298 K).

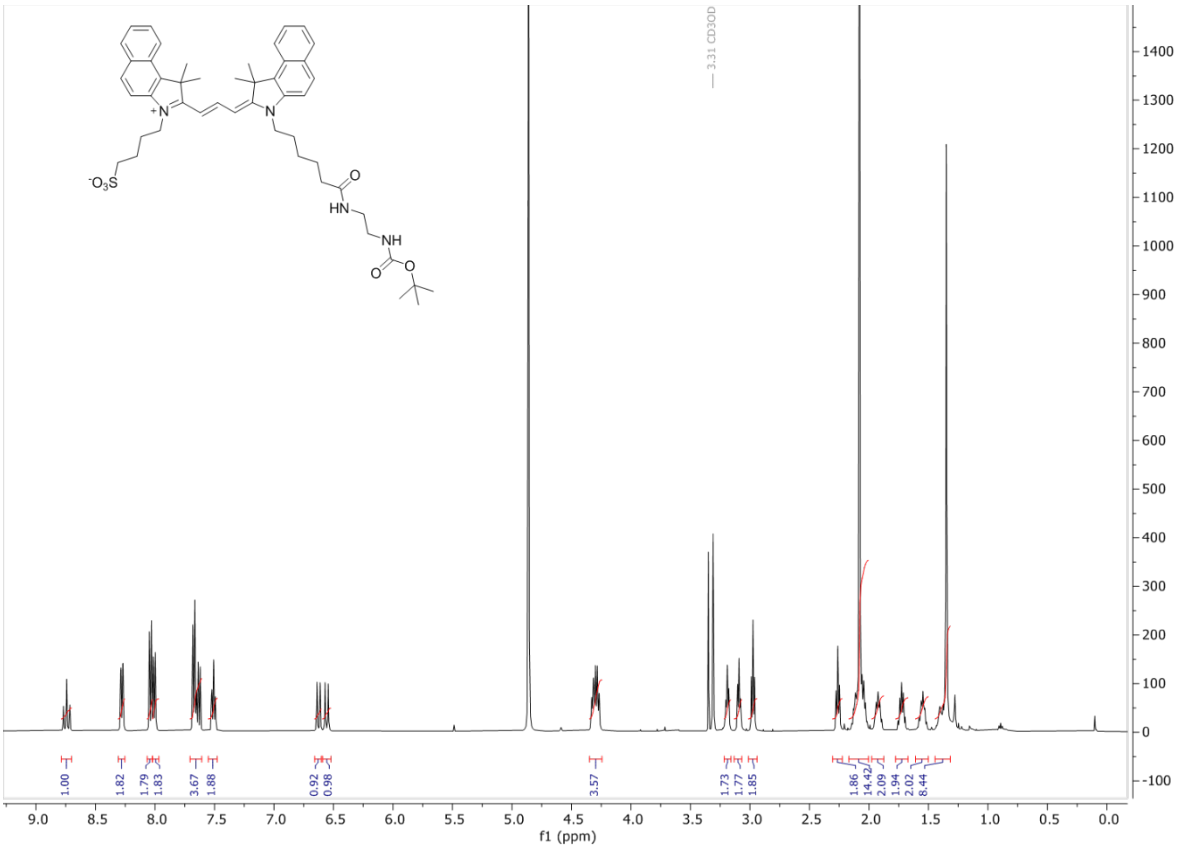

Figure S73 ^{1}H NMR spectrum of dye **2d** (CD_3OD, 500 MHz, 298 K).

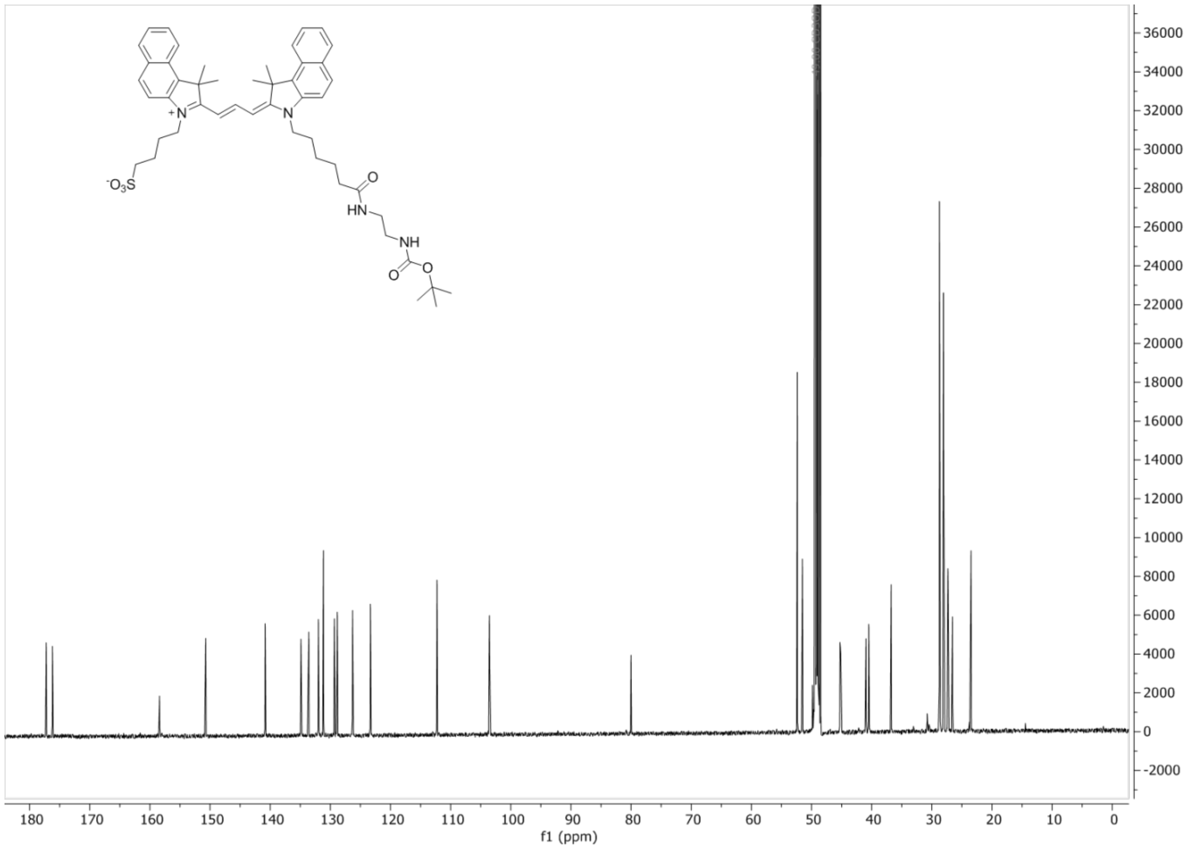

Figure S74 ^{13}C NMR spectrum of dye **2d** (CD_3OD, 126 MHz, 298 K).

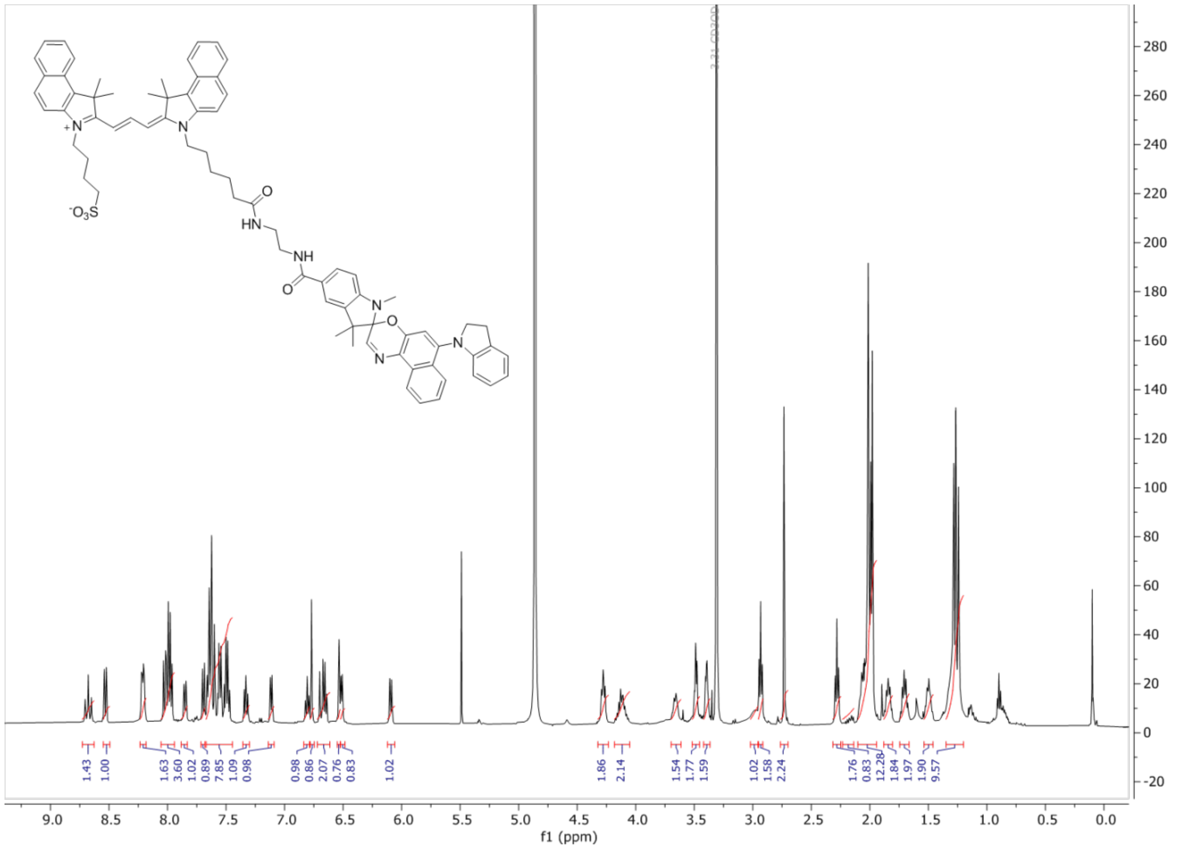

Figure S75 ^{1}H NMR spectrum of dyad **2** (CD_3OD, 400 MHz, 298 K).

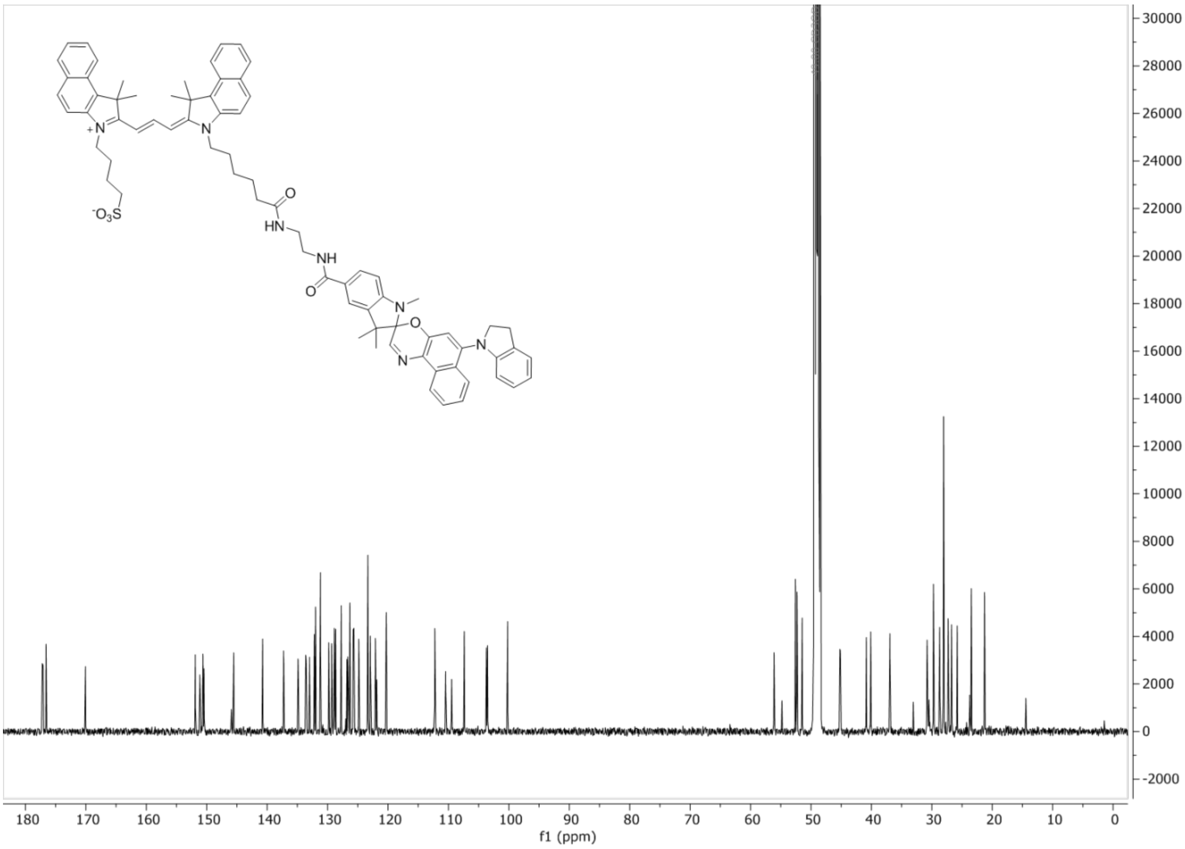

Figure S76 ^{13}C NMR spectrum of dyad **2** (CD_3OD, 126 MHz, 298 K).

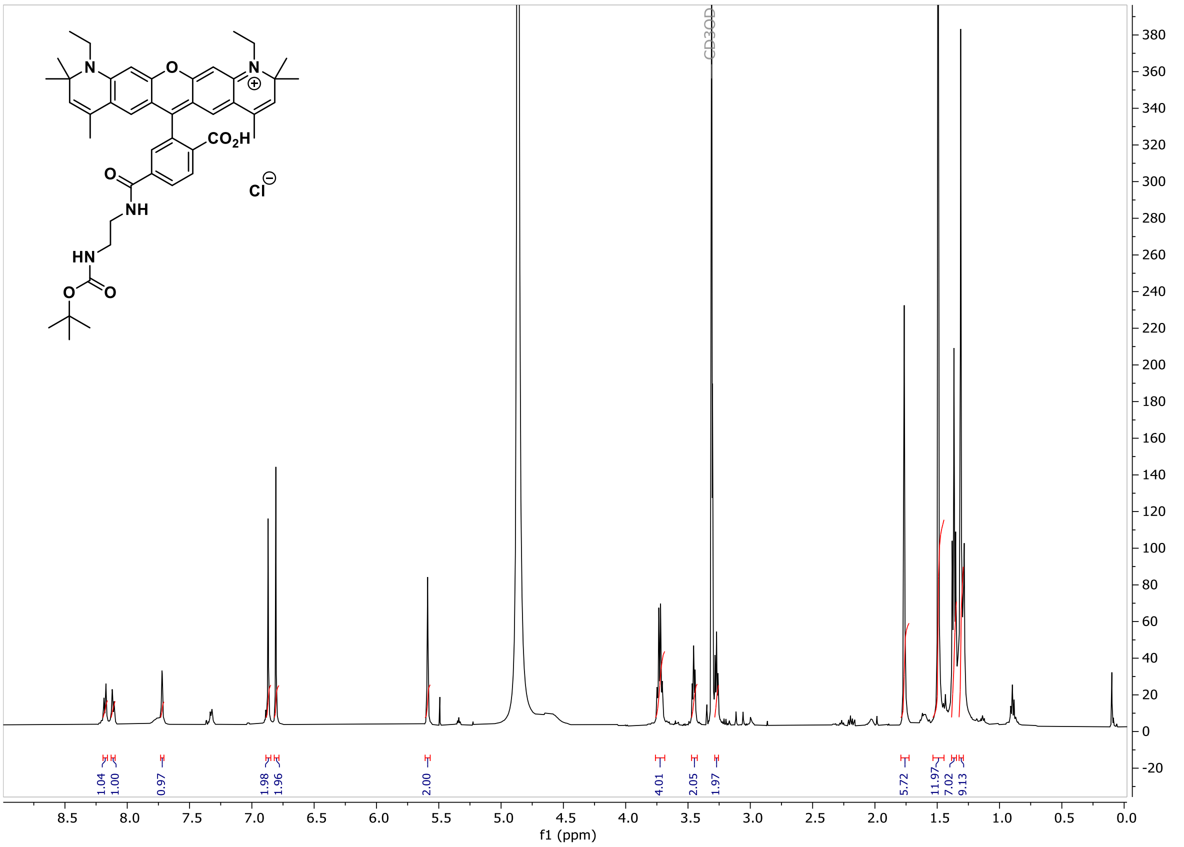

Figure S77 ^{1}H NMR spectrum of dye **3a** (CD_3OD, 500 MHz, 298 K).

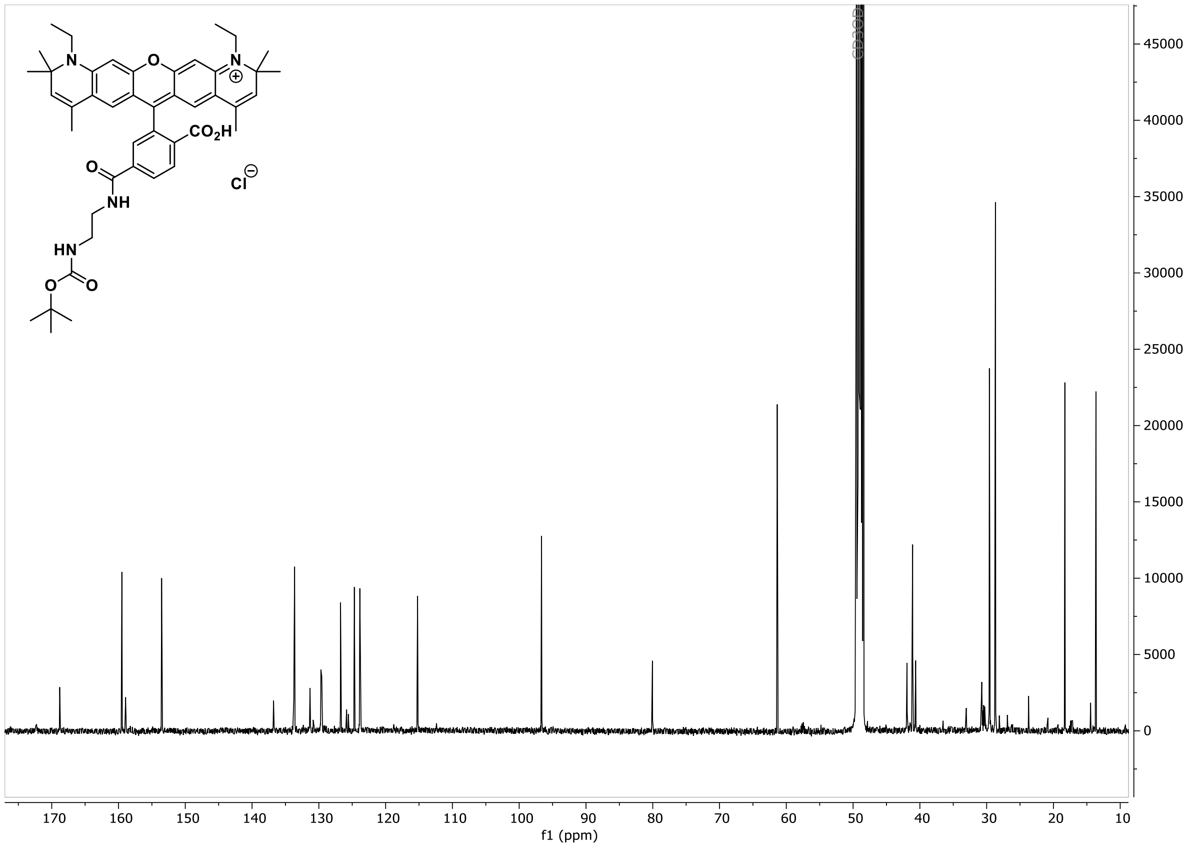

Figure S78 ^{13}C NMR spectrum of dye **3a** (CD_3OD, 126 MHz, 298 K).

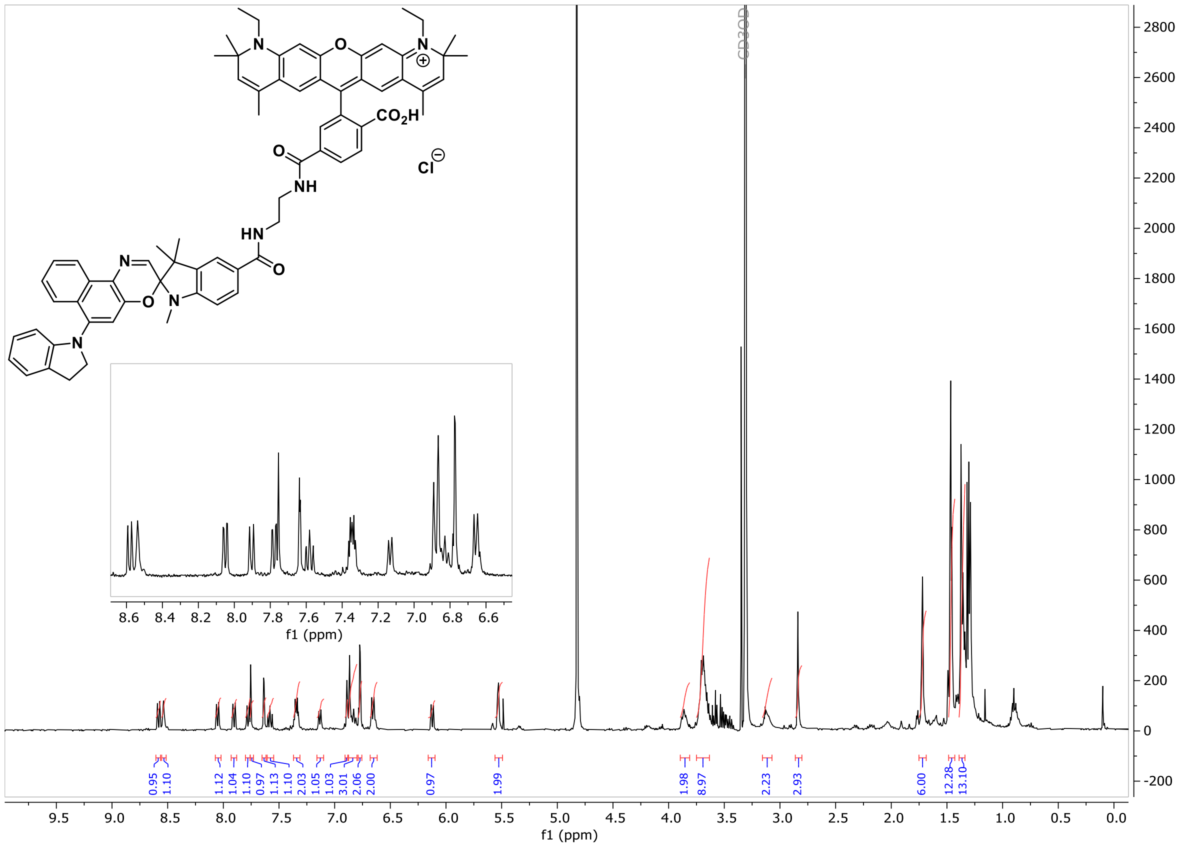

Figure S79 ^{1}H NMR spectrum of dyad **3** (CD_3OD, 400 MHz, 298 K).

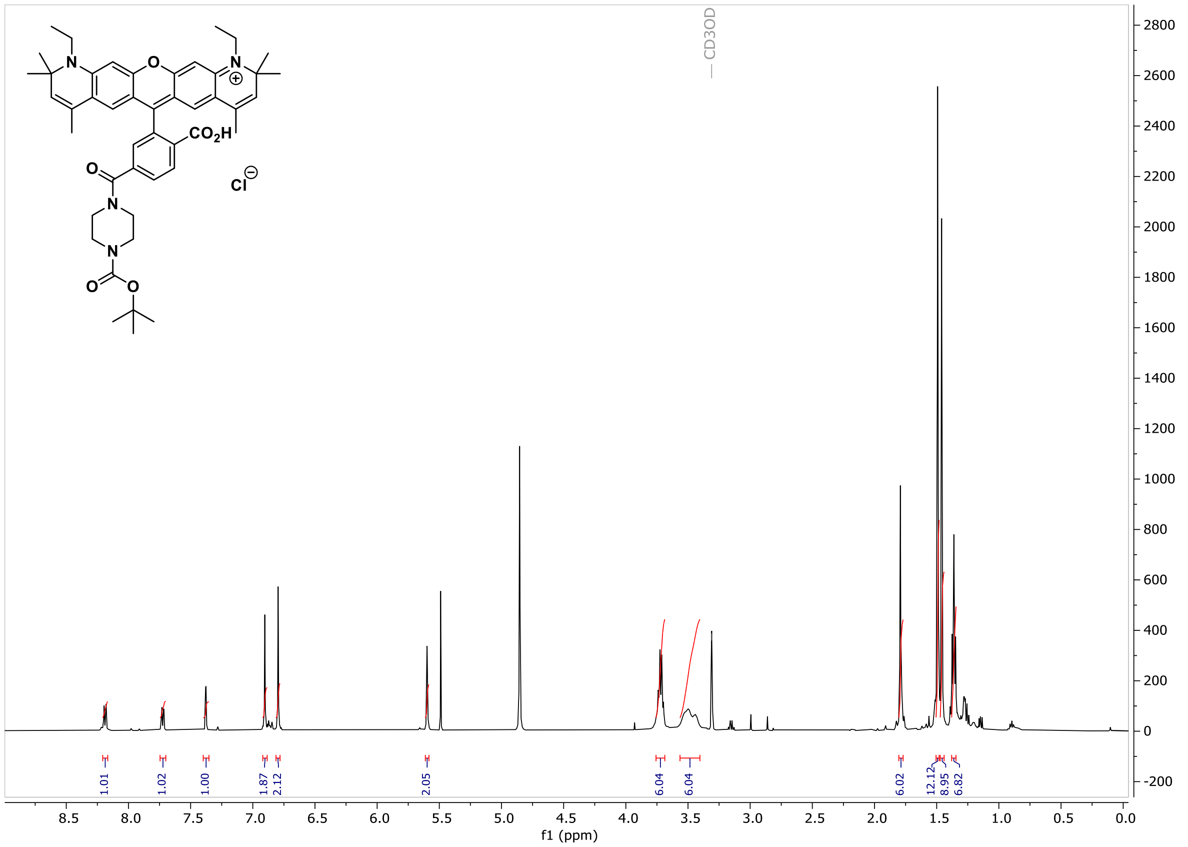

Figure S80 ^{1}H NMR spectrum of dye **4a** (CD_3OD, 500 MHz, 298 K).

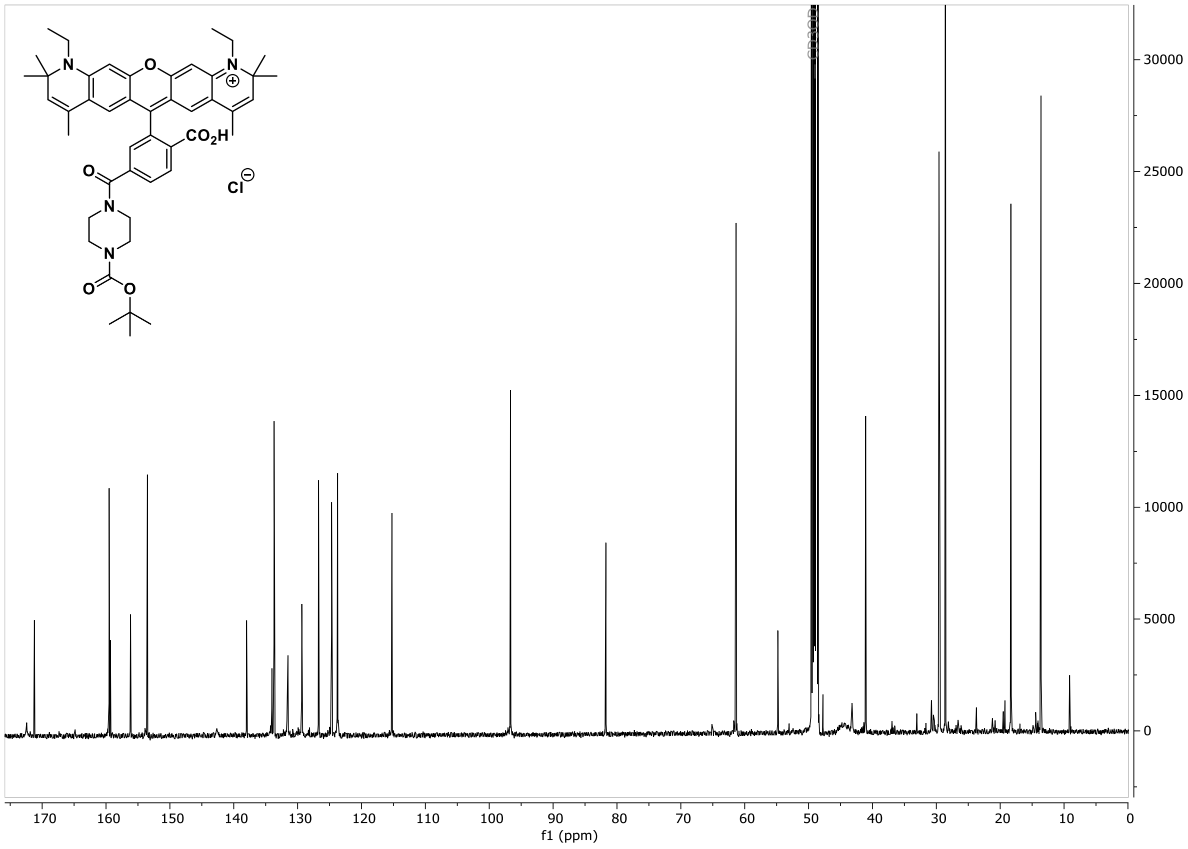

Figure S81 ^{13}C NMR spectrum of dye **4a** (CD_3OD, 126 MHz, 298 K).

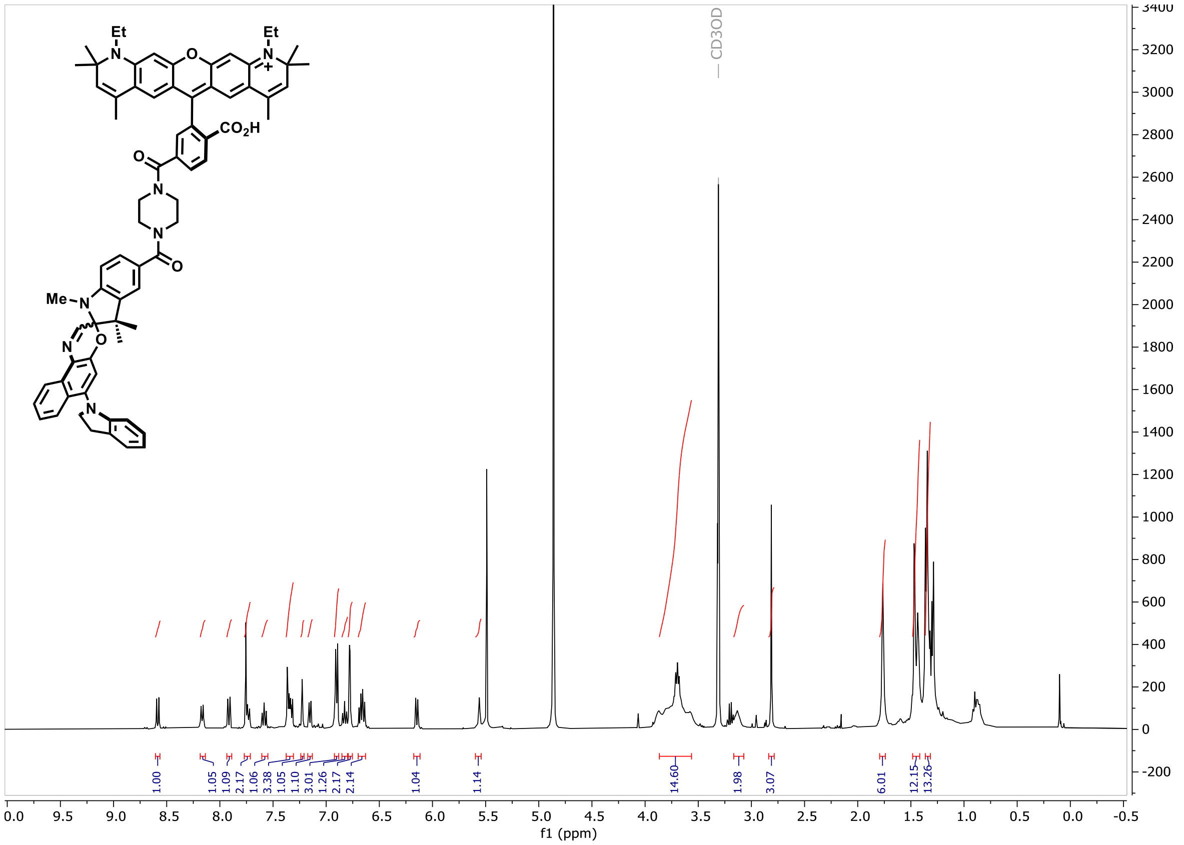

Figure S82 ^{1}H NMR spectrum of dyad **4** (CD_3OD, 500 MHz, 298 K).

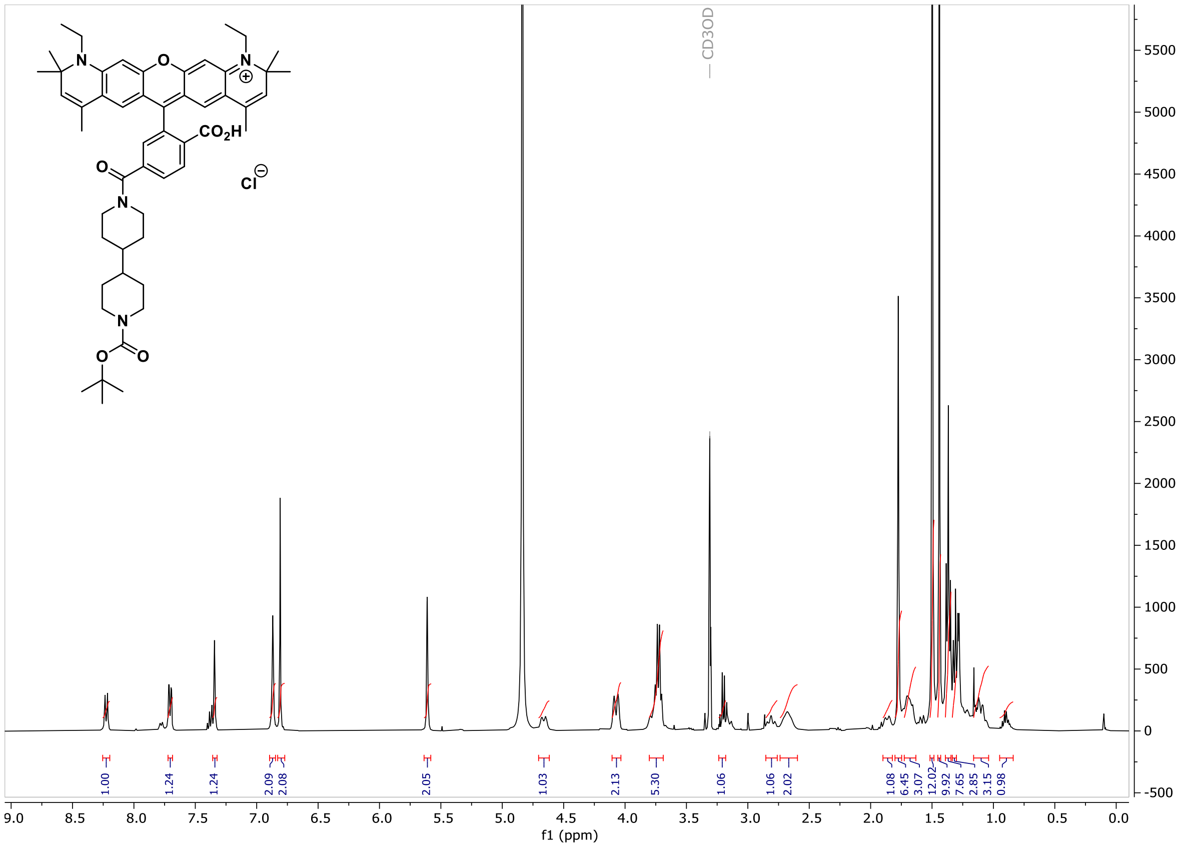

Figure S83 ^{1}H NMR spectrum of dye **5a** (CD_3OD, 500 MHz, 298 K).

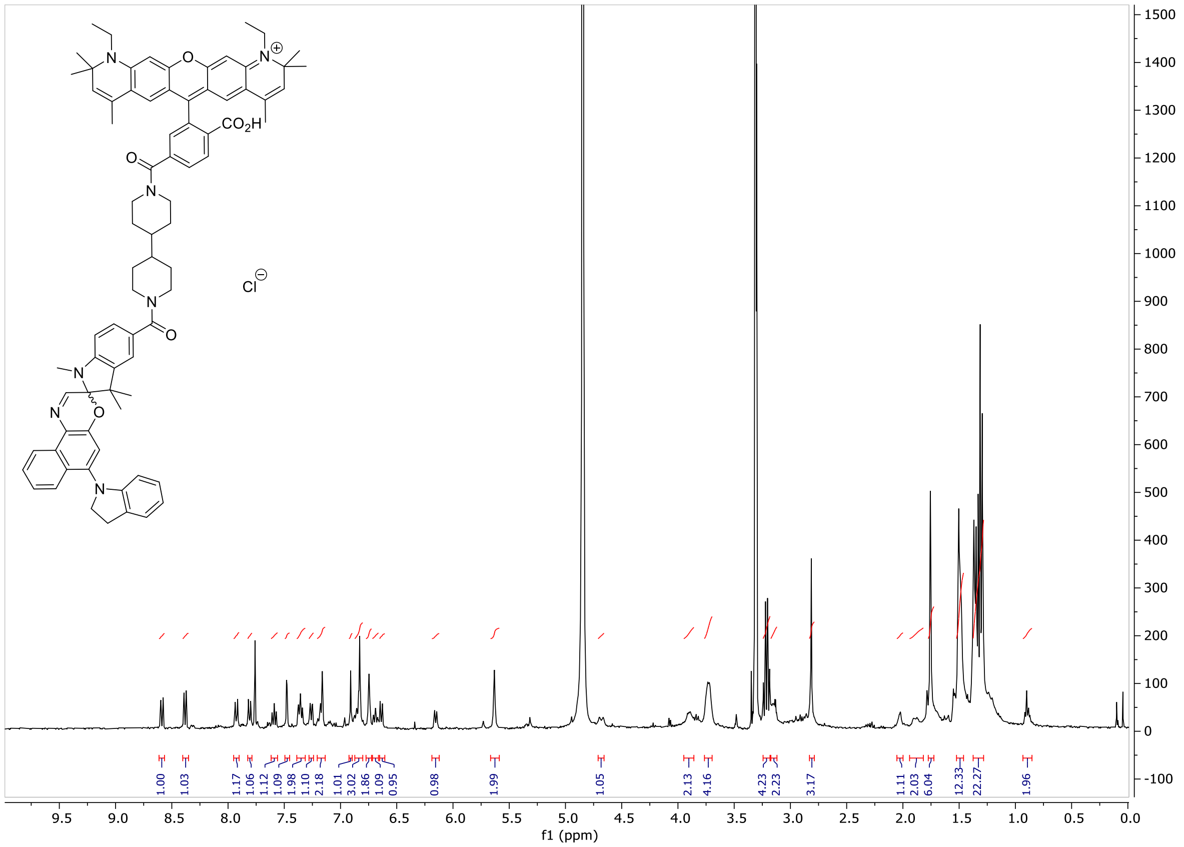

Figure S84 ^{1}H NMR spectrum of dyad **5** (CD_3OD, 500 MHz, 298 K).

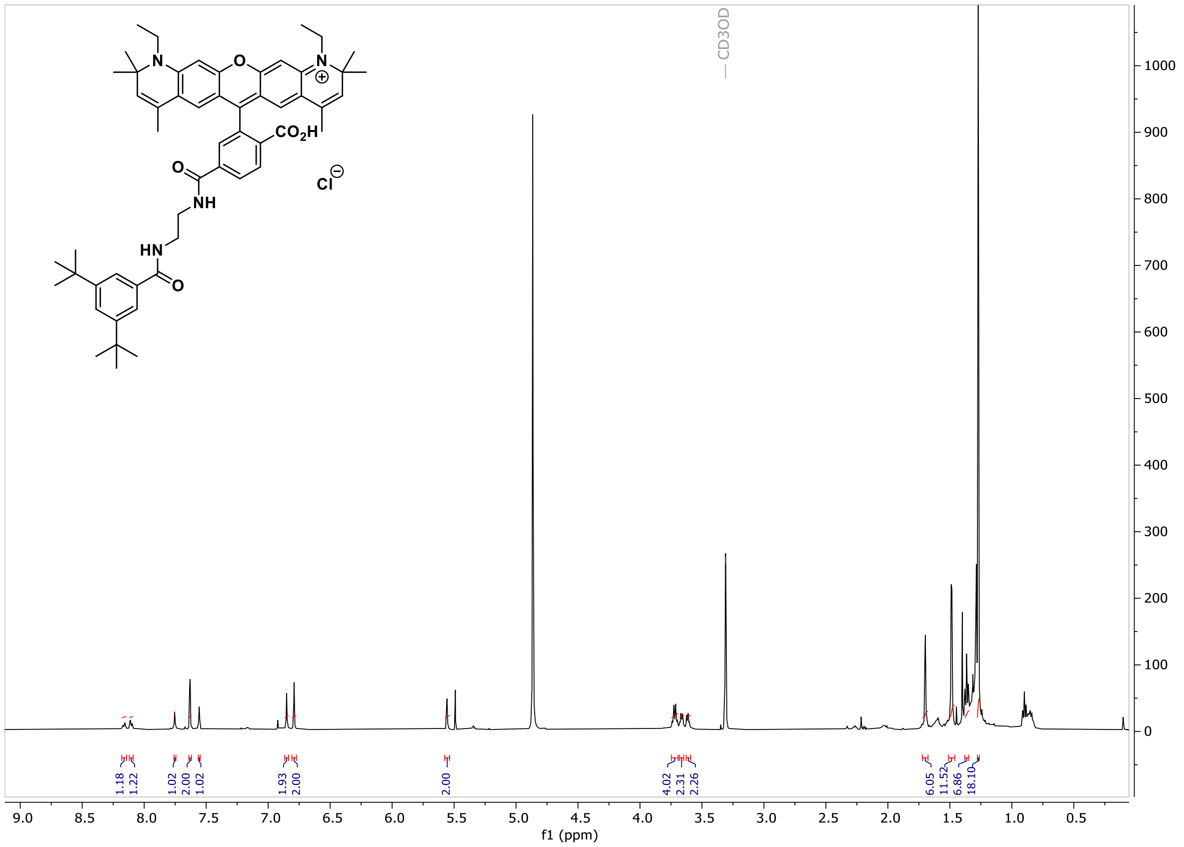

Figure S85 ^{1}H NMR spectrum of 'dummy dyad' **6** (CD_3OD, 500 MHz, 298 K).

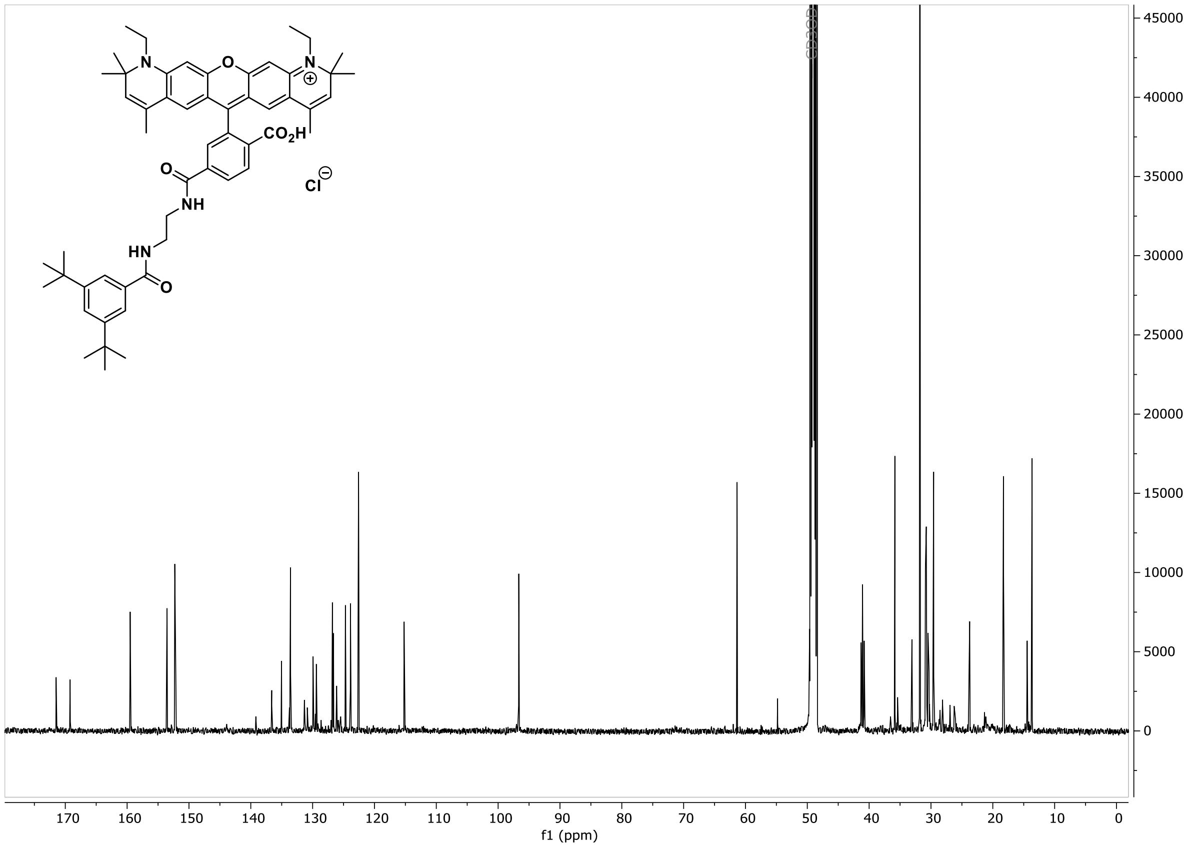

Figure S86 ^{13}C NMR spectrum of 'dummy dyad' **6** (CD_3OD, 126 MHz, 298 K).

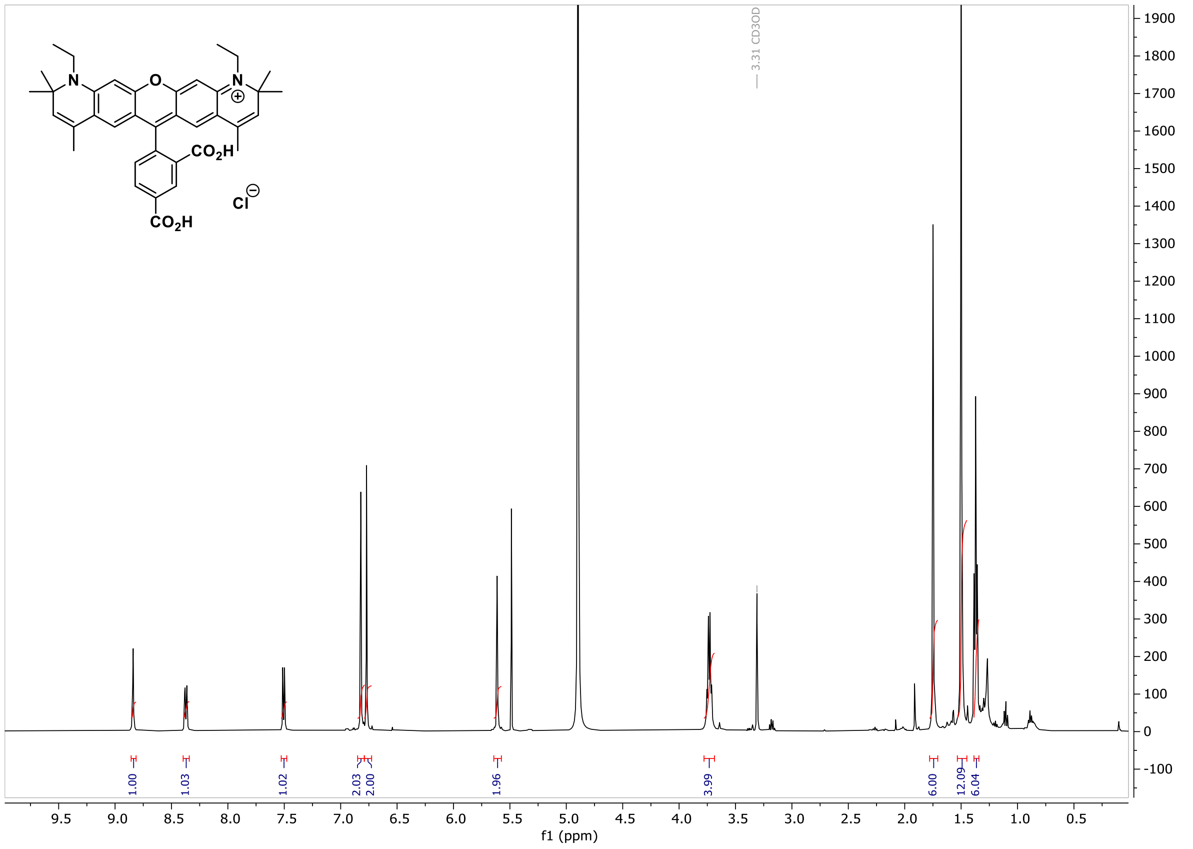

Figure S87 ^{1}H NMR spectrum of **5'-Atto590** (CD_3OD, 500 MHz, 298 K).

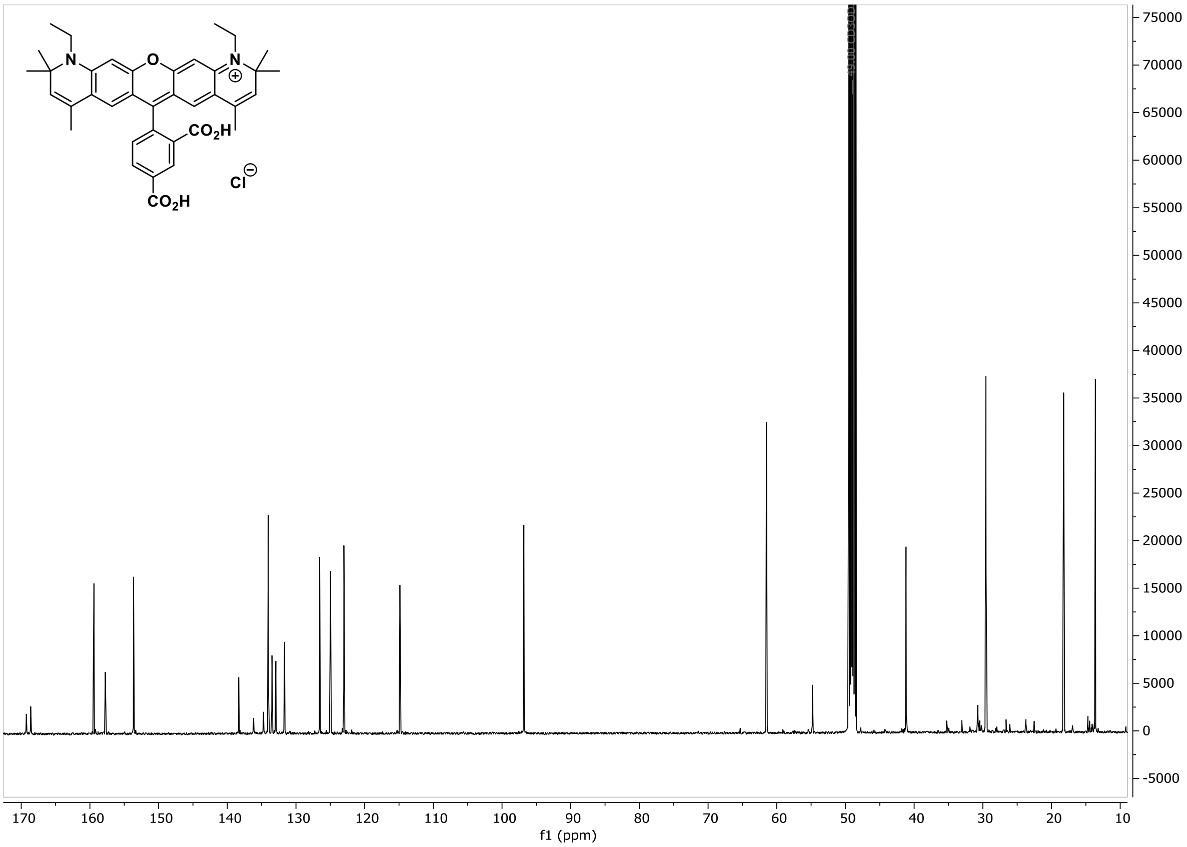

Figure S88 ^{13}C NMR spectrum of **5'-Atto590** (CD_3OD, 126 MHz, 298 K).

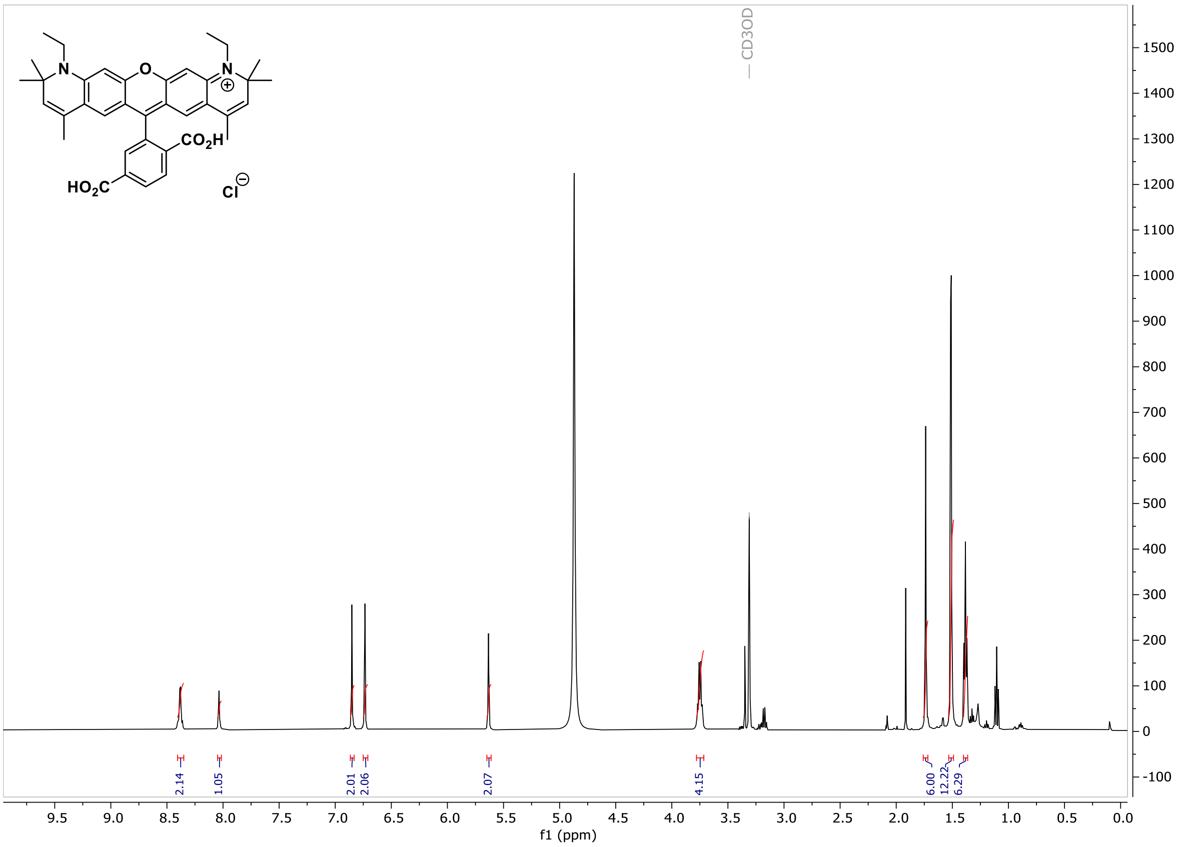

Figure S89 ^{1}H NMR spectrum of **6'-Atto590** (CD_3OD, 500 MHz, 298 K).

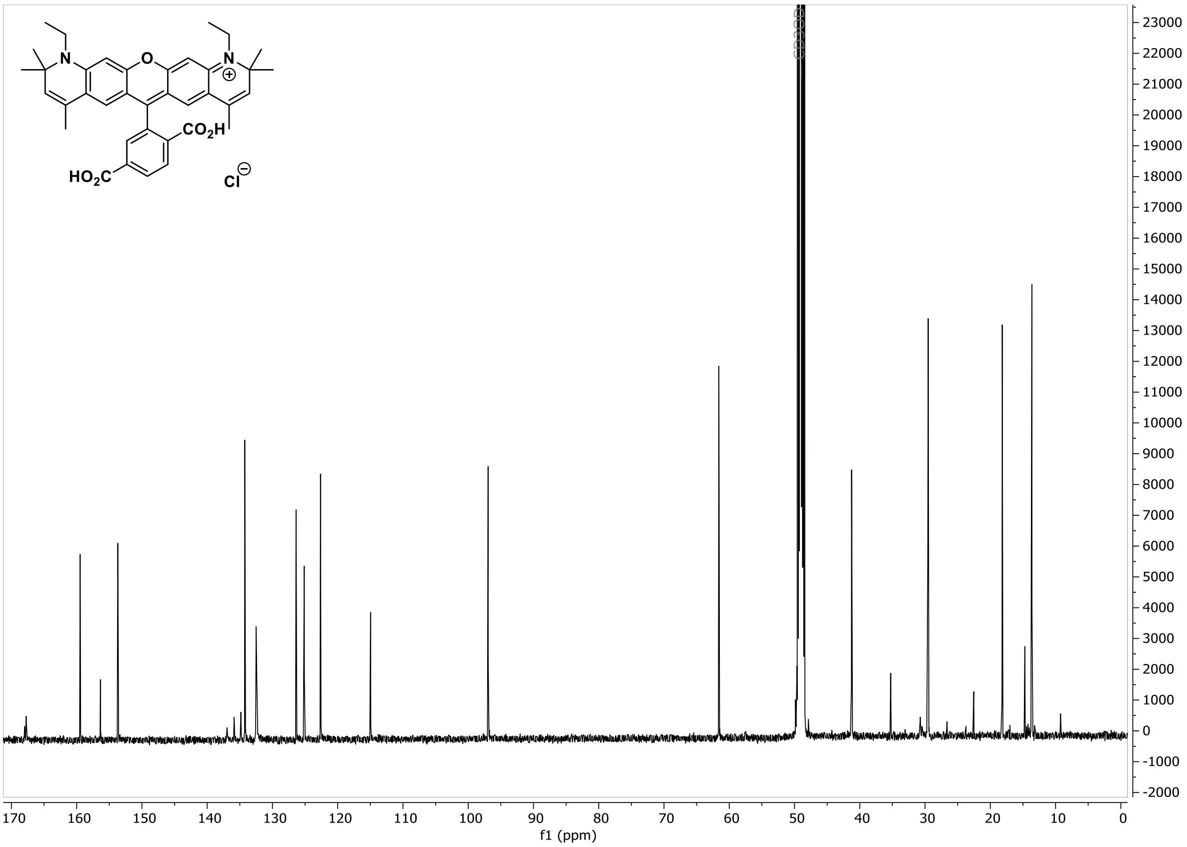

Figure S90 ^{13}C NMR spectrum of **6'-Atto590** (CD_3OD, 126 MHz, 298 K).

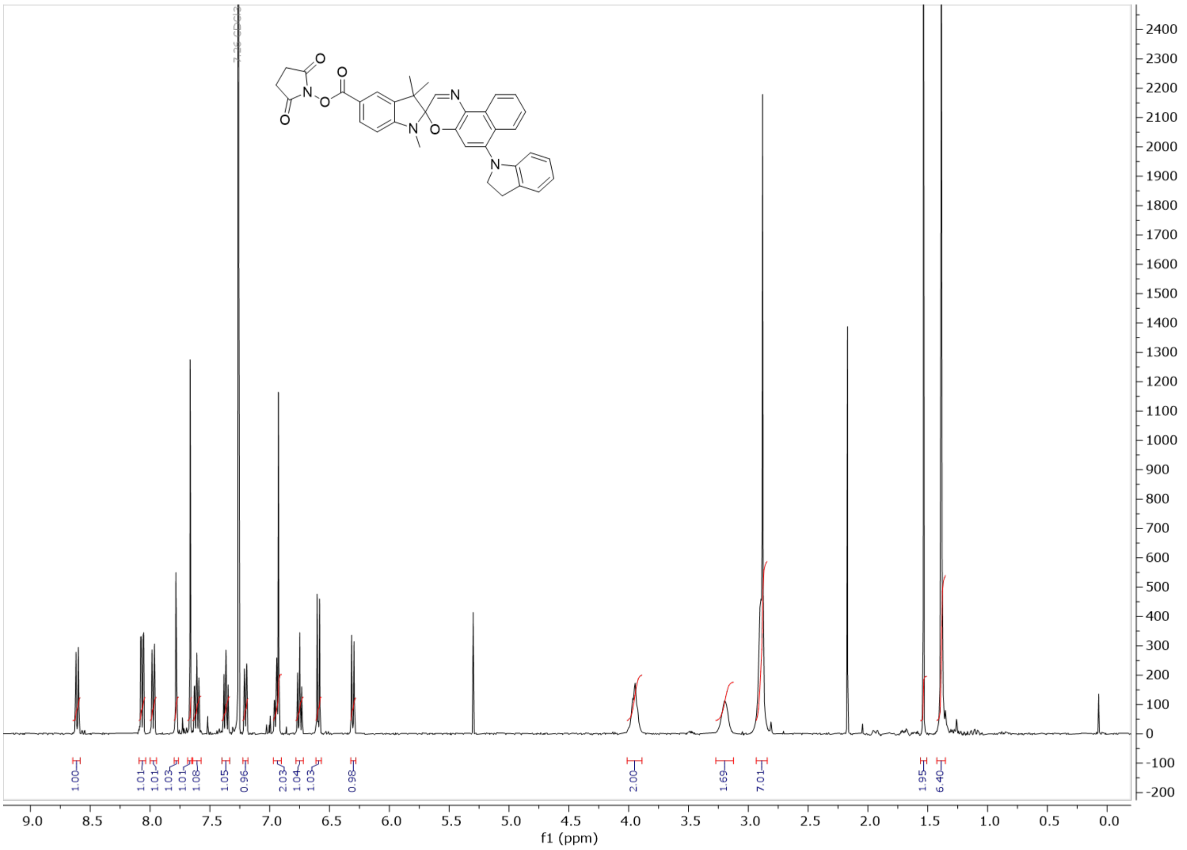

Figure S91 ^{1}H NMR spectrum of **SO-NHS** ($CDCl_3$, 400 MHz, 298 K).

12. References

1 D. M. Mizrahi, O. Ziv-Polat, B. Perlstein, E. Gluz and S. Margel, *Eur. J. Med. Chem.*, 2011, **46**, 5175.

2 M. Lopalco, E. N. Koini, J. K. Cho and M. Bradley, *Org. Biomol. Chem.*, 2009, **7**, 856.

3 V. Wycisk, K. Achazi, O. Hirsch, C. Kuehne, J. Dernedde, R. Haag and K. Licha, *ChemistryOpen*, 2017, **6**, 437.

4 Z. Li, P. Praveen, *Fluorescent Dyes*, 2013, WO 2013/055647 A1.

5 F. Neese, *Wiley Interdiscip. Rev.: Comput. Mol. Sci.*, 2018, **8**, e1327.

6 Y. Xiong, A. Vargas Jentzsch, J. M. Osterrieth, E. Sezgin, I. V. Sazanovich, K. Reglinski, S. Galiani, A. W. Parker, C. Eggeling and H. L. Anderson, *Chem. Sci.*, 2018, **9**, 3029.

7 T. B. Demissie, K. Ruud and J. H. Hansen, *Organometallics*, 2015, **34**, 4218.

8 N. G. Connelly and W. E. Geiger, *Chem. Rev.*, 1996, **96**, 877.

9 A. Weller, *Pure Appl. Chem.*, 1968, **16**, 115.

10 F. Schneider, D. Waithe, M. P. Clausen, S. Galiani, T. Koller, G. Ozhan, C. Eggeling and E. Sezgin, *Mol. Biol. Cell*, 2017, **28**, 1507.

11 B. Roubinet, M. L. Bossi, P. Alt, M. Leutenegger, H. Shojaei, S. Schnorrenberg, S. Nizamov, M. Irie, V. N. Belov and S. W. Hell, *Angew. Chem. Int. Ed.*, 2016, **55**, 15429.

12 O. Nevskyi, D. Sysoiev, J. Dreier, S. C. Stein, A. Oppermann, F. Lemken, T. Janke, J. Enderlein, I. Testa, T. Huhn and D. Wöll, *Small*, 2018, **14**, 1703333.

13 M. Hofmann, C. Eggeling, S. Jakob and S. W. Hell, *Proc. Natl. Acad. Sci. U.S.A.*, 2005, **102**, 17565.

14 D. Waithe, M. P. Clausen, E. Sezgin and C. Eggeling, *Bioinformatics*, 2015, **32**, 958.

15 K. Pinkwart, F. Schneider, M. Lukoseviciute, T. Sauka-Spengler, E. Lyman, C. Eggeling and E. Sezgin, *J. Biol. Chem.*, 2019, **294**, 12599.

13. Author Contributions

A.T.F. and V.W. synthesized and characterized all compounds and recorded the photophysical data of the synthesized substances. A.T.F., V.W. and Y.X. prepared the synthetic vesicles and performed the microscopy experiments. S.G. and I.U. aligned the lasers on the RESOLFT microscope and assisted in the development of the RESOLFT microscopy sequence. I.U. and E.S. assisted with FCS experiments. V.W., A.T.F., I.U. and E.S. processed the FCS data. A.T.F. and K.G.L. carried out electrochemistry measurements. K.G.L. carried out DFT calculations. A.V.J. assisted with the fatigue modeling. H.L.A. and C.E. managed the project and acquired the funding. A.T.F., V.W. and H.L.A. wrote the manuscript. All authors discussed the experimental results and contributed to the editing of the manuscript.
